# Supplementary material for: Discovery of non-squalene triterpenes
Source: Nature. 2022 Jun 1;606(7913):414–9. doi: 10.1038/s41586-022-04773-3 (PMC9177416; doi:10.1038/s41586-022-04773-3)
Supplement: Supplementary file 1 — This file contains Supplementary Methods, Supplementary Figs. 1–59, Supplementary Tables 1–9, Supplementary Schemes 1 and 2, the synthetic procedure for compounds S1–S10, the synthetic procedure for compounds S11–S15, the amino acid sequences for AlphaFold2 prediction and supplementary references. [file 41586_2022_4773_MOESM1_ESM.docx]

**Supplementary Information**

**Discovery of non-squalene triterpenes**

Hui Tao^1,9^, Lukas Lauterbach^2,9^, Guangkai Bian^3,9^, Rong Chen^4,9^, Anwei Hou^2,9^, Takahiro Mori^1,5,6,9^, Shu Cheng^7^, Ben Hu^7^, Li Lu^7^, Xin Mu^7^, Min Li^7^, Naruhiko Adachi^8^, Masato Kawasaki^8^, Toshio Moriya^8^, Toshiya Senda^8^, Xinghuan Wang^4^, Zixin Deng^7^, Ikuro Abe^1,5^*, Jeroen S. Dickschat^2^* and Tiangang Liu^3,4,7^*

^1^Graduate School of Pharmaceutical Sciences, The University of Tokyo, 7-3-1 Hongo, Bunkyo-ku, Tokyo 113-0033, Japan.

^2^Kekulé-Institut für Organische Chemie und Biochemie, Rheinische Friedrich-Wilhelms-Universität Bonn, Gerhard-Domagk-Straße 1, 53121 Bonn, Germany.

^3^Department of Urology, Zhongnan Hospital of Wuhan University, School of Pharmaceutical Sciences, Wuhan University, Wuhan, 430071, China

^4^Department of Urology, Zhongnan Hospital of Wuhan University, Wuhan, 430071, China.

^5^Collaborative Research Institute for Innovative Microbiology, The University of Tokyo, Yayoi 1-1-1, Bunkyo-ku, Tokyo 113-8657, Japan.

^6^PRESTO, Japan Science and Technology Agency, Saitama, Japan.

^7^Key Laboratory of Combinatorial Biosynthesis and Drug Discovery, Ministry of Education and School of Pharmaceutical Sciences, Wuhan University, Wuhan, 430071, China.

^8^Structural Biology Research Center, Institute of Materials Structure Science, High Energy Accelerator Research Organization (KEK), 1-1 Oho, Tsukuba, Ibaraki 305-0801, Japan.

^9^These authors contributed equally: Hui Tao, Lukas Lauterbach, Guangkai Bian, Rong Chen, Anwei Hou, Takahiro Mori. Email: [abei@mol.f.u-tokyo.ac.jp](mailto:abei@mol.f.u-tokyo.ac.jp); [dickschat@uni-bonn.de](mailto:dickschat@uni-bonn.de); [liutg@whu.edu.cn](mailto:liutg@whu.edu.cn)

**Table of contents:**

| Supplementary Methods | Page 4-11 |
| --- | --- |
| Supplementary Figure 1 | Page 16 |
| Supplementary Figure 2 | Page 17 |
| Supplementary Figure 3 | Page 18 |
| Supplementary Figure 4 and 5 | Page 19 |
| Supplementary Figure 6 and 7 | Page 22 |
| Supplementary Figure 8 and 9 | Page 23 |
| Supplementary Figure 10 and 11 | Page 24 |
| Supplementary Figure 12 | Page 25 |
| Supplementary Figure 13 | Page 27 |
| Supplementary Figure 14 | Page 28 |
| Supplementary Figure 15 | Page 29 |
| Supplementary Figure 16 | Page 36 |
| Supplementary Figure 17 | Page 37 |
| Supplementary Figure 18 | Page 38 |
| Supplementary Figure 19 | Page 39 |
| Supplementary Figure 20 | Page 40 |
| Supplementary Figure 21 and 22 | Page 41 |
| Supplementary Figure 23 | Page 42 |
| Supplementary Figure 24 and 25 | Page 44 |
| Supplementary Figure 26 and 27 | Page 45 |
| Supplementary Figure 28 and 29 | Page 46 |
| Supplementary Figure 30 | Page 47 |
| Supplementary Figure 31 | Page 48 |
| Supplementary Figure 32 | Page 49-53 |
| Supplementary Figure 33 | Page 54 |
| Supplementary Figure 34 | Page 55 |
| Supplementary Figure 35 | Page 56 |
| Supplementary Figure 36 | Page 61-62 |
| Supplementary Figure 37 | Page 63 |
| Supplementary Figure 38 | Page 64 |
| Supplementary Figure 39 | Page 65 |
| Supplementary Figure 40 | Page 66 |
| Supplementary Figure 41 and 42 | Page 67 |
| Supplementary Figure 43 | Page 68 |
| Supplementary Figure 44 | Page 69 |
| Supplementary Figure 45 and 46 | Page 70 |
| Supplementary Figure 47 | Page 71 |
| Supplementary Figure 48 and 49 | Page 74 |
| Supplementary Figure 50 and 51 | Page 75 |
| Supplementary Figure 52 and 53 | Page 76 |
| Supplementary Figure 54 | Page 77 |
| Supplementary Figure 55 | Page 78 |
| Supplementary Figure 56 | Page 79 |
| Supplementary Figure 57 and 58 | Page 80 |
| Supplementary Figure 59 | Page 81 |
| Supplementary Table 1 | Page 12 |
| Supplementary Table 2 | Page 13-15 |
| Supplementary Table 3 | Page 20 |
| Supplementary Table 4 | Page 21 |
| Supplementary Table 5 | Page 26 |
| Supplementary Table 6 | Page 43 |
| Supplementary Table 7 | Page 69 |
| Supplementary Table 8 | Page 72 |
| Supplementary Table 9 | Page 73 |
| Supplementary Scheme 1 | Page 30 |
| Supplementary Scheme 2 | Page 57 |
| Synthetic procedure compounds **S1**-**S10** | Page 31-34 |
| Synthetic procedure for compounds **S11**-**S15** | Page 57-60 |
| Amino acid sequences for AlphaFold2 prediction | Page 82-84 |
| Supplementary References | Page 84-86 |

**Supplementary Methods**

**GC-EI-MS and GC-MS-QtoF.** GC-EI-MS analyses were performed using different machines in the laboratories at Wuhan University, the University of Bonn, and the University of Tokyo. Wuhan: A Thermo Trace GC Ultra-TSQ Quantum XLS harboring a TRACE TR-5MS (30 m × 0.25 mm × 0.25 μm) column was used. Temperature profile: oven temperature, 80 °C for 1 min, increased to 220 °C at a rate of 10 °C/min, and held at 220 °C for 15 min; injector and transfer lines, maintained at 230 and 240 °C, respectively. The Thermo Xcalibur 2.2 was used for data acquisition and processing. Bonn: A 7890B GC–5977A mass detector system (Agilent) was used. The GC was fitted with an HP5-MS fused silica capillary column (30 m, 0.25 mm i.d., 0.50 μm film). The GC parameters were as follows: inlet pressure, 77.1 kPa (He flow at 23.3 ml/min); injection volume, 2 μl; temperature ramp, 5 min at 50 °C, increasing 5 °C/min to 320 °C; valve time, 60 s; carrier gas, He at 1.2 ml/min. The MS parameters were as follows: ion source, 230 °C; transfer line, 250 °C; quadrupole, 150 °C; and electron energy, 70 eV. Retention indices (*I*) were determined in comparison to a homologous series of *n*-alkanes (C_7_–C_40_). The Agilent MSD ChemStation D02.00.237 was used for data acquisition and processing. Tokyo: A GC/MS-QP2010 Plus (Shimadzu) spectrometer was used, fitted with a Restek Rtx-5MS glass capillary column (0.25 mm i.d. × 30 m, 0.25 μm film thickness). The temperature of the ionization chamber was 260 °C, with electron impact ionization at 70 eV. Helium was used as a carrier gas at a linear velocity of 43.0 cm/min. The following condition was used for the analysis: 100 °C for 1 min, temperature increase from 100 to 250 ºC at the rate of 10 °C/min, and 250 °C for 15 min. The Shimadzu GCMS solution software (version 4.41) was used for data acquisition and processing. GC-MS-QToF analyses were carried out on a 7890 B GC–7200 accurate-mass Q-ToF detector system (Agilent). The GC was equipped with an HP5-MS fused silica capillary column (30 m, 0.25 mm i.d., 0.50 μm film). The GC parameters were as follows: injection volume, 1 μl; split ratio, 10:1; valve time, 60 s; carrier gas, He at 1 ml/min; and temperature ramp, 5 min at 50 °C, increasing 10 °C/min to 320 °C. The MS parameters were as follows: inlet pressure, 83.2 kPa (He at 24.6 ml/min); transfer line, 250 °C; and electron energy, 70 eV.

**Reverse transcription experiments.** To verify the transcription of *TvTS*, native host *T. verruculosus* and the engineered strain RC177 were cultivated in PDB medium at 30 °C for two days with three replicates. Followed, 100 mg mycelium was harvested and total RNA was extracted using a RNeasy plant Mini kit (QIAGEN, Germantown, MD, USA) as previously described^1^. An RNase-Free DNase Set (QIAGEN) was employed to eliminate DNA contamination. Followed, a total amount of 1 µg RNA was selected as reverse transcription template and cDNA was synthesized using a PrimeScript Reagent kit (TaKaRa, Dalian, China). Finally, using primer pair P9/P10 to detect the transcription of *TvTS* in the native host and the engineered *T. verruculosus* RC177.

**Construction of *T. verruculosus* RC177.** The full-length gene coding for TvTS was amplified from *T. verruculosus* TS63-9 using the primer pair P9/P10, *Tnos* was amplified from pGB92^1^ with the primer pair P11/P12, the plasmid backbone was amplified from pSC134 with the primer pair P9/P10, and *Pamy*, *HygR* and *AMA1* were amplified from pSC269 using the primer pairs P15/P16, P17/P18, and P19/P20, respectively. All the obtained fragments were assembled using a yeast assembly method^2^ to produce plasmid pRC177. Followed, pRC177 was transformed into *T. verruculosus* TS63-9 via the protoplast transformation method to produce *T. verruculosus* RC177 for functional verification of TvTS in vivo. Protoplasts were produced according to a previously described method^3^, with some modifications. *T. verruculosus* was transferred to a 250-ml shake flask with 50 ml PDB medium and cultivated at 25 °C and 180 rpm for 5 days. The mycelium was harvested by centrifugation (8,000 × *g*, 10 min) at 4 °C and washed twice with osmotic medium (1.2 M MgSO_4_, 10 mM NaH_2_PO_4_, pH 5.8). The mycelium was digested with lysis solution (20 g/l yatalase, 30 g/l lysing enzyme, 10 g/l snailase) at 34 °C and 80 rpm for 7–8 h. The protoplasts were filtered through sterile gauze, mixed with an equal volume of trapping buffer (0.4 M sorbitol in 100 mM Tris-HCl, pH 7.0), inverted slowly to mix well, harvested by centrifugation (3000 rpm, 10 min) at 4 °C, and washed with STC buffer (1.2 M sorbitol, 10 mM CaCl_2_, 10 mM Tris-HCl, pH 7.5) twice. The concentration of protoplasts was adjusted to 10^8^–10^9^ for plasmid transformation. Then 10–15 μg plasmid was dissolved in 50 μl STC buffer, transformed into 150 μl protoplast preparation, mixed, and incubated on ice for 1 h. Subsequently, 1.25 ml of 25% PEG6000 solution (250 g/l PEG6000, 100 mM CaCl_2_, 0.6 M KCl, 50 mM Tris-HCl, pH 7.5) was added, and the sample was mixed well and incubated at room temperature for 30 min. The protoplast solution was then moved to a 15 ml centrifuge tube, 4 ml STC buffer was added, and the sample was mixed well. Finally, 1 ml protoplast solution was coated onto regeneration medium (glucose minimal medium with 1.2 M sorbitol and 200 mg/l hygromycin B) and incubated at 30 °C for 3–4 days to obtain transformants. The transformants were verified by polymerase chain reaction amplification using primer pair P9/P14 and Phusion DNA polymerase (New England Biolabs, Beverly, MA, USA). Fermentation of *T. verruculosus* RC177 was carried out in PDB medium supplemented with 2% maltose (to induce the *amyB* promoter and activate TvTS) at 30 °C and 140 rpm for 7 days, and the resulting solution was extracted with hexane/ethyl acetate (4/1) for GC-MS analysis.

**Cryo-EM data processing.** For the MpMS-PT dataset, 2,522 particles were manually picked and performed 2D classification for preparing a 2D reference of template-matching-based auto-pick by RELION-3.1^57^. From the 1,888 micrographs, 904,572 particles were automatically picked and extracted while rescaling to 2.64 Å/pixel with 100-pixel box size. The extracted particle images were split into 8 sets and one of the sets was subjected to the reference-free 2D classification for single cycle (200 expected classes, 180 Å mask diameter). After the single cycle of reference-free 2D classification, the 89,598 particles corresponding to the best 9 classes, which displayed secondary-structural elements, were selected from the result, and then used for *ab initio* reconstruction (asymmetry, single expected class, 200 Å mask diameter). The extracted 904,572 particle images were split into 8 sets again and subjected to the reference-free 2D classification for two cycles (200 expected classes, 180 Å mask diameter). After the two cycles of reference-free 2D classification, the 878,822 particles corresponding to the best 14 classes, which displayed secondary-structural elements, were selected from the result. For the analysis without symmetry, the generated *ab initio* map was used as an initial 3D reference for the first 3D classification (4 expected classes, 200 Å mask diameter). The 3D volume and 431,246 particles of the best 3D class, which displayed the highest resolution, were used for the first 3D refinements (*C_1_* symmetry, 200 Å mask diameter). The refined volume and 431,246 particle images were rescaled to 0.88 Å/pixel with a 300-pixel box size and used for the 3D refinement (*C_1_* symmetry, 200 Å mask diameter). The generated 3D refined map was used as a 3D reference for the second 3D classification (4 expected classes, 200 Å mask diameter). The 3D volume and 325,729 particles of the best 3D class, which displayed the highest resolution, were used for the subsequent 3D refinements (*C_1_* symmetry, 200 Å mask diameter). The generated 3D refined map and 325,729 particles was used for the no-alignment 3D classification (2 expected classes, T=4, 200 Å mask diameter). The 3D volume and 325,729 particles of the best 3D classes, which displayed the highest resolution, were re-extracted as 0.88 Å/pixel with 400-pixel box size and used for the subsequent 3D refinements (*C_1_* symmetry, 300 Å mask diameter). The cycle of CTF refinement and Bayesian polishing was performed^4^. The 3D refinement (*C_1_* symmetry, 300 Å mask diameter) with a soft-edged 3D mask (15-pixel extension, 30-pixel soft cosine edge) was executed after each CTF refinement and Bayesian polishing step. The last 3D refinement (*C_1_* symmetry, 300 Å mask diameter) with a soft-edged 3D mask (15-pixel extension, 30-pixel soft cosine edge) and post-processing generated the final result at 3.83 Å resolution.

For the analysis with *D_3_* symmetry, *D_3_* symmetry was imposed on the first 3D refined map, which was used for the 3D refinement (*D_3_* symmetry, 200 Å mask diameter). The refined volume and 431,246 particle images were rescaled to 0.88 Å/pixel with a 300-pixel box size and used for the 3D refinement (*D_3_* symmetry, 200 Å mask diameter). The generated 3D refined map was used as a 3D reference for the 3D classification (4 expected classes, 200 Å mask diameter). The 3D volume and 295,009 particles of the best 3D class, which displayed the highest resolution, were used for the subsequent 3D refinements (*D_3_* symmetry, 200 Å mask diameter). The generated 3D refined map and 295,009 particles was used for the no-alignment 3D classification (2 expected classes, T=4, 200 Å mask diameter). The 3D volume and 132,926 particles of the best 3D classes, which displayed the highest resolution, were re-extracted as 0.88 Å/pixel with 400-pixel box size and used for the subsequent 3D refinements (*D_3_* symmetry, 300 Å mask diameter). The cycle of CTF refinement and Bayesian polishing was repeated two times^58^. The 3D refinement (*D_3_* symmetry, 300 Å mask diameter) with a soft-edged 3D mask (15-pixel extension, 30-pixel soft cosine edge) was executed after each CTF refinement and Bayesian polishing step. The last 3D refinement (*D_3_* symmetry, 300 Å mask diameter) with a soft-edged 3D mask (15-pixel extension, 30-pixel soft cosine edge) and post-processing generated the final result at 3.17 Å resolution.

**Cryo-EM data processing for the MpMS-crosslink.** For the MpMS-crosslink dataset, 1,527 particles were manually picked and performed 2D classification for preparing a 2D reference of template-matching-based auto-pick by RELION-3.1. From the 1,529 micrographs, 573,736 particles were automatically picked and extracted while rescaling to 2.64 Å/pixel with 100-pixel box size. The extracted particle images were split into 6 sets and one of the sets was subjected to the reference-free 2D classification for single cycle (200 expected classes, 220 Å mask diameter). After the single cycle of reference-free 2D classification, the 42,751 particles corresponding to the best 10 classes, which displayed secondary-structural elements, were selected from the result, and then used for *ab initio* reconstruction (asymmetry, single expected class, 240 Å mask diameter). The extracted 573,736 particle images were split into 6 sets again and subjected to the reference-free 2D classification for two cycles (200 expected classes, 220 Å mask diameter). After the two cycles of reference-free 2D classification, the 555,447 particles corresponding to the best 28 classes, which displayed secondary-structural elements, were selected from the result.

For the analysis without symmetry, the generated *ab initio* map was used as an initial 3D reference for the 3D classification (4 expected classes, 220 Å mask diameter). The 3D volume and 282,791 particles of the best 3D class, which displayed the highest resolution, were used for the 3D refinements (*C_1_* symmetry, 220 Å mask diameter). The refined volume and 282,791 particle images were rescaled to 0.88 Å/pixel with a 300-pixel box size and used for the no-alignment 3D classification (2 expected classes, T=4, 220 Å mask diameter). The 3D volume and 113,860 particles of the best 3D classes, which displayed the highest resolution, were used for the third 3D refinements (*C_1_* symmetry, 220 Å mask diameter). The cycle of CTF refinement and Bayesian polishing was repeated four times^58^. The 3D refinement (*C_1_* symmetry, 220 Å mask diameter) with a soft-edged 3D mask (15-pixel extension, 30-pixel soft cosine edge) was executed after each CTF refinement and Bayesian polishing step. The last 3D refinement (*C_1_* symmetry, 220 Å mask diameter) with a soft-edged 3D mask (15-pixel extension, 30-pixel soft cosine edge) and post-processing generated the final result at 4.00 Å resolution. The final map was z-flipped after the post-processing.

For the analysis with *D_3_* symmetry, *D_3_* symmetry was imposed on the third 3D refined map and the 555,447 particles were re-extracted as 0.88 Å/pixel with a 300-pixel box size, which was used as an initial 3D reference for the 3D classification (4 expected classes, 220 Å mask diameter). The 3D volume and 181,017 particles of the best 3D class, which displayed the highest resolution, were used for the 3D refinement (*D_3_* symmetry, 220 Å mask diameter). The refined volume and 181,017 particles were used for the no-alignment 3D classification (2 expected classes, T=32, 220 Å mask diameter). The 3D volume and 69,658 particles of the best 3D classes, which displayed the highest resolution, were used for the subsequent 3D refinements (*D_3_* symmetry, 220 Å mask diameter). The cycle of CTF refinement and Bayesian polishing was repeated three times. The 3D refinement (*D_3_* symmetry, 220 Å mask diameter) with a soft-edged 3D mask (15-pixel extension, 30-pixel soft cosine edge) was executed after each CTF refinement and Bayesian polishing step. The last 3D refinement (*D_3_* symmetry, 220 Å mask diameter) with a soft-edged 3D mask (15-pixel extension, 30-pixel soft cosine edge) and post-processing generated the final result at 3.47 Å resolution. The final map was z-flipped after the post-processing.

After each of the 3D refinements, the “gold-standard” FSC resolution with the 0.143 criterion^5^ in RELION was used as a global resolution estimation with phase randomization, to account for possible artifactual resolution enhancement caused by solvent mask^6,7^. The local resolutions of the 3D cryo-EM maps were estimated using RELION’s own implementation. UCSF Chimera 13.1^8^ was used for the visualization of the output 3D images. The cryo-EM maps and the atomic coordinates for MpMS-PT domain and MpMS-crosslink have been deposited in Electron Microscopy Data Bank (EMDB, https://www.ebi.ac.uk/pdbe/emdb/) and PDB with accession codes EMD-32531 and EMD-32532, and 7WIJ, respectively*.* The statistics of EM data processing and refinement are summarized in Extended Data Table 2.

**Structural characterization of compound 1-3.** NMR measurements were performed on a Bruker (Billerica, MA, USA) Avance III HD Cryo (700 MHz) and a Bruker Avance III HD Prodigy (500 MHz), and analyzed using the MestReNova 5.3.1. The spectra were referenced against solvent signals (^1^H-NMR, residual proton signal: C_6_D_6_ *δ* = 7.16 ppm, ^13^C-NMR: C_6_D_6_ *δ* = 128.06 ppm).

**Talaropentaene, (3*aR*,6*E*,10*E*,12*aS*)-3-((*E*)-6,10-dimethylundeca-5,9-dien-2-yl)-6,10,12a-trimethyl-1,3a,4,5,8,9,12,12a-octahydrocyclopenta[11]annulene (1).** Yield: 7.2 mg, obtained as a colourless oil. Optical rotation: [α]^22^_D_ = –4.4 (*c* 0.26, CHCl_3_). HRESI-MS: *m*/*z* = 409.3829 [M+H]^+^ (C_30_H_48_), calculated: 409.3828.

**Macrophomene, (1*R*,2*E*,6*E*,10*E*,14*E*,18*E*,22*R*)-3,7,11,15,19,23,23-heptamethylbicyclo [20.1.0] tricosa-2,6,10,14,18-pentaene (2).** Yield: 0.6 mg (1.5 μmol, 2 %). *R*_f_ (pentane) = 0.65. [α]_D_^25^ = 0.65 (*c* 0.45, C_6_D_6_). HRMS (APCI): *m*/*z* = 409.3829 (calc. for [C_30_H_49_]^+^ 409.3829). GC (HP5-MS): *I* = 2872. MS (EI, 70 eV): *m*/*z* (%) = 408 (4), 393 (2), 365 (1), 341 (2), 281 (5), 257 (4), 229 (4), 203 (8), 189 (16), 175 (12), 161 (27), 147 (29), 135 (39), 121 (88), 107 (100), 93 (98), 81 (89), 67 (81), 55 (39), 41 (19), see Supplementary Figure 22. IR (diamond ATR): $\tilde{\text{}}$ / cm^‑1^ = 2919 (s), 2853 (m), 2277 (w), 1737 (w), 1666 (w), 1565 (w), 1443 (m), 1380 (m), 1343 (w), 1260 (w), 1156 (w), 1096 (m), 1024 (m), 975 (w), 912 (m), 747 (w), 585 (w), 543 (s), 496 (s), 439 (m). NMR data are given in Supplementary Table 6 and spectra are shown in Supplementary Figures 24-30.

**Colleterpenol** **(*S,E*)-6,10-dimethyl-2-((*R,3E,7E,11E*)-4,8,12-trimethylcyclotetradeca-3,7,11-trien-1-yl)undeca-5,9-dien-2-ol (3).** [α]^20^_D_ = –20.7 (*c* 0.34, CHCl_3_), was obtained as a colorless oil. The molecular formula of **3** was determined as C_30_H_50_O based on its HRESI-MS: *m*/*z* = 449.3751 [M + Na]^+^ (C_30_H_50_O), calculated: 449.3754, see Supplementary Figure 54, implying six degrees of unsaturation. The ^1^H NMR (400 MHz, CDCl_3_) spectrum exhibited characteristic signals for seven methyl groups at *δ*_H_ 1.59 (m, H-24), *δ*_H_ 1.59 (m, H-25), *δ*_H_ 1.59 (m, H-26), *δ*_H_ 1.16 (m, H-27), *δ*_H_ 1.65 (s, H-28), *δ*_H_ 1.62 (s, H-29), *δ*_H_ 1.70 (s, H-24), and five olefinic protons at *δ*_H_ 5.15 (m, H-2), *δ*_H_ 4.97 (m, H-6), *δ*_H_ 5.04 (m, H-10), *δ*_H_ 5.16 (m, H-18), *δ*_H_ 5.11 (s, H-22). The ^13^C and DEPT spectra (100 MHz, CDCl_3_) of compound **3** suggested the presence of 30 carbon resonances including seven methyls, eleven methylenes, six methines (five olefinic carbons), and six quaternary carbons (one oxygenated, five olefinic carbons) (Supplementary Table 8). These data suggest the monocyclic skeleton for compound **3**. The ^1^H-^1^H COSY correlations then revealed the spin systems of H-1/H-2/H-14/H-13/H-12, H-4/H-5/H-6, H-8/H-9/H-10, H-16/H-17/H-18, and H-20/H-21/H-22. Additionally, HMBC correlations from Me-30 and Me-29 to C-22, C-23 and C-29; from H-20 to C-18, C-22 and C-28; from H-17 to C-15, C-18 and C-19; from Me-27 to C-14, C-15 and C-16; from H-1 to C-15, C-13 and C-3; from Me-24 to C-2, C-3 and C-4; from H-6 to C-4, C-5, C-8 and C-25; from H-8 to C-6 and C-10; from to Me-26 to C-12 and C-10; from H-12 to C-10 and C-14; from H-14 to C-2, C-12 and C-16. Thus, the planar structure of compound **3** was identified as a 14- macrocyclic triterpenol.

**Relative configuration of colleterpenol (3)**. To reveal the relative configuration of colleterpenol (**3**), we ran quantum NMR calculations on its two possible diastereoisomers, (14*R**,15*S**)-**3** and (14*R**,15*R**)-**3**. The results were represented with parameters including correlation coefficient (*R*^2^), mean absolute error (MAE), corrected mean absolute error (CMAE), and the DP4+ probability^9^. The two diastereoisomers possessed identical *R*^2^ (0.9983) and similar MAE (2.1 for 14*R**,15*S**, 2.3 for 14*R**,15*R**), and CMAE (1.6 for 14*R**,15*S**, 1.5 for 14*R**,15*R**) in carbon data analysis. In proton data analysis, (14*R**,15*S**)-**3** showed better correlations in *R*^2^ (0.9849 for 14*R**,15*S**, 0.9757 for 14*R**,15*R**), and smaller differences in MAE (0.17 for 14*R**,15*S**, 0.20 for 14*R**,15*R**) and CMAE (0.11 for 14*R**,15*S**, 0.13 for 14*R**,15*R**). Additionally, the DP4+ probability showed significant differences in all data analysis (100% for 14*R**,15*S**, 0% for 14*R**,15*R**) (Supplementary Table 9). We further conducted DP4+ probability analysis based on random conformational amplitudes^10^ to confirm the configuration of **3**. As a result, the averaged probability of (14*R**,15*S**)-**3** and (14*R**,15*R**)-**3** were 70.10% and 29.9%, respectively, (Supplementary Figure 45) which was consistent with the result obtained by standard DP4+. These results indicated that (14*R**,15*S**)-**3** was the real structure of **3**. The absolute configuration of **3** was determined by TDDFT calculation of ECD spectrum which was performed at Cam-B3LYP/6-31+G (2d, p) level of theory with PCM in methanol. The calculated ECD spectrum matched the experimental counterpart well (Supplementary Figure 46), thus, the absolute configuration of **3** could be determined as 14*R*,15*S*.

**Quantum chemical calculation of NMR shielding tensors and ECD spectrum.** The conformational search of **(14*R**,15*S**)-3** and **(14*R**,15*R**)-3** were undertaken with the CREST code (version 2.8) using the default iMTD-GC procedure^11^ Top 45 conformers of both **(14*R**,15*S**)-3** and **(14*R**,15*R**)-3** were subjected to geometry optimization and subsequent frequency analysis in Gaussian 09 software package^12^ at B3LYP/6-31G(d) level in gas phase. More accurate energies of optimized conformers were evaluated at M06-2X-D3/6-311+G(2d,p) level of theory in the gas phase, and were then added to “thermal correction to Gibbs free energies” obtained by frequency analysis to get the Gibbs free energy of each conformer. Those two B3LYP geometries with RMSD below 0.15 Å and energy difference below 0.15 kcal were regarded as duplicate conformers, and the one with higher energy was removed. Subsequently, Room-temperature (298.15 K) equilibrium populations were calculated according to Boltzmann distribution law:

$$p_{i}= \frac{n_{i}}{\sum_{j} n_{j}}= \frac{e^{-\Delta G_{i}/RT}}{\sum_{j} e^{-\Delta G_{j}/RT}}$$

Where *P_i_* is the population of the *i^th^* conformer; *n_i_* the number of molecules in *i^th^* conformer; *ΔG* is the relative Gibbs free energy (kcal/mol); *T* is room temperature (298.15 K); *R* is the ideal gas constant (0.0019858995).

After the population analysis, 39 non-redundant conformers remained for both **(14*R**,15*S**)-3** and **(14*R**,15*R**)-3**, and their coordinates were provided below. In order to perform both standard DP4+ analysis and DP4+ analysis bases on random conformational amplitudes, all non-redundant conformers were further subjected to calculation of NMR shielding tensors at the mPW1PW91/6-31+G(d,p) level with PCM model in chloroform. The obtained shielding constants were converted into chemical shifts by referencing to TMS at 0 ppm (*δ*_cal_ = *σ*_TMS_ – *σ*_cal_), where the *σ*_TMS_ was the shielding constant of TMS calculated at the same level of theory. The parameters *a* and *b* of the linear regression *δ*_cal_ = *aδ*_exp_ + *b*; the correlation coefficient, *R*^2^; the mean absolute error (MAE) defined as Σ_n_|*δ*_cal_ – *δ*_exp_|/*n*; the corrected mean absolute error (CMAE) defined as Σ_n_|*δ*_corr_ – *δ*_exp_|/*n*, where *δ*_corr_ = (*δ*_cal_ – *b*)/*a* were calculated. The optimized conformation geometries and populations of all conformations were provided in Supplementary Tables 10 to 111.

Those conformers with Boltzmann distribution over 1% were subjected to theoretical calculation of the ECD spectrum, and the calculation was performed in Gaussian 09 software package using TDDFT at CAM-B3LYP/6-31+G(2d,p) level of theory with PCM in methanol. The ECD spectrum of **(14*R*,15*S*)-3** was obtained by weighing the Boltzmann distribution of each conformation in Multiwfn 3.8^13^.

The ECD spectrum is simulated by overlapping Gaussian functions for each transition according to

$$\Delta\varepsilon\left( E \right)=\frac{1}{2.297\times{10}^{-39}}\times\frac{1}{\sqrt{2\pi\sigma}}\sum_{i}^{A} \Delta E_{i}R_{i}e^{-{[(E-E_{i})/(2\sigma)]}^{2}}$$

Where *σ* represents the width of the band at 1/*e* height, and Δ*E_i_* and *R_i_* are the excitation energies and rotational strengths for transition *i*, respectively. *σ* = 0.66 eV and R^velocity^ have been used in this work.

**Supplementary Table 1 │ Strains and plasmids used in this study.**

| **Strain** | **Relevant genotyp** | **Reference** |
| --- | --- | --- |
| BL21(DE3) | *E. coli B F^-^dcm ompT hsdSB(rB^-^mB^-^)gal* | Invitrogen |
| YZL141 | *S. cerevisiae*:: pGAL10-tHMG1 | ^14^ |
| XM018 | *S. cerevisiae*:: P_GAL1_-MPH_02178 (MpMS)-T_ADH1_ | this work |
| XM139 | *S. cerevisiae*:: P_GAL1_-ZTR_06220 (TvTS)-T_ADH1_ | this work |
| TS63-9 | *T. verruculosus* | ^15^ |
| MS6 | *Macrophomina phaseolina* | ^16^ |
| RC177 | *T. verruculosus*:: Hyg^R^, PamyB- ZTR_06220 (TvTS)-Tnos | this work |
| RC181 | *S. cerevisiae*:: P_GAL1_-Cgl13855 (CgCS)-T_ADH1_ | this work |
| **Plasmid** | **Description** | **Reference** |
| pXM018 | p426gal derived, *URA*, P_GAL1_-MpMS-T_ADH1_ | this work |
| pXM139 | p426gal derived, *URA*, P_GAL1_-TvTS-T_ADH1_ | this work |
| pRC009 | pET28a derived, C-terminal His_6_-tag, for expression of PT domain of TvTS in *E. coli* BL21(DH3) | this work |
| pRC041 | pET21a derived, N-terminal His_6_-tag, for expression of TC domain of TvTS in *E. coli* BL21(DH3) | this work |
| pRC088 | pET28a derived, C-terminal His_6_-tag, for expression of full lenght MpMS in *E. coli* BL21(DH3) | this work |
| pRC088-D114A-N115A | pET28a derived, C-terminal His_6_-tag, for expression of TC domain inactivated MpMS-D114A-N115A in *E. coli* BL21(DH3) | this work |
| pRC177 | pSC149 derived, Hyg^R^, P_amyB_-TvTS-T_NOS_ | this work |
| pSUMO041 | pET28a derived, P_T7_: N-terminal His_6_- and SUMO-tag, TvTS-TC (N-terminal 328 AA) | this work |
| pMBP139 | pET28a derived, P_T7_: N-terminal His_6_- and MBP-tag, TvTS | this work |
| pRC181 | p426gal derived, *URA*, P_GAL1_-CgCS(Cgl13855)-T_ADH1_ | this work |
| pYJ117-PTTC027 | p426gal derived, *URA*, P_GAL1_-PTTC027-T_ADH1_ | this work |
| pYJ117-PTTC044 | p426gal derived, *URA*, P_GAL1_-PTTC044-T_ADH1_ | this work |
| pYJ117-PTTC060 | p426gal derived, *URA*, P_GAL1_-PTTC060-T_ADH1_ | this work |
| pYJ117-PTTC114 | p426gal derived, *URA*, P_GAL1_-PTTC0114-T_ADH1_ | this work |

**Supplementary Table 2 │ Primers used in this study.**

| Primer | Function | Sequence (5ʹ to 3ʹ)^[a]^ |
| --- | --- | --- |
| P1 | Expression TvTS-PT | GCTCGAATTCTTAACCCACAACCAGCAGCTCAACC |
| P2 | Expression TvTS-PT | CAGCCATATGCAGAAGCAATTTGTTGGTGAC |
| P3 | Expression TvTS-TC | CGACAAGCTTATGGACTTCAAGTACAGCCGTG |
| P4 | Expression TvTS-TC | ATATCTCGAGCGGGGTCTTAAAGTCAACGAAG |
| P5 | Expression MpMS-PT | GCAGCATATGTGCAACACCAAATGCTAC |
| P6 | Expression MpMS-PT | CCGCAATCGCCGCCATATAAAAGATGCTCTC |
| P7 | Expression MpMS-PT | CTTTTATATGGCGGCGATTGCGGAGAGCGGTAGCC |
| P8 | Expression MpMS-PT | GGCAGAATTCCTTACGCTTTAATACGCAGAC |
| P9 | pRC177 construction for TvTS | CTCTGAACAATAAACCCCACAGATGGATTTTAAATACTCTCGGGAAC |
| P10 | pRC177 construction | GTTTGAACGATCGGCGCGCCTCAACCAACTACCAGCAGCTCAACG |
| P11 | pRC177 construction | GAGCTGCTGGTAGTTGGTTGAGGCGCGCCGATCGTTCAAACATTTG |
| P12 | pRC177 construction | CTTCAATATCATCTTCTGGATCTAGTAACATAGATGACACCGCGCG |
| P13 | pRC177 construction | AATGGAAACGGTGAGAGTCCAGGCGCGCCTTTCCATAGGCTCCG |
| P14 | pRC177 construction | AAATGATCAAAACACCATGAACGCGCCCTGTAGCGGCGCA |
| P15 | pRC177 construction | TGCGCCGCTACAGGGCGCGTTCATGGTGTTTTGATCATTT |
| P16 | pRC177 construction | AGAGTATTTAAAATCCATCTGTGGGGTTTATTGTTCAGAGAAGGGAGAG |
| P17 | pRC177 construction | CGGTGTCATCTATGTTACTAGATCCAGAAGATGATATTGAAGGAGCAC |
| P18 | pRC177 construction | GGAACATATACTGGGCCCGGGATCTGGATTTTAGTACTGGATTTTG |
| P19 | pRC177 construction | CCAGTACTAAAATCCAGATCCCGGGCCCAGTATATGTTCCGCAG |
| P20 | pRC177 construction | GAGCCTATGGAAAGGCGCGCCTGGACTCTCACCGTTTCCATTGGGG |
| P21 | Expression TvTS | TATTTTCAGGGATCCATGGACTTCAAGTACAGCCGTG |
| P22 | Expression TvTS | TGCGGCCGCAAGCTTTTAACCCACAACCAGCAGCTC |
| P23 | Expression TvTS | CAGATTGGAGGTATGGACTTCAAGTACAGCCGTG |
| P24 | Expression TvTS | TGGTGCTCGAGTCACGGGGTCTTAAAGTCAACGAAG |
| P25 | Expression TvTS | TTTAAGACCCCGTGACTCGAGCACCACCACCACCAC |
| P26 | Expression TvTS | CTTGAAGTCCATACCTCCAATCTGTTCGCGGTG |
| F65A-F | TvTS mutation | AGTTCGGTGCTGTTAGCGCGTGCATTCC |
| F65A-R | TvTS mutation | CGCTAACAGCACCGAACTGCGGGCCACG |
| F65L-F | TvTS mutation | TTCGGTCTTGTTAGCGCGTGCATTCC |
| F65L-R | TvTS mutation | GCGCTAACAAGACCGAACTGCGGGCC |
| F89A-F | TvTS mutation | TTCGCGGCTCTGCACGACGATATGACC |
| F89A-R | TvTS mutation | GTCGTGCAGAGCCGCGAACTCGTTCGCG |
| F89L-F | TvTS mutation | AGTTCGCGCTTCTGCACGACGATATG |
| F89L-R | TvTS mutation | GTCGTGCAGAAGCGCGAACTCGTTC |
| G184F-F | TvTS mutation | CGGATTGCTTCGAAAAATTCTGGTTTG |
| G184F-R | TvTS mutation | AATTTTTCGAAGCAATCCGCGCAAC |
| F187A-F | TvTS mutation | CGAAAAAGCCTGGTTTGGTCTGGTGACG |
| F187A-R | TvTS mutation | CAAACCAGGCTTTTTCGCCGCAATCCGC |
| F187L-F | TvTS mutation | GCGAAAAACTCTGGTTTGGTCTGGTG |
| F187L-R | TvTS mutation | CCAAACCAGAGTTTTTCGCCGCAATCC |
| W188A-F | TvTS mutation | AAATTCGCGTTTGGTCTGGTGACGTTCG |
| W188A-R | TvTS mutation | GACCAAACGCGAATTTTTCGCCGCAATC |
| W188L-F | TvTS mutation | AAAAATTCCTGTTTGGTCTGGTGACG |
| W188L-R | TvTS mutation | CAGACCAAACAGGAATTTTTCGCCGC |
| A212M-F | TvTS mutation | AGCGTCTGATGCAAAACGCGTACCTGGC |
| A212M-R | TvTS mutation | CGTTTTGCATCAGACGCTGCACCAGCTC |
| Y216W-F | TvTS mutation | AAACGCGTGGCTGGCGGCGGGCCTGACC |
| Y216W-R | TvTS mutation | CCGCCAGCCACGCGTTTTGCGCCAGACG |
| A219L-F | TvTS mutation | TACCTGGCGCTGGGCCTGACCAACGAC |
| A219L-R | TvTS mutation | GGTCAGGCCCAGCGCCAGGTACGCG |
| G220L-F | TvTS mutation | CTGGCGGCGCTCCTGACCAACGACCTG |
| G220L-R | TvTS mutation | GTTGGTCAGGAGCGCCGCCAGGTAC |
| G300M-F | TvTS mutation | GAAACCATGCTGTATGGCATCAGCGGTAG |
| G300M-R | TvTS mutation | CCATACAGCATGGTTTCCAGGTACGCCAG |
| G303S-F | TvTS mutation | GTCTGTATAGCATCAGCGGTAGCACCGC |
| G303S-R | TvTS mutation | GCTGATGCTATACAGACCGGTTTCCAGG |
| S307N-F | TvTS mutation | ATCAGCGGTAACACCGCGTGGAACCTGG |
| S307N-R | TvTS mutation | ACGCGGTGTTACCGCTGATGCCATACAG |
| S307F-F | TvTS mutation | ATCAGCGGTTTCACCGCGTGGAACCTG |
| S307F-R | TvTS mutation | ACGCGGTGAAACCGCTGATGCCATAC |
| W310A-F | TvTS mutation | CACCGCGGCGAACCTGGATTGCCCG |
| W310A-R | TvTS mutation | TCCAGGTTCGCCGCGGTGCTACCGC |
| V206F-F | MpMS mutation | TGCGATCTTCGCGGTTCACCTGATGAAC |
| V206F-R | MpMS mutation | GTGAACCGCGAAGATCGCACCGCTATCC |
| A207W-F | MpMS mutation | GATCGTGTGGGTTCACCTGATGAACTTC |
| A207W-R | MpMS mutation | AGGTGAACCCACACGATCGCACCGCTATC |
| L66D-F | MpMS mutation | GCCTCGCAGTCTTCTTTCGCCTTCGCGCTACCCG |
| L66D-R | MpMS mutation | GCGAAGGCGAAAGAAGACTGCGAGGCGCTGATCGG |
| C67W-F | MpMS mutation | CAGCGCCTCCCACAGTTCTTTCGCCTTCGCGC |
| C67W-R | MpMS mutation | GGCGAAAGAACTGTGGGAGGCGCTGATCGG |
| T78G-F | MpMS mutation | CTTTCGCAGCCCGGACGCGGATACACAC |
| T78G-R | MpMS mutation | GTATCCGCGTCCGGGCTGCGAAAGCGCGGTGGGTC |
| G97E-F | MpMS mutation | CTCAACACGCTCCGGCATGCATTCCGGCATAACC |
| G97E-R | MpMS mutation | GCCGGAATGCATGCCGGAGCGTGTTGAGGCGATGGGC |
| V99L-F | MpMS mutation | GCCCATCGCCTCCAGACGACCCGGCATGCATTCCGGC |
| V99L-R | MpMS mutation | GCCGGGTCGTCTGGAGGCGATGGGCGAGTTCATG |
| E104Y-F | MpMS mutation | CTCTCCATGAAATAGCCCATCGCCTCAACACGACC |
| E104Y-R | MpMS mutation | GTGTTGAGGCGATGGGCTATTTCATGGAGAGCATC |
| S108F-F | MpMS mutation | CATATAAAAGATAAACTCCATGAACTCGCCCATCGC |
| S108F-R | MpMS mutation | GTTCATGGAGTTTATCTTTTATATGGACAACATTG |
| I109A-F | MpMS mutation | GTCCATATAAAACGCGCTCTCCATGAACTCGCCCATCGC |
| I109A-R | MpMS mutation | CATGGAGAGCGCGTTTTATATGGACAACATTGCG |
| M112H-F | MpMS mutation | CAATGTTGTCGTGATAAAAGATGCTCTCCATGAAC |
| M112H-R | MpMS mutation | GAGCATCTTTTATCACGACAACATTGCGGAGAGCGG |
| N114D-F | MpMS mutation | CTCCGCAATGTCGTCCATATAAAAGATGCTCTC |
| N114D-R | MpMS mutation | CTTTTATATGGACGACATTGCGGAGAGCGGTAGCCAG |
| V152L-F | MpMS mutation | CTTCGCCATCAGCTGTTTCGCGCCGGTGTTGCTG |
| V152L-R | MpMS mutation | GGCGCGAAACAGCTGATGGCGAAGCTGGCGCTGCAAC |
| M153Q-F | MpMS mutation | CCAGCTTCGCTTGAACCTGTTTCGCGCCGGTGTTG |
| M153Q-R | MpMS mutation | GCGAAACAGGTTCAAGCGAAGCTGGCGCTGCAACTG |
| Q159E-F | MpMS mutation | GCTCAGCAGCTCCAGCGCCAGCTTCGCCATAAC |
| Q159E-R | MpMS mutation | GAAGCTGGCGCTGGAGCTGCTGAGCATTGACCCGG |
| A179L-F | MpMS mutation | CAAAACCCGCCAGCCACTCTTTCCACGCCTTCATA |
| A179L-R | MpMS mutation | GTGGAAAGAGTGGCTGGCGGGTTTTGCGAAGCCGCG |
| I191L-F | MpMS mutation | GTACTGCTCCAGGCTATCAAAACGACGCGGCTTC |
| I191L-R | MpMS mutation | GTCGTTTTGATAGCCTAGAGCAGTACATTGACTATC |
| Q193E-F | MpMS mutation | GTCAATGTACTCCTCGATGCTATCAAAACGACG |
| Q193E-R | MpMS mutation | GATAGCATCGAGGAGTACATTGACTATCGTCTGG |
| H233P-F | MpMS mutation | GCCTTGCCCGCCGGGTTAACAATATCGCTCACTTC |
| H233P-R | MpMS mutation | GATATTGTTAACCCGGCGGGCAAGGCGCTGAGC |
| Y240L-F | MpMS mutation | GTCGTTTTGCAGGCTCAGCGCCTTGCCCGCGTGG |
| Y240L-R | MpMS mutation | CAAGGCGCTGAGCCTGCAAAACGACTTCTTTAGC |
| Y249K-F | MpMS mutation | GTCGTGCTCTTTGTTGAAGCTAAAGAAGTCG |
| Y249K-R | MpMS mutation | CTTTAGCTTCAACAAAGAGCACGACATGTTTGTG |
| A271M-F | MpMS mutation | CTTCGGTTTCCATCAGAACAAACACCGCGTTCGC |
| A271M-R | MpMS mutation | GTGTTTGTTCTGATGGAAACCGAAGGTCTGAGCC |
| V296Y-F | MpMS mutation | GTGTTTGTTCTGATGGAAACCGAAGGTCTGAGCC |
| V296Y-R | MpMS mutation | CACGAGGACGCGTACCTGCGTCTGAAAGATGAAG |
| C314Y-F | MpMS mutation | GACCCTCCAGGTAGATACGCAGCTTGTAGCTAAC |
| C314Y-R | MpMS mutation | CAAGCTGCGTATCTACCTGGAGGGTCTGGTGGAC |
| D333P-F | MpMS mutation | CTGCTGTAACGCGGGCAGCTCGCGCTCCAAACCAG |
| D333P-R | MpMS mutation | GCGCGAGCTGCCCGCGTTACAGCAGCTATCGTCG |
| S336H-F | MpMS mutation | GACGATAGCTGTGGTAACGATCGCAGCTCGCGCTC |
| S336H-R | MpMS mutation | CTGCGATCGTTACCACAGCTATCGTCGTGAAAAGC |

[a] Modified triplet codons in mutational primers are underlined.

**ZTR_06220, KUL85185** (Talaropentaene synthase, TvTS)

MDFKYSRELKLESLDALNLTEGIPLRVNENIDLEFRGIERAHSDWERYVGKLNGFHGGRGPQFGFVSACIPECLPERMETVSYANEFAFLHDDMTDAASKDQVNGLNDDLLGGLDFTTEARSSASGKQQMQAKLLLEMLSIDRERTMVTIKAWADFMRGAAGRDHHRGFSSLDEYIPYRCADCGEKFWFGLVTFAMALSIPEQELELVQRLAQNAYLAAGLTNDLYSYEKEQLVAERSGTGQVFNAIAVIMQEHSVSISEAEDICRGRIREYAAKYVRDVADLRAKNELSRDSLAYLETGLYGISGSTAWNLDCPRYQVSTFVDFKTPEDETAKEEFIHVPEQKQFVGDGSIEDQTTEGNQEIVLRKLPQMSTEVIEAPYTYVKSLPSKGVRQRAMHAINTWLQVPMAKMKLIEDVVERIHNSSLMLDDIEDSSPLRREYPAAHMIFGVPQTINSANYELVLALNAAHQLGNPTCLQIFIEELQRLNVGQSYDLYWTHNMITPSMNDYLRMIDSKTGGLFSMLSRLMVACSPRTVSADFDSLSRLVGRFFQIRDDYQNLVSAEYSKQKGFCEDLDEGKYSLPLIHALETCVNSDRDMLRSLLVQRRVAGHLTFEQKKLVLQIMQRCESLEFTKSQLCVLQTRIQEEIDKLVAEFGDENFSLRLLVELLVVG

**MPH_02178, EKG20455** (Macrophomene synthase, MpMS)

MCNTKCYNTLAKMTVITEPAMEYMYSVPLDESEYDKCGFCQDPRYRPRRHKDQHLARAGSAKAKELCEALIGVYPRPTCESAVGHSIALVMPECMPGRVEAMGEFMESIFYMDNIAESGSQQDTGNLGTEWANDMETGPTTSVNSNTGAKQVMAKLALQLLSIDPVCAGNVMKAWKEWAAGFAKPRRFDSIEQYIDYRLVDSGAIVAVHLMNFGMGLDISVEELREVSDIVNHAGKALSYQNDFFSFNYEHDMFVKLPDSIGIANAVFVLAETEGLSLAEAKERVKELAKEHEDAVLRLKDEVESKVSYKLRICLEGLVDMVVGNLVWSASCDRYSSYRREKHQMELPIRIQGPPTPPQEPVYEKATLPNGKQLDAPTESSGKDLSDGVATLSGDEPVLGDEIVSAPIKYLESLPSKGFREAIIDGMNGWLNLPARSVSIIKDVVKHIHTASLLCDDIEDSSPLRRGQPSAHIIFGVSQTVNSTSYLWTLAIDRLSELSSPKSLRIFIDEVRKMQIGQSFDLHWTAALQCPSEEEYLSMIDMKTGGLFHLLIRLMIAESPRKVDMDFSGLVSMTGRYFQIRDDLSNLTSEEYENQKGYCEDLDEGKYSLPLIHALKHTKNKVQLESLLIQRKTQGGMTLEMKRLAIQIMKEAGSLEHTRKVVLELQDAVHRELAKLEEAFGQENYVIQLALERLRIKA

**Cgl13855, NMDCN0000R73** (Colleterpenol synthase, CgCS)

MPTFAQPVPDDIVASSGLRSKFRPHVHGNYQNCVEPSKGMETFYNDAMSTQLESKTLADIPGLGLVHPMALAMANCLPERLPAITRFADFTILNDDYYDIAKRDEIEKVNSDIQDALQDASAPGNKTKSSGGSDIDFKPKQMQAALVLELIMLDQQLAMDIMSSYSQGLDVATFAPDNLRTLDEYLPVRKVNSGLDVTAEMVCFGMGLRIAKEDKAKLRPVVDLANFAITVVNDLYSWPKEIKCHLETPGSELPFNAVAVLMRHGGYSEPEAFRILYAKQAELEAEHLRQLDALRAQEGGRLPENQELYVENAQRAVCGSELWSVYTTRYPSKADLQQPEVEFVDGSFRYVADSEGAGEEKVVYESVDSVPTEVEDEFSSSDASPGSVDQAISTPPSTTFCSCEDEDEHIANVKEVSEDEANGVRDMSDISQKSKKKIDTLLPEGPGLTTYASRLEAAPDHAVITPIKYLASLPSKGVRDTFIDALNWWLEVPEDSLRTIKTIISMLHDSSLILDDIEDDSTLRRGSPAAHMIFGTAQCINAANHIFVMVLAELQKLRSPLKTAILIEELESLFVGQADDLHWKYHVDCPSTEDYMEMIDNKTGGLFRLCVRLLQAESTRTDVLDLDPRPFVRQLSLFFQIRDDYQNLVSDAYAKQKGFAEDLDEGKISLPIILTLQRARTRPEIMGVLKHKQPGPMALEMKQYIVKEMEKCGALESTRELLQGMQEDLIAELRRLEGDFGAKNATLELVLRRLWIS

**Supplementary Figure 1 │ Amino acid sequences of TvTS, MpMS and CgCS.** Conserved motifs are highlighted in yellow, and the PT domains are underlined.


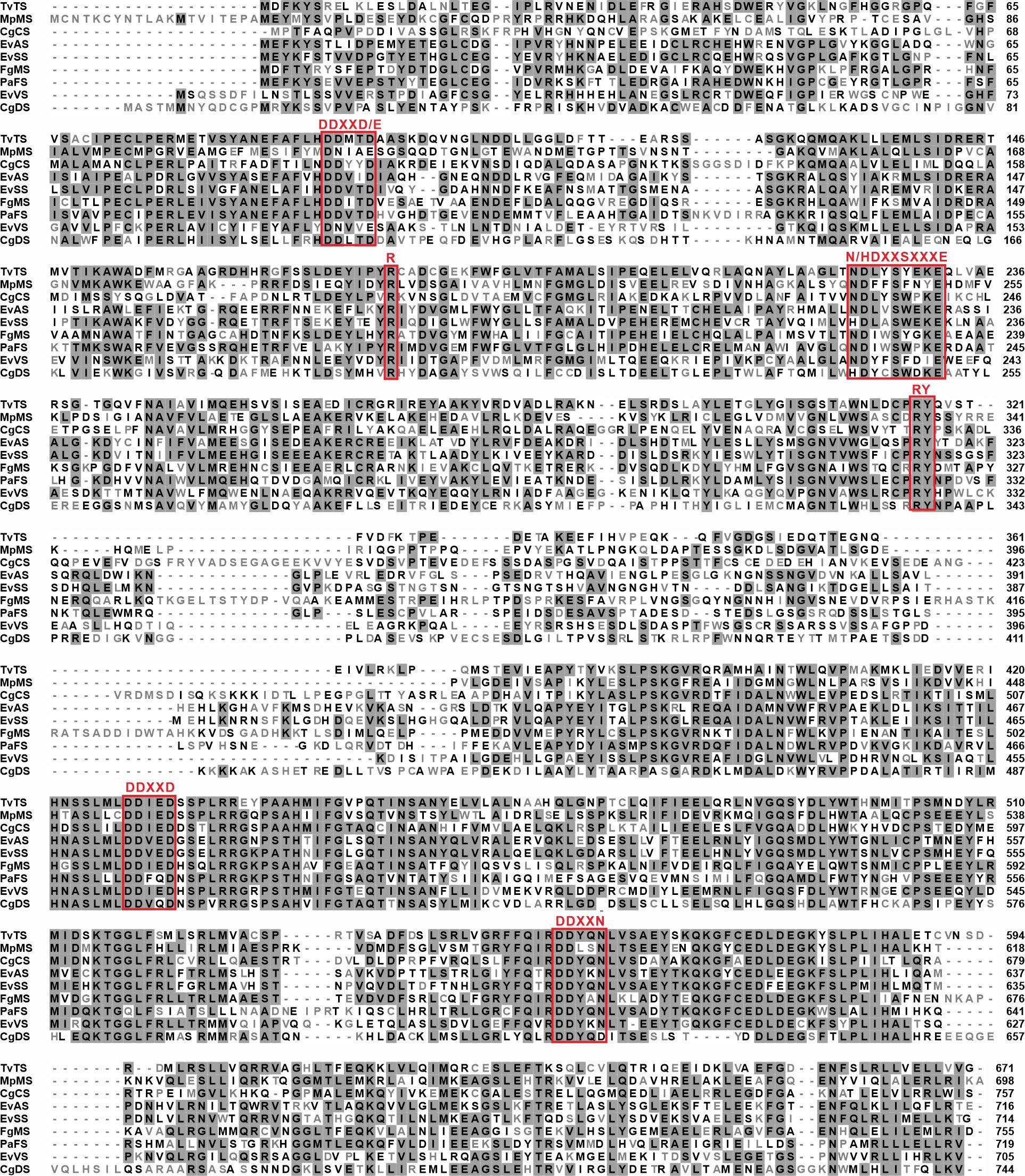


**Supplementary Figure 2 │ Amino acid alignment of TvTS, MpMS and CgCS with characterized chimeric terpene synthases from filamentous fungi.** Protein alignments were done with CLUSTAL W. The boxed sequences indicate the highly conserved “DDXXD/E”, “NSE/DTE”, “R”, and “RY” motifs of the TC domain, and the “DDXXD” and “DDXXN” motifs of the PT domain.


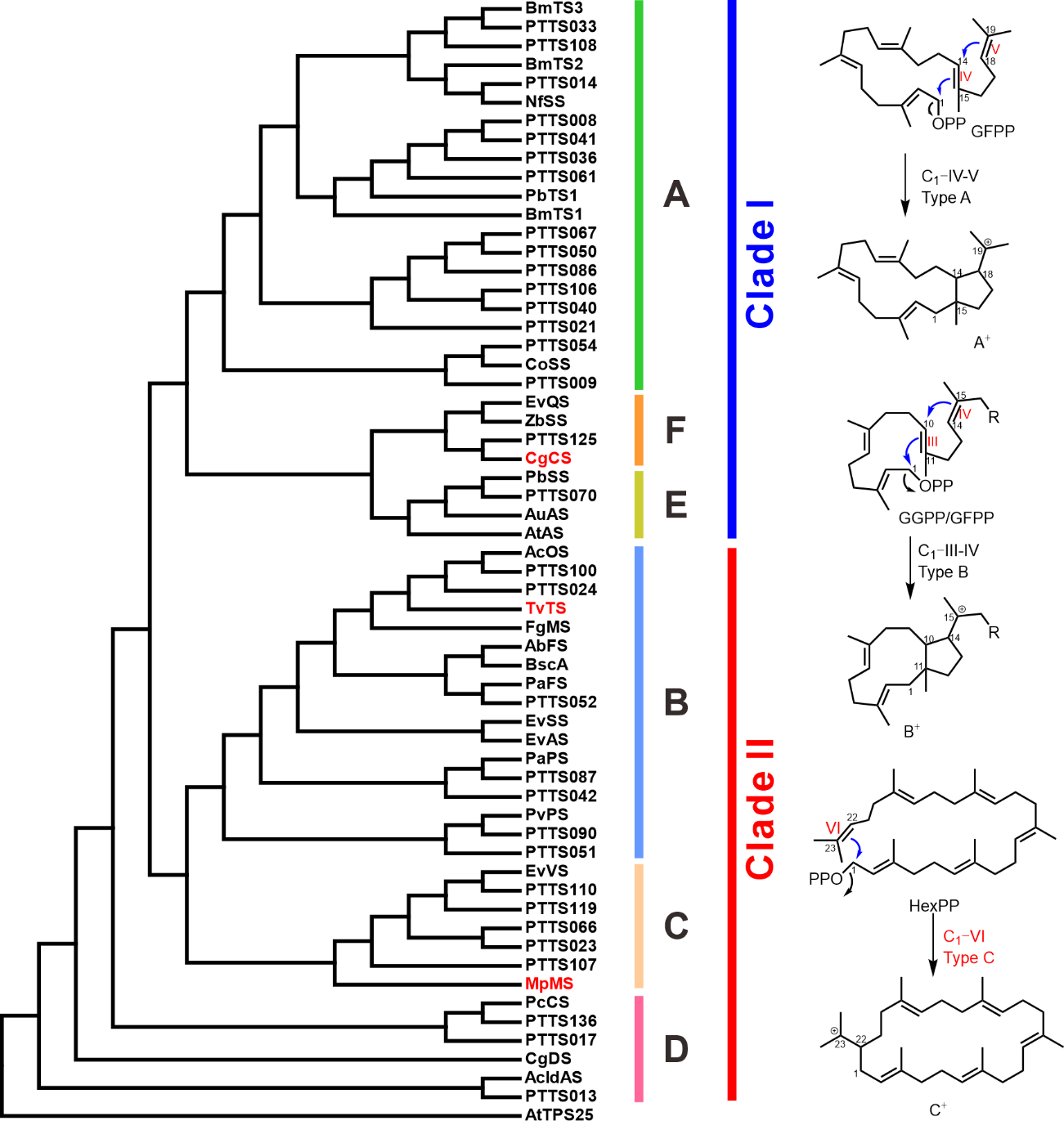


**Supplementary Figure 3 │ Phylogenetic analysis of TvTS, MpMS and CgCS with characterized fungal chimeric terpene synthases.** Phylogenetic tree constructed from 58 chimeric terpene synthases that group into two clades with six subclades. Clades I enzymes (with subclades A, E, and F) show initial type A (C1-IV-V) cyclization, clade II enzymes (with subclades B, C, and D) show initial type B (C1-III-IV) cyclization. Type C represent an unprecedented (C1-VI) cyclization mode that cyclization occurs between C1-cation and C22–C23 olefin (VI) of HexPP. Subclades exhibit different stereochemical courses for the initial cyclization step. Outgroup (bottom): AtTPS25 (*Arabidopsis thaliana*)^17^.

**
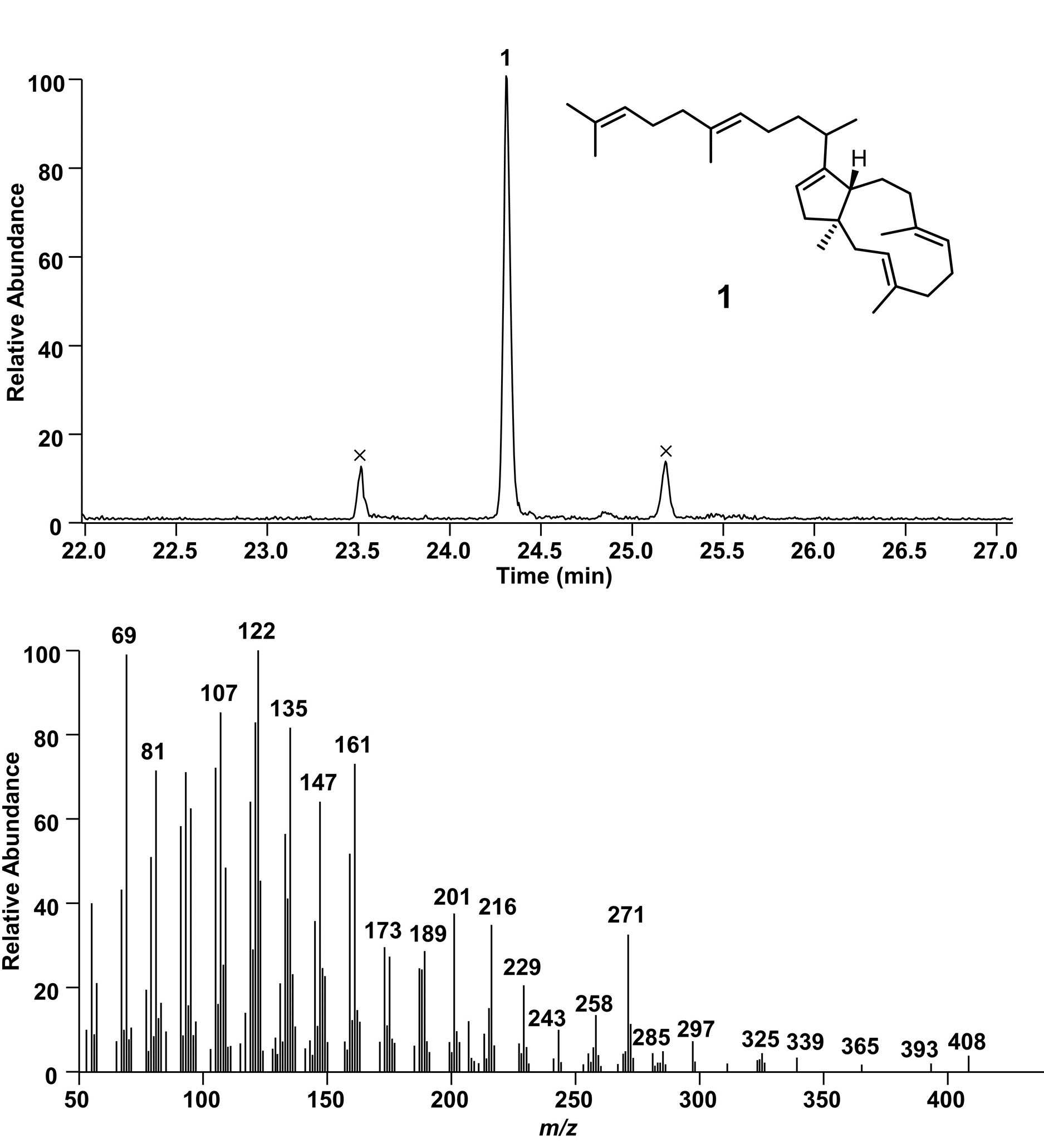
**

**Supplementary Figure 4 │ Production of 1 by *S. cerevisiae* XM139.** Total ion chromatogram of an extract obtained from a culture of *S. cerevisiae* XM139.

**Supplementary Figure 5 │ Structure elucidation of 1.** Carbon numbering follows HexPP numbering to indicate the biosynthetic origin of each carbon by same number. Bold: ^1^H-^1^H COSY correlations, single headed arrows: key HMBC correlations, double headed arrows: key NOESY correlations.

**Supplementary Table 3 │ NMR data of 1 recorded in C_6_D_6_ (700 MHz, 298 K) for the labeling experiments.**

| C^[a]^ | type | ^13^C^[b]^ | ^1^H^[b]^ |
| --- | --- | --- | --- |
| 1 | CH_2_ | 41.89 | 2.40 (dd, ^2^*J*_H,H_ = 12.3 Hz, ^3^*J*_H,H_ = 12.3 Hz, 1H, H_β_)  1.71 (m, 1H, H_α_) |
| 2 | CH | 125.82 | 5.22 (m, 1H) |
| 3 | C_q_ | 134.30 | – |
| 4 | CH_2_ | 40.43 | 2.13 (m, 1H, H_β_)  2.04 (m, 1H, H_α_) |
| 5 | CH_2_ | 24.88 | 2.27 (m, 1H, H_β_)  1.99 (m, 1H, H_α_) |
| 6 | CH | 129.27 | 4.88 (m, 1H) |
| 7 | C_q_ | 133.05 | – |
| 8 | CH_2_ | 38.61 | 2.28 (m, 1H, H_α_)  2.14 (m, 1H, H_β_) |
| 9 | CH_2_ | 26.43 | 1.58 (m, 1H, H_β_)  1.39 (m, 1H, H_α_) |
| 10 | CH | 48.08 | 2.17 (m, 1H) |
| 11 | C_q_ | 46.19 | – |
| 12 | CH_2_ | 48.99 | 2.31 (m, 1H, H_α_)  2.02 (m, 1H, H_β_) |
| 13 | CH | 119.83 | 5.29 (m, 1H) |
| 14 | C_q_ | 153.43 | – |
| 15 | CH | 32.37 | 2.13 (m, 1H) |
| 16 | CH_2_ | 37.40 | 1.70 (m, 1H)  1.31 (m, 1H) |
| 17 | CH_2_ | 26.10 | 2.13 (m, 2H) |
| 18 | CH | 125.03 | 5.31 (m, 1H) |
| 19 | C_q_ | 135.06 | – |
| 20 | CH_2_ | 40.33 | 2.12 (m, 2H) |
| 21 | CH_2_ | 27.29 | 2.21 (m, 2H) |
| 22 | CH | 124.92 | 5.25 (m, 1H) |
| 23 | C_q_ | 131.23 | – |
| 24 | CH_3_ | 25.91 | 1.68 (d, ^4^*J*_H,H_ = 1.4 Hz, 3H) |
| 25 | CH_3_ | 17.79 | 1.58 (br s, 3H) |
| 26 | CH_3_ | 16.17 | 1.64 (br s, 3H) |
| 27 | CH_3_ | 19.75 | 1.16 (d, ^3^*J*_H,H_ = 6.9 Hz, 3H) |
| 28 | CH_3_ | 22.80 | 1.15 (s, 3H) |
| 29 | CH_3_ | 15.42 | 1.55 (br s, 3H) |
| 30 | CH_3_ | 16.38 | 1.55 (br s, 3H) |

[a] Carbon numbering follows numbering of HexPP and is shown in Supplementary Figure 5. [b] Chemical shifts *δ* in ppm, coupling constants *J* in Hertz, s = singlet, d = doublet, m = multiplet, br = broad. Diastereotopic hydrogens were assigned by the labeling experiments (Supplementary Figures 16 and 17).

**Supplementary Table 4 │ NMR data of 1 recorded in CCl_3_ (600 MHz, 298 K) for structure elucidation.**

| C^[a]^ | type | ^13^C^[b]^ | ^1^H^[b]^ |
| --- | --- | --- | --- |
| 1 | CH_2_ | 41.47 | 2.34 (t, *J* = 12.3, 1H)  1.66 (m, 1H) |
| 2 | CH | 125.40 | 5.17 (m, 1H) |
| 3 | C_q_ | 134.64 | - |
| 4 | CH_2_ | 40.01 | 2.08 (m, 1H)  1.98 (m, 1H) |
| 5 | CH_2_ | 24.47 | 2.21 (m, 1H)  1.94 (m, 1H) |
| 6 | CH | 128.86 | 4.83 (m, 1H) |
| 7 | C_q_ | 133.63 | - |
| 8 | CH_2_ | 38.20 | 2.23 (m, 1H)  2.09 (m, 1H) |
| 9 | CH_2_ | 26.02 | 1.53 (m, 1H)  1.33 (m, 1H) |
| 10 | CH | 47.67 | 2.11 (m, 1H) |
| 11 | C_q_ | 45.78 | - |
| 12 | CH_2_ | 48.58 | 2.25 (m, 1H)  1.97 (m, 1H) |
| 13 | CH | 119.41 | 5.24 (m, 1H) |
| 14 | C_q_ | 153.02 | - |
| 15 | CH | 31.95 | 2.07 (m, 1H) |
| 16 | CH_2_ | 36.99 | 1.65 (m, 1H)  1.26 (m, 1H) |
| 17 | CH_2_ | 25.69 | 1.06 (m, 2H) |
| 18 | CH | 124.51 | 5.21 (m, 1H) |
| 19 | C_q_ | 134.61 | - |
| 20 | CH_2_ | 39.91 | 2.08 (m, 1H)  1.98 (m, 1H) |
| 21 | CH_2_ | 26.88 | 2.15 (m, 2H) |
| 22 | CH | 124.61 | 5.26 (m, 1H) |
| 23 | C_q_ | 130.82 | - |
| 24 | CH_3_ | 25.49 | 1.64 (d, *J* = 1.46, 3H) |
| 25 | CH_3_ | 17.38 | 1.54 (s, 3H) |
| 26 | CH_3_ | 15.76 | 1.59 (s, 3H) |
| 27 | CH_3_ | 19.34 | 1.11 (br s, 3H) |
| 28 | CH_3_ | 22.39 | 1.10 (br s, 3H) |
| 29 | CH_3_ | 15.01 | 1.51 (br s, 3H) |
| 30 | CH_3_ | 15.97 | 1.50 (br s, 3H) |

[a] Carbon numbering as shown in Fig. 2e of main text. [b] Chemical shifts *δ* in ppm, coupling constants *J* in Hertz, s = singlet, d = doublet, m = multiplet, br = broad.


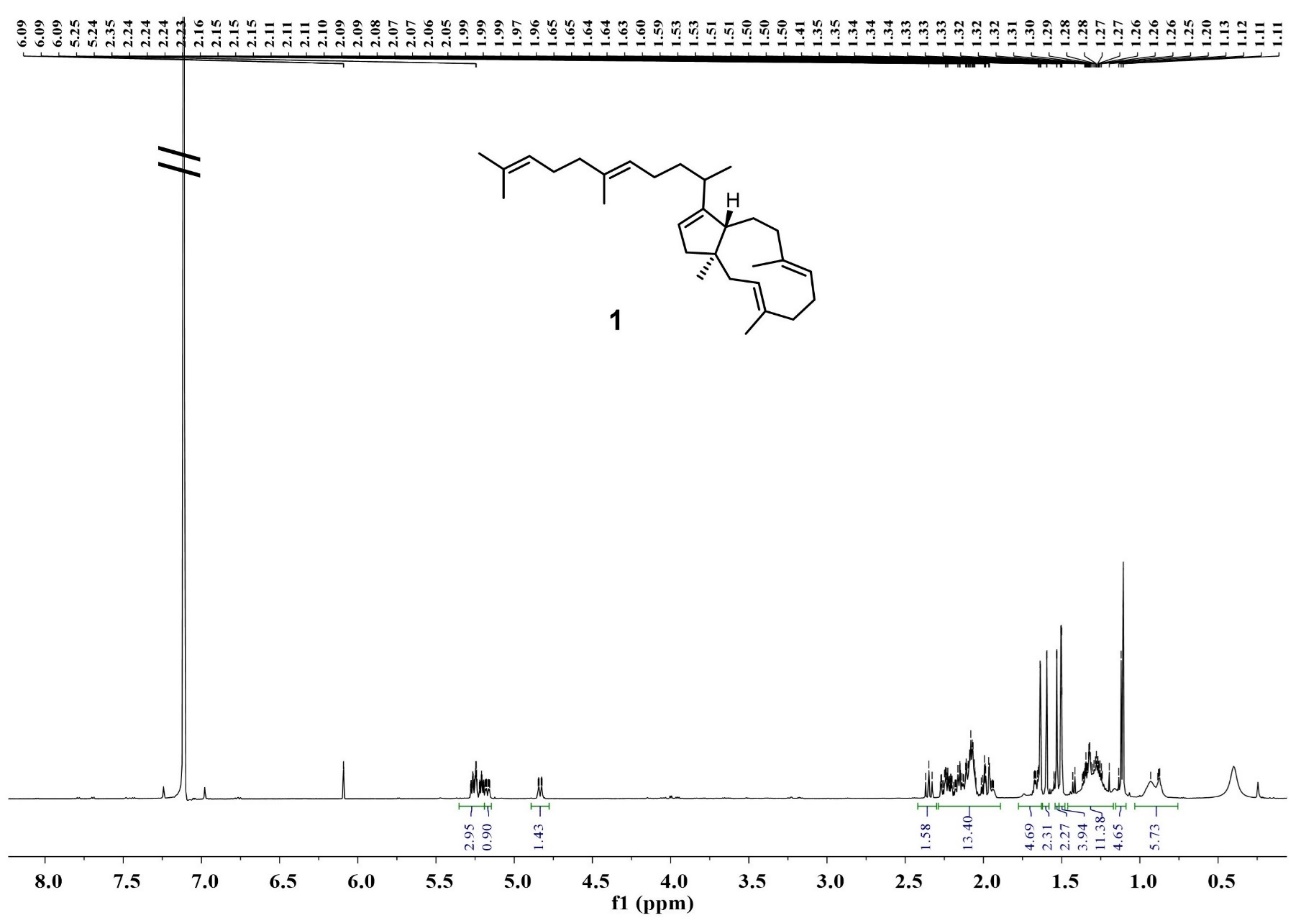


**Supplementary Figure 6 │ ^1^H-NMR spectrum of 1 (C_6_D_6_, 600 MHz).**


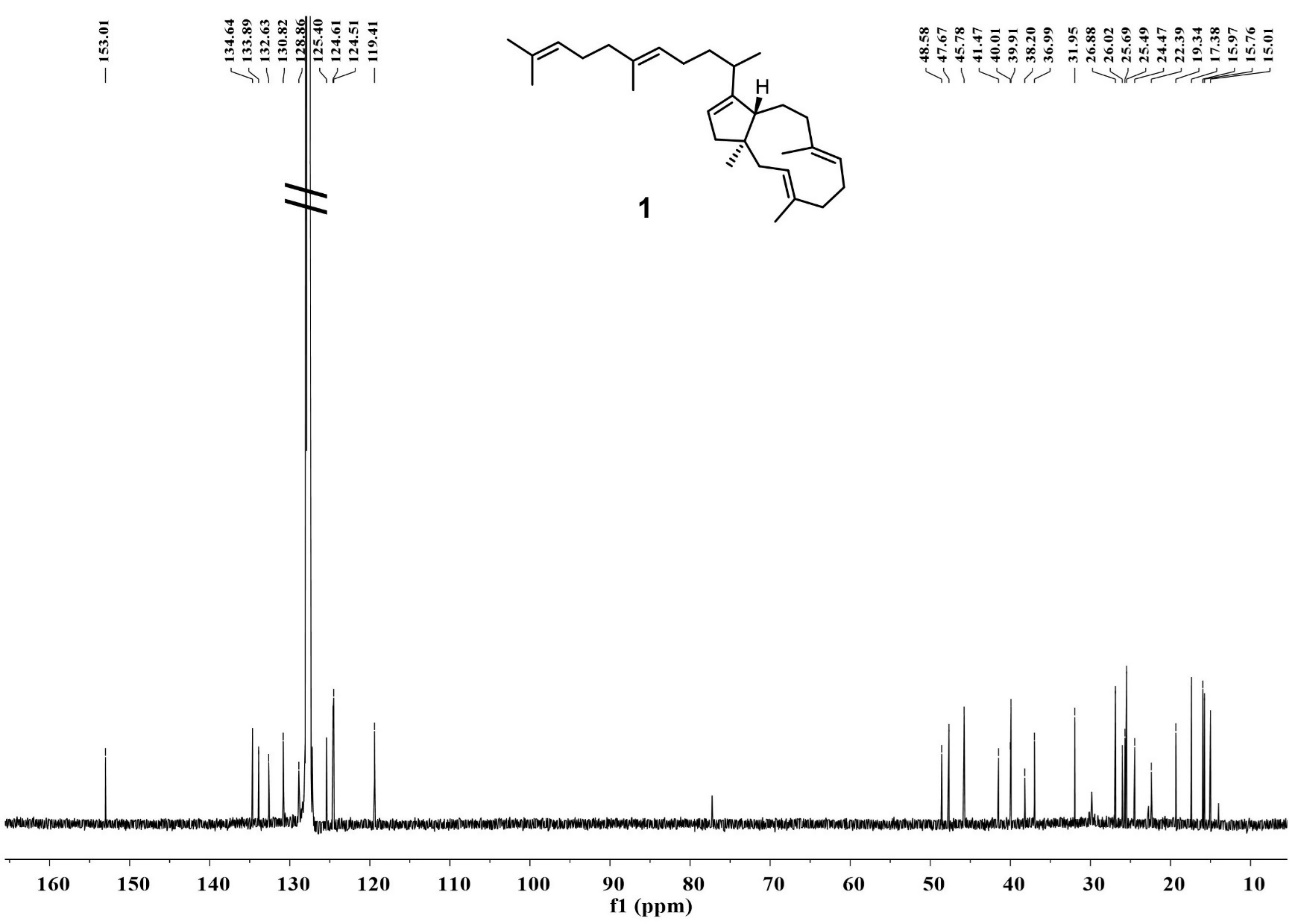


**Supplementary Figure 7 │ ^13^C-NMR spectrum of 1 (C_6_D_6_, 150 MHz).**


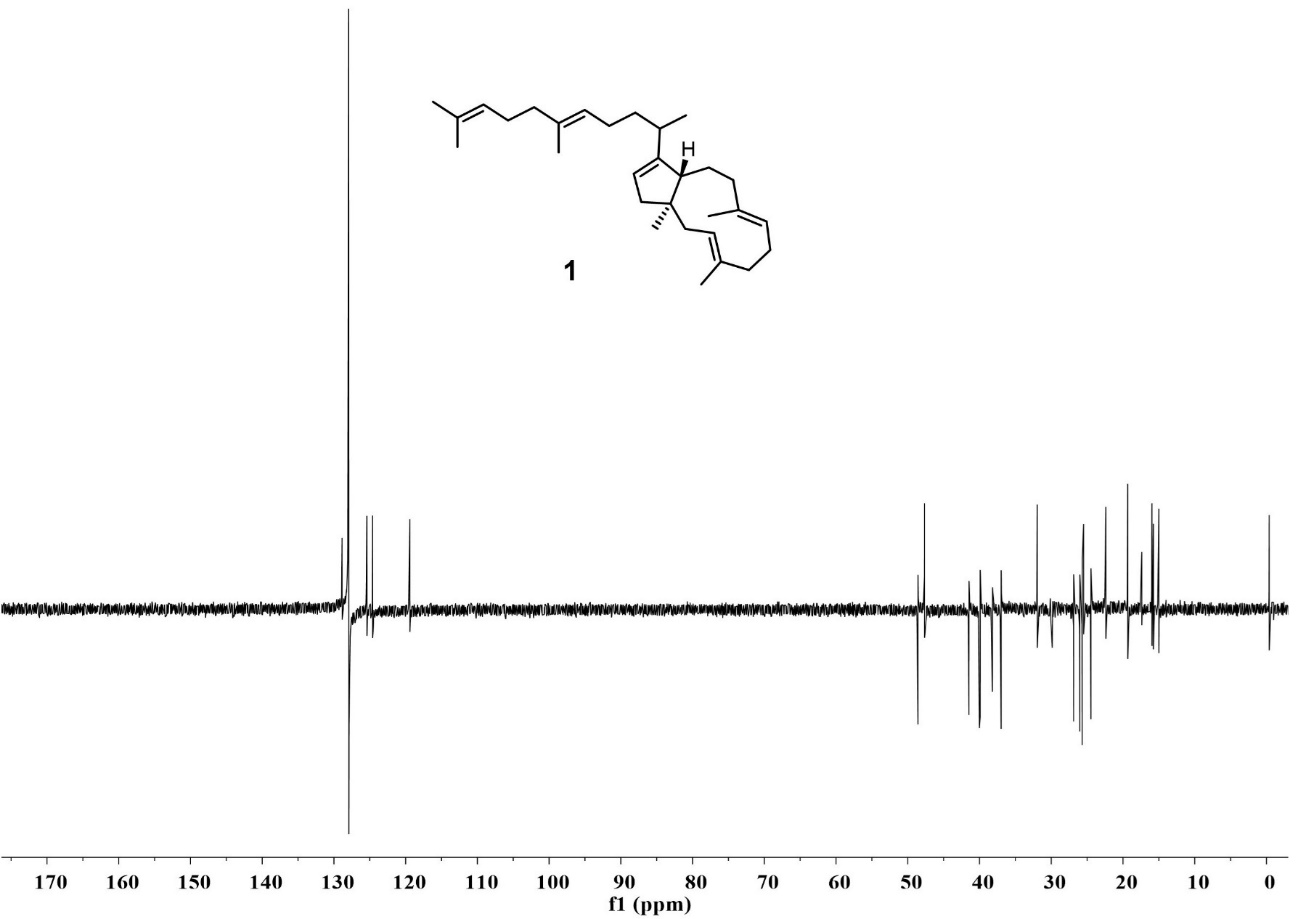


**Supplementary Figure 8 │ ^13^C-DEPT spectrum of 1 (C_6_D_6_, 150 MHz).**


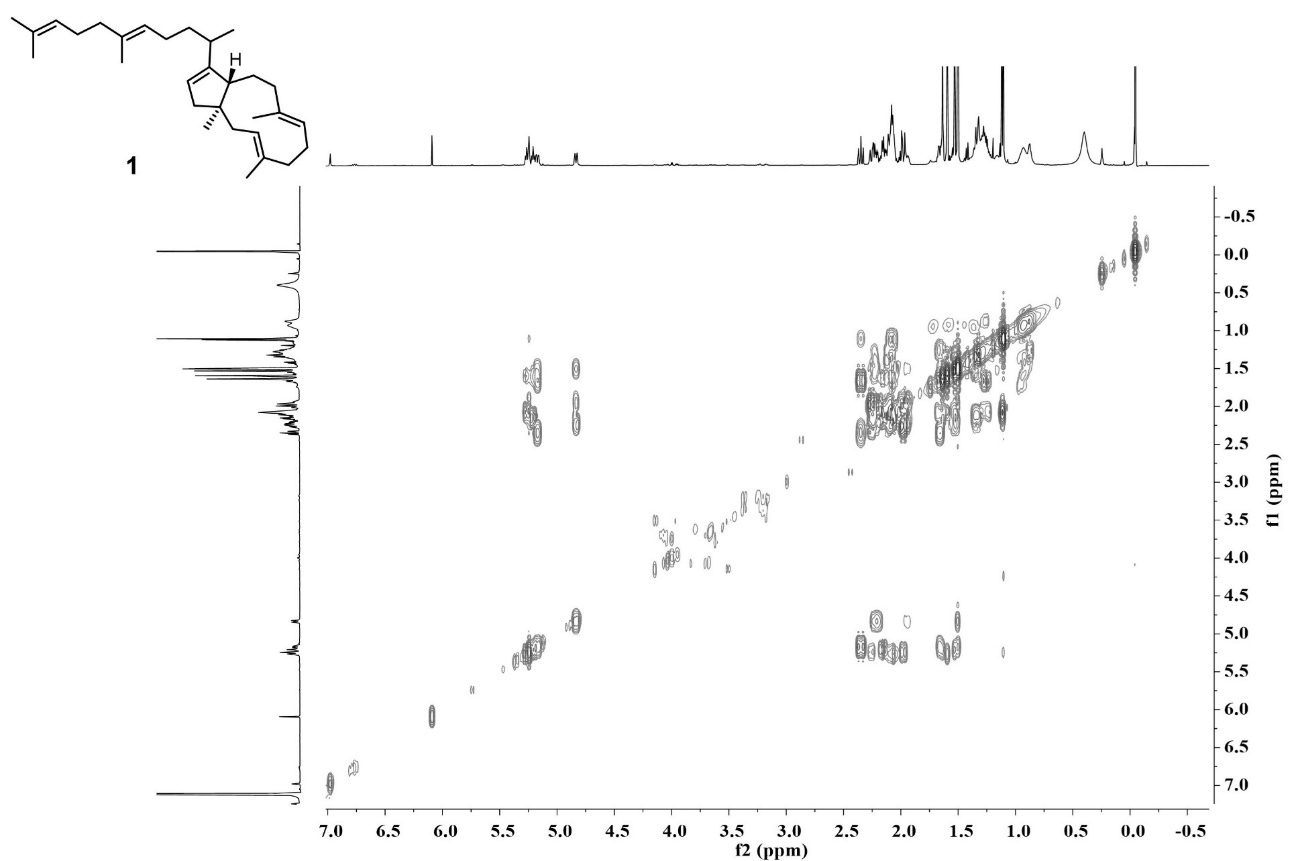


**Supplementary Figure 9 │ ^1^H-^1^H-COSY spectrum of 1 (C_6_D_6_).**


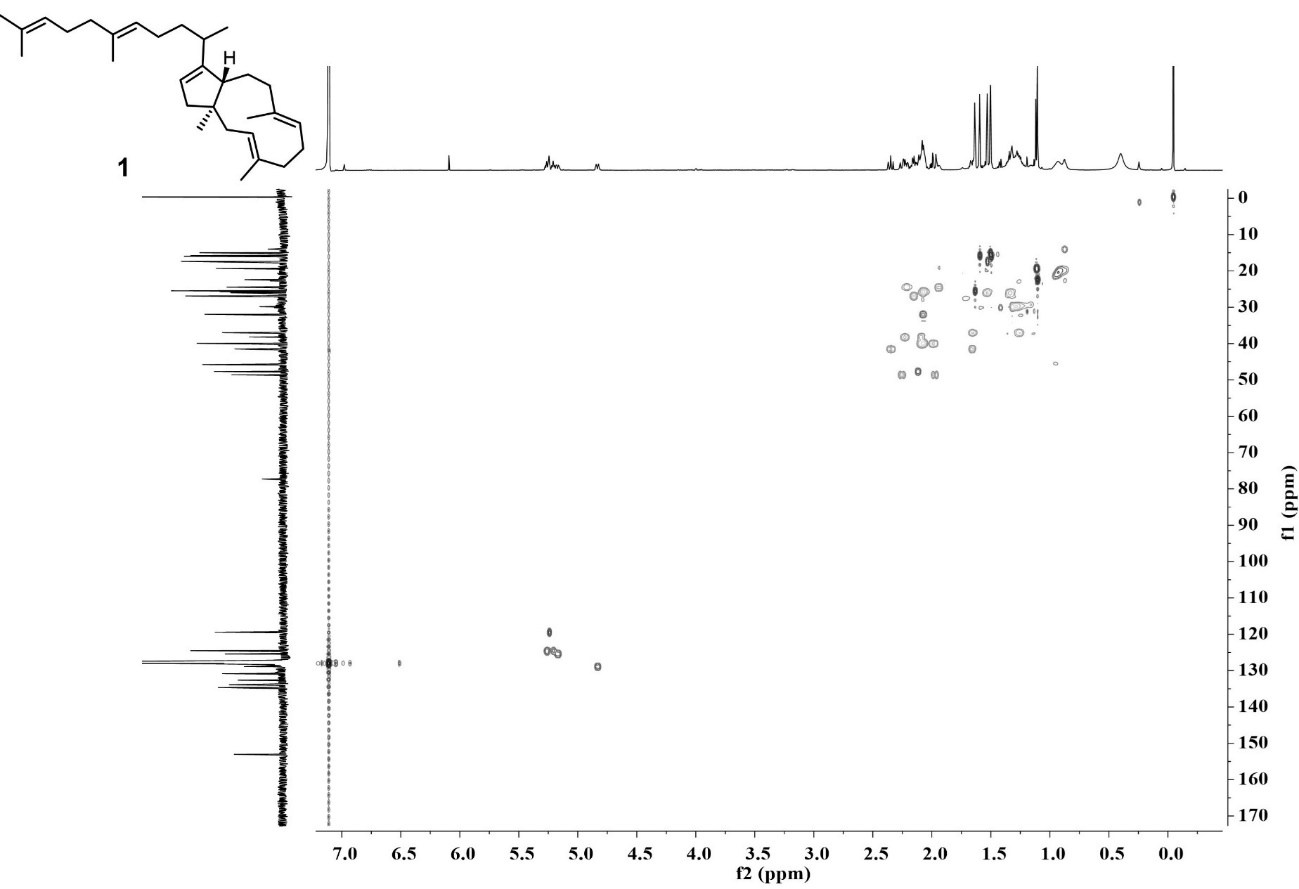


**Supplementary Figure 10 │ HSQC spectrum of 1 (C_6_D_6_).**


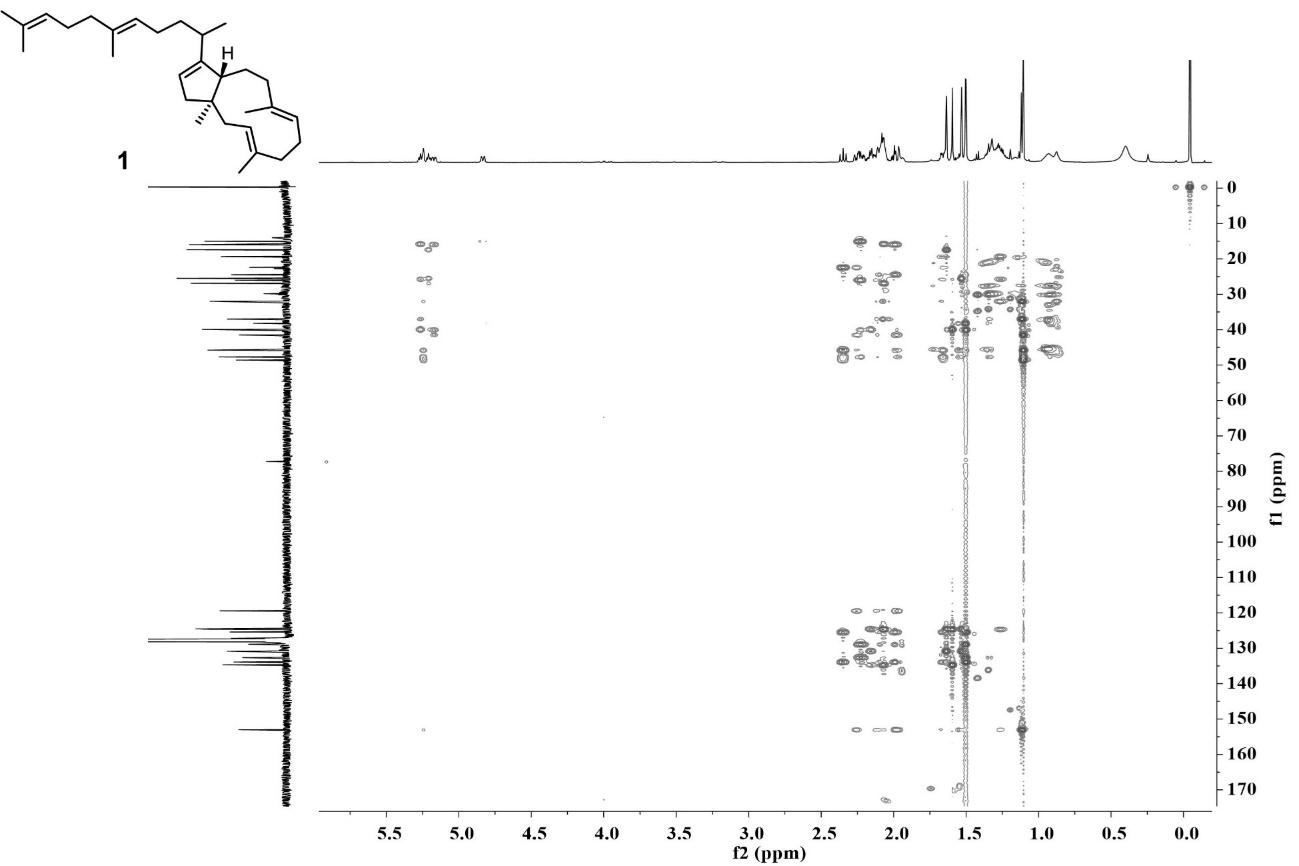


**Supplementary Figure 11 │ HMBC spectrum of 1 (C_6_D_6_).**


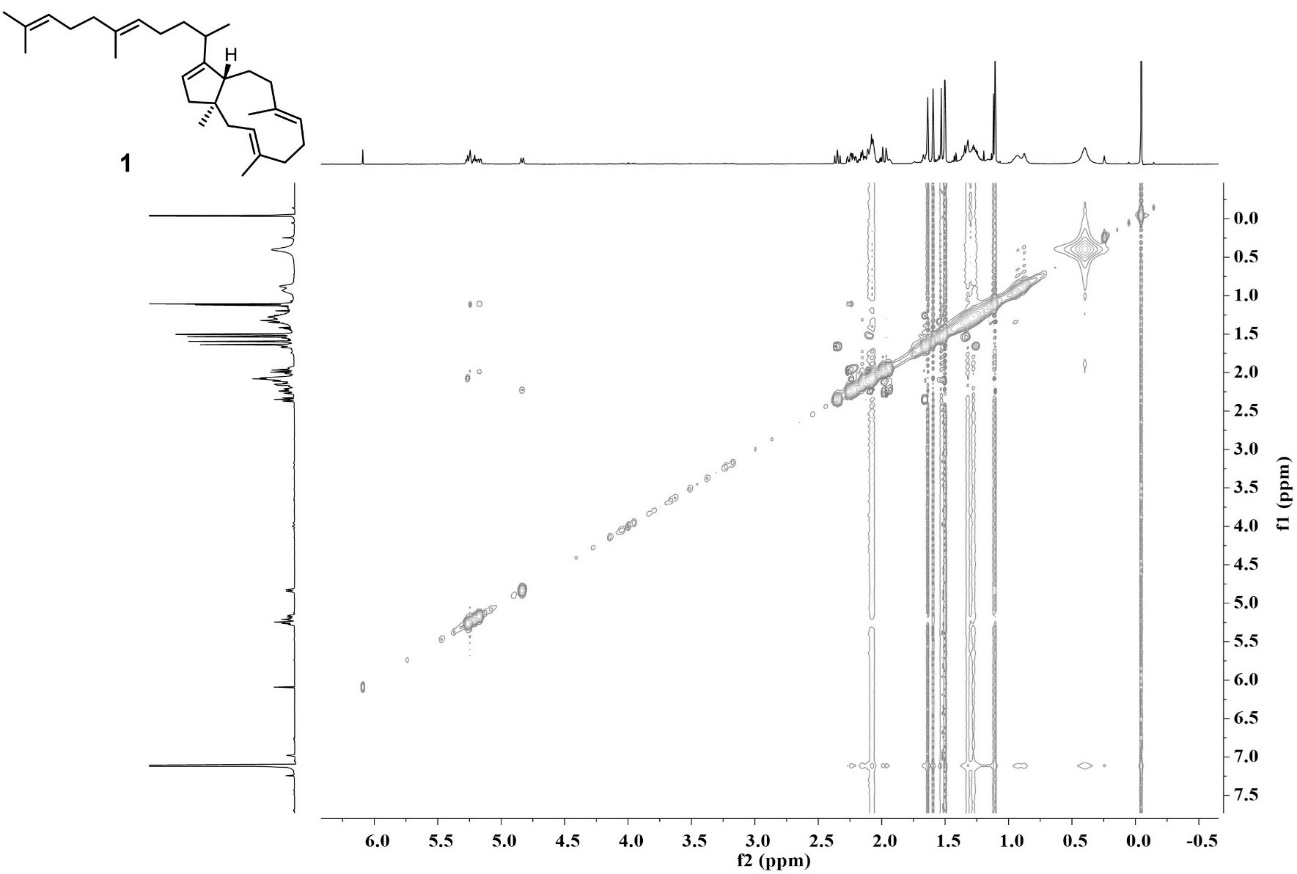


**Supplementary Figure 12 │ NOESY spectrum of 1 (C_6_D_6_).**

**Supplementary Table 5 │ Isotopic labeling experiments performed in this study.**

| **Substrate(s)** | **Enzyme(s)** | **Results shown in** |
| --- | --- | --- |
| (*E*)-(4-^13^C,4-^2^H)IPP^18^ + GPP | TvTS-PT + TvTS-TC | Supplementary Figure 16 |
| (*Z*)-(4-^13^C,4-^2^H)IPP^18^ + GPP | TvTS-PT + TvTS-TC | Supplementary Figure 16 |
| (*R*)-(1-^13^C,1-^2^H)IPP^19^ + GPP | TvTS-PT + TvTS-TC | Supplementary Figures 17 and 19 |
| (*S*)-(1-^13^C,1-^2^H)IPP^19^ + GPP | TvTS-PT + TvTS-TC | Supplementary Figures 17 and 19 |
| (3-^13^C,2-^2^H)FPP^20^ + IPP | TvTS-PT + TvTS-TC | Supplementary Figure 18 |
| (1-^13^C)IPP^21^ + GFPP | MpMS | Supplementary Figure 32 |
| (1-^13^C)GGPP^22^ + IPP | MpMS | Supplementary Figure 32 |
| (1-^13^C)FPP^23^ + IPP | MpMS | Supplementary Figure 32 |
| (5-^13^C)FPP^23^ + IPP | MpMS | Supplementary Figure 32 |
| (2-^13^C)GFPP^24^ + IPP | MpMS | Supplementary Figure 32 |
| (2-^13^C)GGPP^21^ + IPP | MpMS | Supplementary Figure 32 |
| (2-^13^C)FPP^23^ + IPP | MpMS | Supplementary Figure 32 |
| (6-^13^C)FPP^23^ + IPP | MpMS | Supplementary Figure 32 |
| (3-^13^C)IPP^21^ + GGPP | MpMS | Supplementary Figure 32 |
| (3-^13^C)GGPP^22^ + IPP | MpMS | Supplementary Figure 32 |
| (3-^13^C)FPP^23^ + IPP | MpMS | Supplementary Figure 32 |
| (7-^13^C)FPP^23^ + IPP | MpMS | Supplementary Figure 32 |
| (4-^13^C)IPP^21^ + GFPP | MpMS | Supplementary Figure 32 |
| (4-^13^C)IPP^21^ + GGPP | MpMS | Supplementary Figure 32 |
| (4-^13^C)GGPP^22^ + IPP | MpMS | Supplementary Figure 32 |
| (4-^13^C)FPP^23^ + IPP | MpMS | Supplementary Figure 32 |
| (8-^13^C)FPP^23^ + IPP | MpMS | Supplementary Figure 32 |
| (14-^13^C)FPP^23^ + IPP | MpMS | Supplementary Figure 32 |
| (15-^13^C)FPP^23^ + IPP | MpMS | Supplementary Figure 32 |
| (20-^13^C)GGPP^21^ + IPP | MpMS | Supplementary Figure 32 |
| (25-^13^C)GFPP^24^ + IPP | MpMS | Supplementary Figure 32 |
| (9-^13^C)GPP^25^ + IPP | MpMS | Supplementary Figure 33 |
| (12-^13^C)FPP^23^ + IPP | MpMS | Supplementary Figure 33 |
| (*R*)-(1-^2^H)IPP^18^ + GFPP | MpMS | Supplementary Figure 34 |
| (*S*)-(1-^2^H)IPP^18^ + GFPP | MpMS | Supplementary Figure 34 |
| (*R*)-(1-^13^C,1-^2^H)IPP^19^ + DMAPP | MpMS + IDI | Supplementary Figure 35 |
| (*S*)-(1-^13^C,1-^2^H)IPP^19^ + DMAPP | MpMS + IDI | Supplementary Figure 35 |


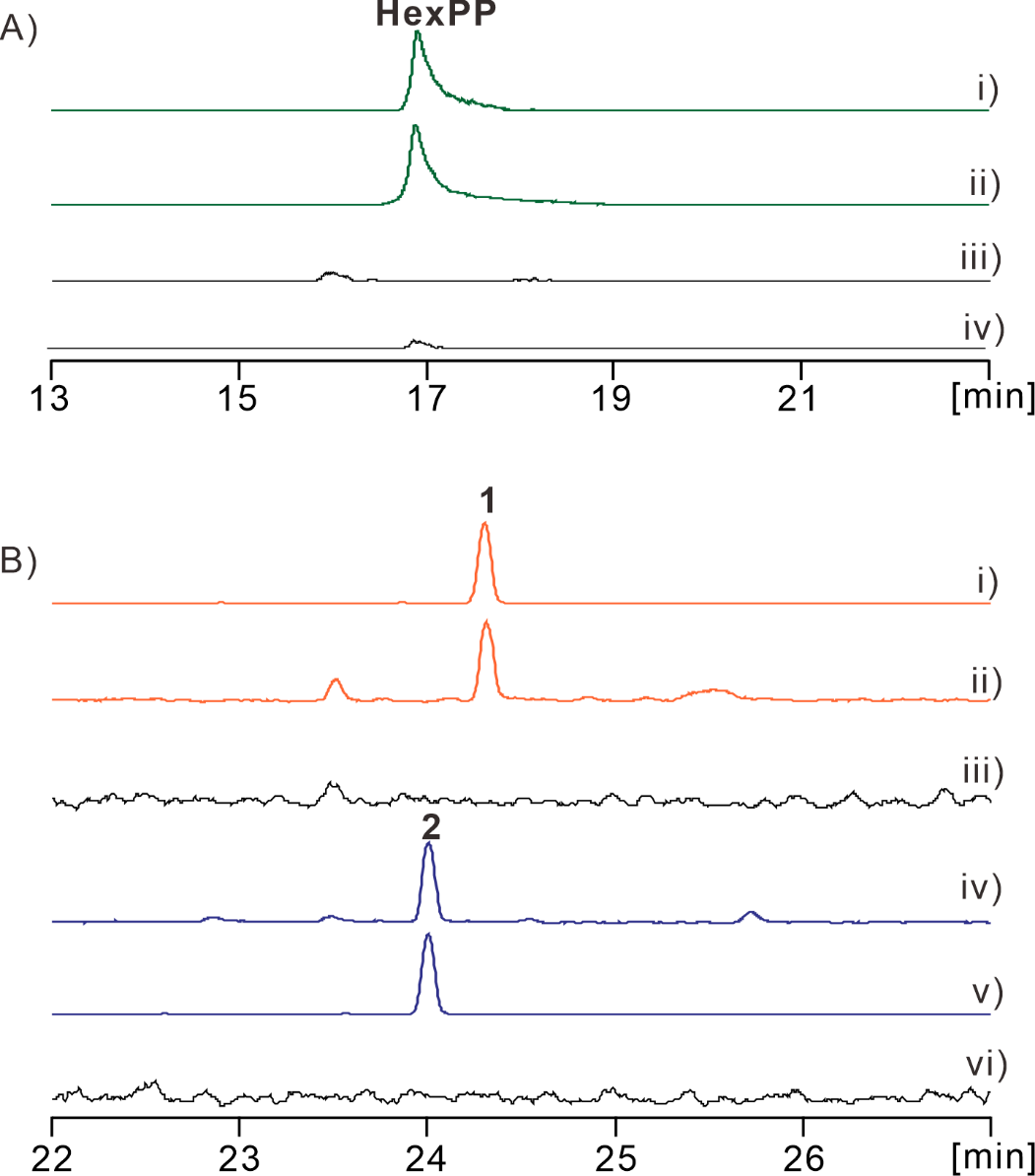


**Supplementary** **Figure 13 │ Functional characterization of TvTS and MpMS in vitro and in vivo.** A) HR-ESI-MS ion chromatograms (m/z 505.3452) of extracts obtained from incubations of DMAPP and IPP with i) TvTS-PT, ii) MpMS-PT(D114A-N115A), iii) boiled TvTS-PT, iv) boiled MpMS-PT(D114A-N115A). B) GC-EI-MS ion chromatograms of extracts from i) an incubation of DMAPP and IPP with recombinant TvTS-PT and TvTS-TC, ii) *S. cerevisiae* XM139 under heterologous expression of the gene for TvTS, iii) an incubation of DMAPP and IPP with boiled TvTS-PT and TvTS-TC, iv) an incubation of DMAPP and IPP with recombinant MpMS, v) *S. cerevisiae* XM018 under heterologous expression of the gene for MpMS, vi) iii) an incubation of DMAPP and IPP with boiled MpMS.

**
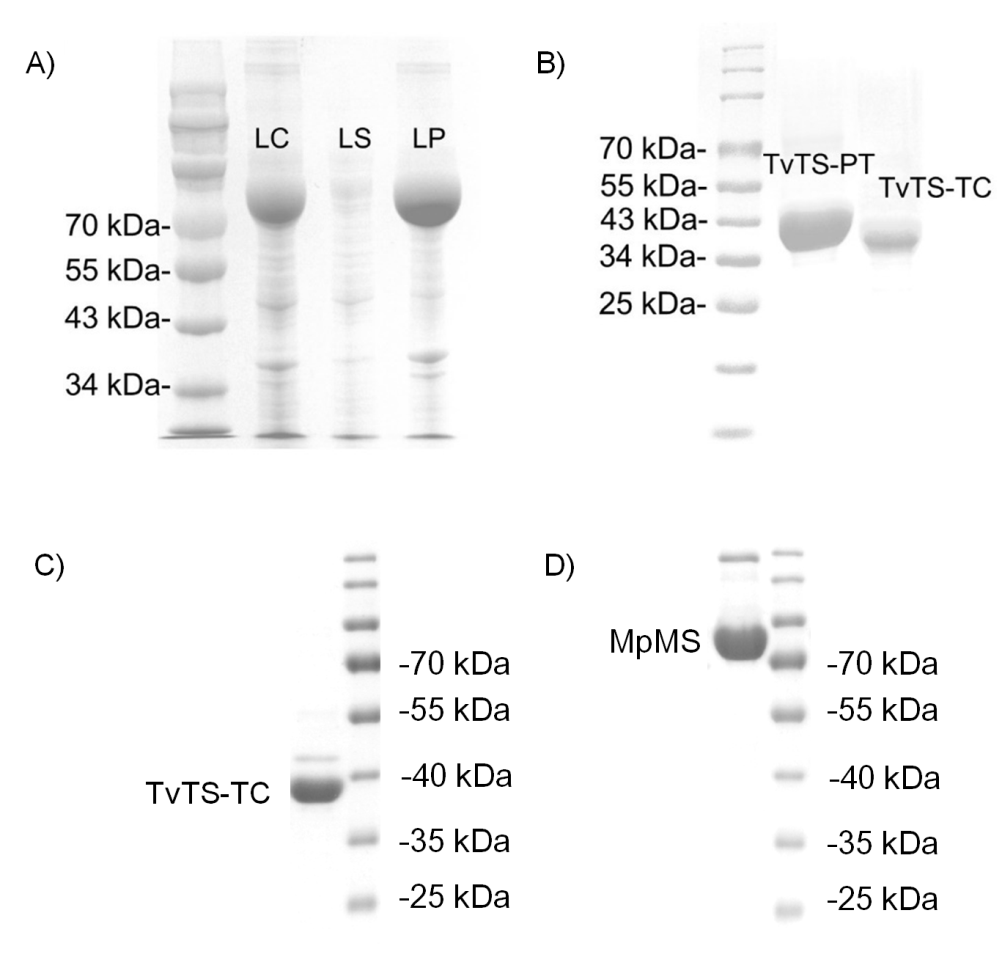
**

**Supplementary Figure 14 │ SDS-PAGE analysis of purified recombinant TvTS.** A) Heterologous expression of the codon optimized sequence of full length TvTS in *E. coli* yielded only insoluble inclusion bodies. LC: lysed cell; LS: lysed supernatant; LP: lysed precipitate. B) Spliced TvTS-PT and TvTS-TC gave soluble proteins. C) TvTS-TC purified for crystallization. D) MpMS purified for cryo-EM analysis. Each protein was purified at least three times throughout the course of the work to a similar result.


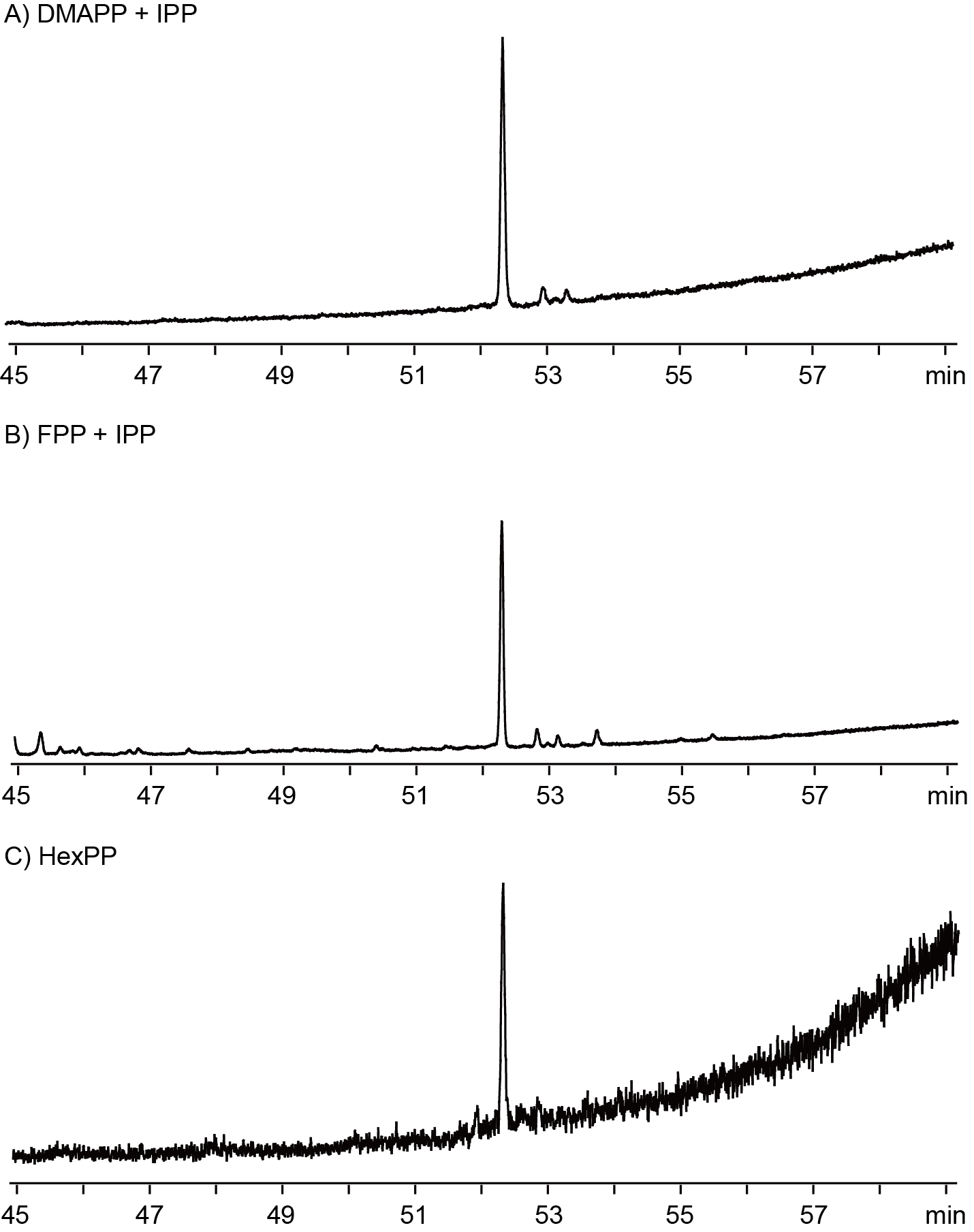


**Supplementary Figure 15 │ Substrate scope of TvTS.** Total ion chromatograms of extracts from incubations of TvTS-PT and TvTS-TC with A) DMAPP and IPP, and B) FPP and IPP, and C) incubation of HexPP with TvTS-TC.

**Supplementary Scheme 1 │ Synthesis of HexPP.** Reaction conditions: a) pyridine, acetyl chloride, CH_2_Cl_2_, 0 °C, 45 min, 98%; b) SeO_2_, *t*-BuOOH, CH_2_Cl_2_, room temperature, 24 h; NaBH_4_, MeOH/THF (1/4), 0 °C, 1 h, 26%; c) PBr_3_, Et_2_O, 0 °C, 45 min; d) PBr_3_, Et_2_O, 0 °C, 45 min; e) sodium benzenesulfinate, DMF, room temperature, overnight, 80% over 2 steps; f) *n*-BuLi, THF/HMPA (4:1), –78 °C to room temperature, overnight; g) NaOH, MeOH, room temperature, 4 h, 70 % over 3 steps; h) Pd(dppp)Cl_2_, LiEt_3_BH, THF, 0 °C, 0.5 h, 75%; i) PBr_3_, Et_2_O, 0 °C, 45 min; j) (NBu_4_)_3_HP_2_O_7_, acetonitrile, room temperature, overnight, 68%.

**Synthetic procedures**

**Synthesis of (2*E*,6*E*)-3,7,11-trimethyldodeca-2,6,10-trien-1-yl acetate (S1)**

To a CH_2_Cl_2_ (200 mL, 0 °C) solution of (2*E*,6*E*)-farnesol (4.45 g, 20.0 mmol) and pyridine (2.21 g, 28.0 mmol, 1.4 eq) was added acetyl chloride (1.88 g, 24.0 mmol, 1.2 eq) dropwise. The reaction was stirred at 0 °C for 45 min, then quenched by addition of H_2_O (200 mL). The organic layer was separated and the aqueous layer was extracted with Et_2_O (2 x 60 mL). The combined organic layers were dried with MgSO_4_ and concentrated under reduced pressure. The product **S1** (5.18 g, 19.6 mmol, 98%) was obtained via silica gel chromatography (cyclohexane/ethyl acetate, 10:1, *R*_f_ = 0.47) as a colorless oil. EI-MS (70 eV): *m*/*z* (%) = 264 (1), 204 (2), 189 (4), 175 (1), 161 (5), 147 (3), 136 (17), 121 (12), 107 (16), 93 (33), 81 (29), 69 (100), 53 (13), 43 (54). GC (HP5-MS): *I* = 1843. ^1^H NMR (500 MHz, C_6_D_6_): *δ* = 5.43 (tq, ^3^*J*_H,H_ = 7.1 Hz, ^4^*J*_H,H_ = 1.3 Hz, 1H), 5.22 (ddq, ^3^*J*_H,H_ = 8.5 Hz, ^3^*J*_H,H_ = 5.8 Hz, ^4^*J*_H,H_ = 1.5 Hz, 1H), 5.17 (tq, ^3^*J*_H,H_ = 7.1 Hz, ^4^*J*_H,H_ = 1.3 Hz, 1H), 4.61 (dd, ^3^*J*_H,H_ = 7.2 Hz, ^4^*J*_H,H_ = 1.0 Hz, 2H), 2.18 – 2.13 (m, 2H), 2.11 – 2.04 (m, 4H), 1.99 – 1.93 (m, 2H), 1.70 (s, 3H), 1.68 (q, ^4^*J*_H,H_ = 1.3 Hz, 3H), 1.56 (d, ^4^*J*_H,H_ = 1.3 Hz, 3H), 1.54 (d, ^4^*J*_H,H_ = 1.2 Hz, 3H), 1.51 (d, ^4^*J*_H,H_ = 1.5 Hz, 3H) ppm. ^13^C NMR (126 MHz, C_6_D_6_): *δ* = 170.09 (C_q_), 141.60 (C_q_), 135.44 (C_q_), 131.21 (C_q_), 124.90 (CH), 124.25 (CH), 119.50 (CH), 61.21 (CH_2_), 40.19 (CH_2_), 39.83 (CH_2_), 27.21 (CH_2_), 26.58 (CH_2_), 25.88 (CH_3_), 20.61 (CH_3_), 17.77 (CH_3_), 16.34 (CH_3_), 16.10 (CH_3_) ppm.

**Synthesis of (2*E*,6*E*,10*E*)-12-hydroxy-3,7,11-trimethyldodeca-2,6,10-trien-1-yl acetate (S2)**

To a CH_2_Cl_2_ (20 mL, room temperature) suspension of SeO_2_ (111 mg, 1.0 mmol, 0.1 eq) was added *t*-BuOOH (3.6 mL, 5.5 M in decane, 20.0 mmol, 2.0 eq) dropwise. The mixture was stirred at room temperature for 0.5 h, followed by the addition of **S1** (2.64 g, 10.0 mmol) dropwise. The reaction mixture was stirred at room temperature for 24 h. Then the reaction solution was concentrated under reduced pressure. The residue was dissolved in Et_2_O (100 mL). The organic layer was washed with sat. NaHCO_3_ (50 mL), dried with MgSO_4_ and concentrated by vacuum evaporation.

The residue was re-dissolved in methanol/THF (1:4, 20 mL), and the mixture was cooled to 0 °C, then NaBH_4_ (0.75 g, 20.0 mmol, 2.0 eq) was added into the mixture in three batches. The reaction was stirred at 0 °C for 1 h, and then quenched by pouring into aq. NH_4_Cl (50 mL sat. NH_4_Cl with 100 mL ice-water). The product was extracted with Et_2_O (3 x 80 mL), and the extracts were dried with MgSO_4_ and concentrated under reduced pressure. Purification via flash chromatography (cyclohexane/ethyl acetate, 4:1, *R*_f_ = 0.23) provided compound **S2** (0.74 g, 2.64 mmol, 26%) as a colourless oil. EI-MS (70 eV): *m*/*z* (%) = 220 (1), 202 (1), 187 (3), 161 (3), 147 (3), 135 (9), 119 (10), 107 (24), 93 (39), 81 (16), 68 (27), 55 (16), 43 (100). GC (HP5-MS): *I* = 2112. ^1^H NMR (500 MHz, C_6_D_6_): *δ* = 5.43 (tq, ^3^*J*_H,H_ = 7.1 Hz, ^4^*J*_H,H_ = 1.3 Hz, 1H), 5.38 (tq, ^3^*J*_H,H_ = 7.0 Hz, ^4^*J*_H,H_ = 1.4 Hz, 1H), 5.16 (tq, ^3^*J*_H,H_ = 5.2 Hz, ^4^*J*_H,H_ = 1.3 Hz, 1H), 4.61 (d, ^3^*J*_H,H_ = 7.2 Hz, 2H), 3.81 (d, ^3^*J*_H,H_ = 3.8 Hz, 2H), 2.14 – 2.10 (m, 2H), 2.09 – 2.02 (m, 4H), 1.99 – 1.93 (m, 2H), 1.70 (s, 3H), 1.56 (d, ^4^*J*_H,H_ = 1.4 Hz, 3H), 1.53 (d, ^4^*J*_H,H_ = 1.5 Hz, 3H), 1.51 (d, ^4^*J*_H,H_ = 1.5 Hz, 3H), 1.03 (m, 1H) ppm. ^13^C NMR (126 MHz, C_6_D_6_): *δ* = 170.22 (C_q_), 141.55 (C_q_), 135.51 (C_q_), 135.18 (C_q_), 125.11 (CH), 124.41 (CH), 119.55 (CH), 68.71 (CH_2_), 61.25 (CH_2_), 39.81 (CH_2_), 39.77 (CH_2_), 26.53 (CH_2_), 26.49 (CH_2_), 20.62 (CH_3_), 16.33 (CH_3_), 16.08 (CH_3_), 13.70 (CH_3_) ppm.

**Synthesis of (((2*E*,6*E*)-3,7,11-trimethyldodeca-2,6,10-trien-1-yl)sulfonyl)benzene (S5)**

To a Et_2_O (20 mL, 0 °C) solution of (2*E*,6*E*)-farnesol (3.00 g, 13.5 mmol) was added PBr_3_ (1.46 g, 5.4 mmol, 0.4 eq) dropwise. The mixture was stirred at 0 °C for 45 min, and then quenched by pouring into ice-water (150 mL). The mixture was extracted with Et_2_O (3 x 50 mL). The combined extracts were dried with MgSO_4_ and concentrated under reduced pressure to afford crude **S4**, which was used for the next step directly.

Sodium benzenesulfinate (2.89 g, 17.6 mmol, 1.3 eq) was added into DMF (45 mL) at room temperature, followed by the addition of **S4** dropwise. The mixture was stirred overnight and then quenched by pouring into water (100 mL). The product was extracted with Et_2_O (3 x 100 mL). The organic layers were combined, washed with sat. NH_4_Cl and brine, dried with MgSO_4_ and concentrated under reduced pressure. The residue was purified by column chromatography (cyclohexane/ethyl acetate, 6:1, *R*_f_ = 0.33) to give compound **S5** (3.74 g, 10.8 mmol, 80% over 2 steps) as a colorless oil. EI-MS (70 eV): *m*/*z* (%) = 346 (5), 205 (5), 189 (1), 161 (8), 149 (6), 137 (14), 121 (9), 107 (9), 93 (17), 81 (43), 69 (100), 53 (11), 41 (38). GC (HP5-MS): *I* = 2758. ^1^H NMR (500 MHz, C_6_D_6_): *δ* = 7.82 – 7.75 (m, 2H), 7.00 – 6.87 (m, 3H), 5.23 (dp, ^3^*J*_H,H_ = 7.0 Hz, ^4^*J*_H,H_ =1.4 Hz, 1H), 5.16 – 5.10 (m, 2H), 3.47 (d, ^3^*J*_H,H_ = 7.9 Hz, 2H), 2.23 – 2.12 (m, 2H), 2.10 – 2.03 (m, 2H), 1.99 – 1.91 (m, 2H), 1.87 – 1.79 (m, 2H), 1.68 (d, ^4^*J*_H,H_ = 1.3 Hz, 3H), 1.57 (d, ^4^*J*_H,H_ = 1.2 Hz, 3H), 1.54 (d, ^4^*J*_H,H_ = 1.3 Hz, 3H), 1.06 (d, ^4^*J*_H,H_ = 1.3 Hz, 3H) ppm. ^13^C NMR (126 MHz, C_6_D_6_): *δ* = 145.39 (C_q_), 140.19 (C_q_), 135.63 (C_q_), 132.92 (CH), 131.33 (C_q_), 128.86 (2 x CH), 128.79 (2 x CH), 124.84 (CH), 124.04 (CH), 111.59 (CH), 56.16 (CH_2_), 40.20 (CH_2_), 39.93 (CH_2_), 27.20 (CH_2_), 26.55 (CH_2_), 25.90 (CH_3_), 17.80 (CH_3_), 16.10 (CH_3_), 16.07 (CH_3_) ppm.

**Synthesis of (2*E*,6*E*,10*E*,14*E*,18*E*)-3,7,11,15,19,23-hexamethyl-13-(phenylsulfonyl) tetracosa-2,6,10,14,18,22-hexaen-1-ol (S7)**

This compound was synthesized according to a published procedure^26^. To a Et_2_O (6 mL, 0 °C) solution of **S2** (0.74 g, 2.64 mmol) was added PBr_3_ (0.28 g, 1.05 mmol, 0.4 eq). After stirring for 45 min, the reaction was quenched by pouring into ice-water (150 mL), followed by extraction with Et_2_O (3 x 60 mL). The extracts were dried with MgSO_4_ and concentrated under reduced pressure to afford crude product **S3**, which was used for next reaction directly without further purification.

Sulfone **S5** (1.01g, 2.90 mmol, 1.1 eq) was dissolved in a mixture solution of THF/HMPA (4:1, 15 mL). After cooling the mixture to –78 °C, *n*-BuLi (1.98 mL, 1.6 M in hexane, 3.17 mmol, 1.2 eq) was added dropwise. The mixture was stirred for 1.5 h, and **S3** was added into the mixture dropwise. The reaction solution was stirred overnight without further cooling. The reaction was quenched by pouring into an ice-cold aqueous NH_4_Cl solution (40 mL sat. NH_4_Cl with 100 mL ice-water). The product was extracted with Et_2_O (3 x 50 mL), the combined extracts were washed with sat. NaCl, dried with MgSO_4_, and concentrated under reduced pressure to provide the crude compound **S6**.

Sulfone **S6** was dissolved in methanol (20 mL) and NaOH (5 mL, 1 M in H_2_O) was added. The reaction mixture was stirred at room temperature for 4 h and monitored by TLC to confirm the reaction was completed. Then the reaction mixture was poured into ice-water (150 mL), and the product was extracted with Et_2_O (3 x 50 mL). The combined extracts were washed with brine, dried with MgSO_4_ and concentrated under reduced pressure. Purification via silica gel chromatography (cyclohexane/ethyl acetate, 2:1, *R*_f_ = 0.46) gave product **S7** (1.05 g, 1.85 mmol, 70% over 3 steps) as a colourless oil. ^1^H NMR (500 MHz, C_6_D_6_): *δ* = 7.90 – 7.82 (m, 2H), 7.01 – 6.90 (m, 3H), 5.43 (tq, ^3^*J*_H,H_ = 6.7 Hz, ^4^*J*_H,H_ = 1.3 Hz, 1H), 5.24 (tp, ^3^*J*_H,H_ = 6.9 Hz, ^4^*J*_H,H_ = 1.4 Hz, 1H), 5.23 – 5.13 (m, 3H), 5.09 (dq, ^3^*J*_H,H_ = 10.3 Hz, ^4^*J*_H,H_ = 1.3 Hz, 1H), 4.03 (dd, ^3^*J*_H,H_ = 6.5 Hz, ^3^*J*_H,H_ = 3.7 Hz, 2H), 3.98 (td, ^3^*J*_H,H_ = 10.7 Hz, ^3^*J*_H,H_ = 3.2 Hz, 1H), 3.18 (d, ^3^*J*_H,H_ = 13.2 Hz, 1H),2.50 (dd, ^2^*J*_H,H_ = 13.4 Hz, ^3^*J*_H,H_ = 11.2 Hz, 1H), 2.21 – 2.03 (m, 9H), 2.02 – 1.86 (m, 7H), 1.68 (d, ^4^*J*_H,H_ = 1.4 Hz, 3H), 1.58 (d, ^4^*J*_H,H_ = 1.5 Hz, 3H), 1.57 (d, ^4^*J*_H,H_ = 1.5 Hz, 3H), 1.53 (d, ^4^*J*_H,H_ = 1.3 Hz, 3H), 1.49 (d, ^4^*J*_H,H_ = 1.4 Hz, 3H), 1.43 (d, ^4^*J*_H,H_ = 1.3 Hz, 3H), 1.11 (d, ^4^*J*_H,H_ = 1.4 Hz, 3H) ppm. ^13^C NMR (126 MHz, C_6_D_6_): *δ* = 144.41 (C_q_), 139.35 (C_q_), 137.79 (C_q_), 135.62 (C_q_), 134.96 (C_q_), 132.91 (CH), 131.36 (C_q_), 130.55 (C_q_), 129.69 (2 x CH), 128.63 (2 x CH), 125.20 (CH), 124.82 (CH), 124.69 (CH), 124.18 (CH), 118.67 (CH), 63.93 (CH), 59.41 (CH_2_), 40.23 (CH_2_), 40.08 (CH_2_), 39.85 (CH_2_), 39.84 (CH_2_), 38.20 (CH_2_), 27.26 (2 x CH_2_), 27.03 (CH_2_), 26.84 (CH_2_), 26.65 (CH_2_), 25.90 (CH_3_), 17.81 (CH_3_), 16.47 (CH_3_), 16.22 (CH_3_), 16.11 (CH_3_), 16.06 (CH_3_), 15.97 (CH_3_) ppm.

**Synthesis of (2*E*,6*E*,10*E*,14*E*,18*E*)-3,7,11,15,19,23-hexamethyltetracosa-2,6,10,14,18,22-hexaen-1-ol (S8)**

Compound **S7** (0.50 g, 0.88 mmol) and Pd(dppp)Cl_2_ (53 mg, 0.09 mmol, 0.1 eq) were added into THF (3 mL. The mixture was cooled to 0 °C, and LiEt_3_BH (1.55 mL, 1.7 M in THF, 2.64 mmol, 3.0 eq) was added dropwise. After stirring for 0.5 h, the reaction was quenched by pouring into aqueous NH_4_Cl (40 mL sat. NH_4_Cl with 100 mL ice-water). The product was extracted with Et_2_O (3 x 60 mL). The combined extracts were dried with MgSO_4_ and concentrated by vacuum evaporation. The product (0.28 g, 0.66 mmol, 75%) was purified by column chromatography (cyclohexane/ethyl acetate, 4:1, *R*_f_ = 0.45) and was obtained as a colourless oil. EI-MS (70 eV): *m*/*z* (%) = 408 (1), 339 (1), 271 (2), 257 (1), 243 (1), 229 (1), 203 (4), 189 (6), 175 (3), 161 (9), 147 (8), 135 (17), 121 (17), 107 (20), 93 (25), 81 (25), 69 (100), 55 (12), 41 (30). GC (HP5-MS): *I* = 3141. ^1^H NMR (500 MHz, C_6_D_6_): *δ* = 5.40 (tq, ^3^*J*_H,H_ = 6.7 Hz, ^4^*J*_H,H_ =1.3 Hz, 1H), 5.34 – 5.28 (m, 3H), 5.24 (tp, ^3^*J*_H,H_ = 6.8 Hz, ^4^*J*_H,H_ = 1.4 Hz, 2H), 3.98 (t, ^3^*J*_H,H_ = 5.6 Hz, 2H), 2.22 – 2.17 (m, 8H), 2.15 – 2.09 (m, 10H), 2.04 – 1.98 (m, 2H), 1.68 (d, ^4^*J*_H,H_ = 1.5 Hz, 3H), 1.63 – 1.60 (m, 9H), 1.59 (d, ^4^*J*_H,H_ = 1.3 Hz, 3H), 1.57 (d, ^4^*J*_H,H_ = 1.3 Hz, 3H), 1.48 (d, ^4^*J*_H,H_ = 1.3 Hz, 3H), 0.62 – 0.52 (m, 1H) ppm. ^13^C NMR (126 MHz, C_6_D_6_): *δ* = 138.16 (C_q_), 135.35 (C_q_), 135.13 (C_q_), 135.07 (C_q_), 135.03 (C_q_), 131.15 (C_q_), 124.99 (CH), 124.98 (CH), 124.86 (2 x CH), 124.81 (CH), 124.50 (CH), 59.40 (CH_2_), 40.28 (CH_2_), 40.27 (CH_2_), 40.26 (CH_2_), 40.24 (CH_2_), 39.93 (CH_2_), 27.30 (CH_2_), 27.22 (CH_2_), 27.19 (CH_2_), 27.17 (CH_2_), 26.82 (CH_2_), 25.90 (CH_3_), 17.79 (CH_3_), 16.23 (2 x CH_3_), 16.19 (CH_3_), 16.18 (CH_3_), 16.16 (CH_3_) ppm.

**Synthesis of trisammonium farnesylfarnesyl prophosphate (HexPP, S10)**

Alcohol **S8** (100 mg, 0.23 mmol) was converted into **S9** following the same procedure as described in the synthesis of compound **S4**. The obtained crude **S9** was used for phosphorylation without further purification.

(NBu_4_)_3_HP_2_O_7_ (312 mg, 0.35 mmol, 1.5 eq) was dissolved in acetonitrile (0.5 mL), and **S9** (mixed with 1 mL acetonitrile) was added dropwise. The mixture was stirred overnight and the solvent was removed under reduced pressure. The residue was purified via silica gel chromatography (*i*PrOH/25% NH_4_OH/H_2_O, 6:2.5:0.5, *R*_f_ = 0.1) to provide the colourless solid **S10** (100 mg, 0.15 mmol, 68%). HRMS (ESI): *m*/*z* = 585.3119 (calc. for [C_30_H_51_O_7_P_2_]^–^: 585.3116). ^1^H NMR (700 MHz, D_2_O): *δ* = 5.31 (s, 1H), 5.11 – 4.94 (m, 5H), 4.40 (s, 2H), 2.02 – 1.79 (m, 20H), 1.63 (s, 3H), 1.55 (s, 3H), 1.53 (s, 3H), 1.52 – 1.47 (m, 12H) ppm. ^13^C NMR (176 MHz, D_2_O): *δ* = 140.18 (C_q_), 134.70 (C_q_), 134.35 (C_q_), 134.23 (2 x C_q_), 130.44 (C_q_), 124.98 (CH), 124.37 (CH), 124.33 (CH), 124.30 (CH) 124.22 (CH), 120.74 (d, ^3^*J*_C,P_ = 7.0 Hz, CH), 62.51 (d, ^2^*J*_C,P_ = 6.4 Hz, CH_2_), 39.91 (CH_2_), 39.80 (CH_2_), 39.76 (CH_2_), 39.71 (2 x CH_2_), 26.99 (CH_2_), 26.80 (CH_2_), 26.73 (2 x CH_2_), 26.62 (CH_2_), 25.45 (CH_3_), 17.37 (CH_3_), 16.13 (CH_3_), 15.80 (CH_3_), 15.77 (2 x CH_3_), 15.74 (CH_3_) ppm. ^31^P NMR (202 MHz, D_2_O): *δ* = –10.54 (d, ^2^*J*_P,P_ = 18.8 Hz), –11.32 (d, ^2^*J*_P,P_ = 18.7 Hz) ppm.

**Determination of absolute configurations of terpenes through a stereoselective deuterium labeling approach**

This method makes use of the enzymatic introduction of stereoselective anchors at CH_2_ groups of known configuration with exchange of one of the two diastereotopic hydrogens against deuterium. For this purpose the stereoselectively deuterated substrates (*E*)- and (*Z*)-(4-^13^C,4-^2^H)IPP and (*R*)- and (*S*)-(1-^13^C,1-^2^H)IPP are used. Their incorporation into oligoprenyl diphosphates follows a known stereochemical course^27^, so that the configurations at the deuterated carbons are known. The relative orientation of naturally present stereogenic centers in a terpene with respect to the introduced anchors at the deuterated carbons can be determined by NOESY, which allows to conclude on the absolute configuration of the natural product. Additional ^13^C-labelings at the deuterated carbons allow for a sensitive detection by HSQC spectroscopy.


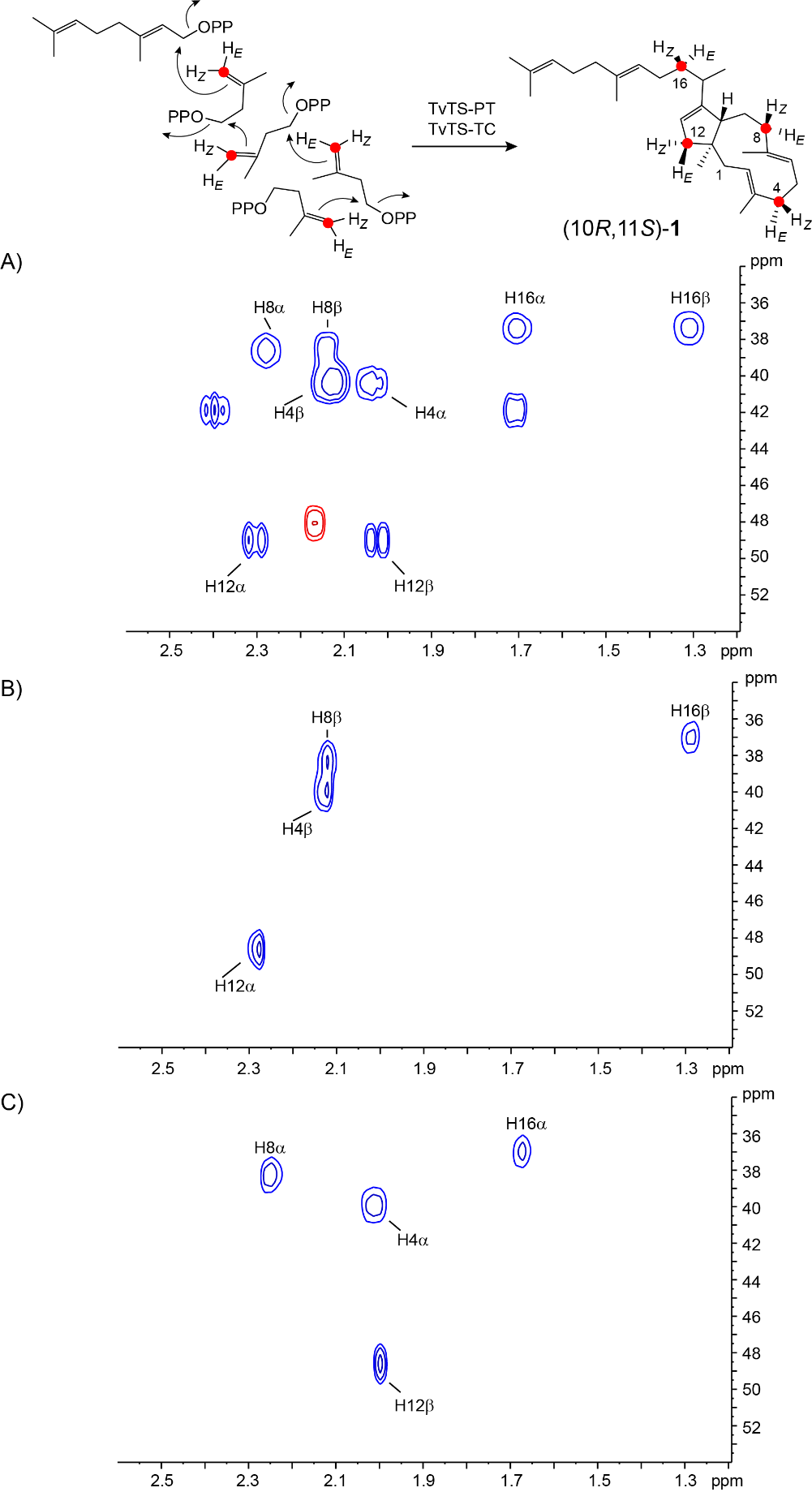


**Supplementary Figure 16 │ The absolute configuration of 1.** Partial HSQC spectra of A) unlabelled **1** and from incubation experiments of TvTS with GPP and B) (*E*)-(4-^13^C,4-^2^H)IPP and C) (*Z*)-(4-^13^C,4-^2^H)IPP. Red dots indicate ^13^C-labelled carbon atoms. Key NOESY correlations to the diastereotopic hydrogens at the labelled carbons used for the assignment of the absolute configuration of **1** are shown in Supplementary Figure 5.


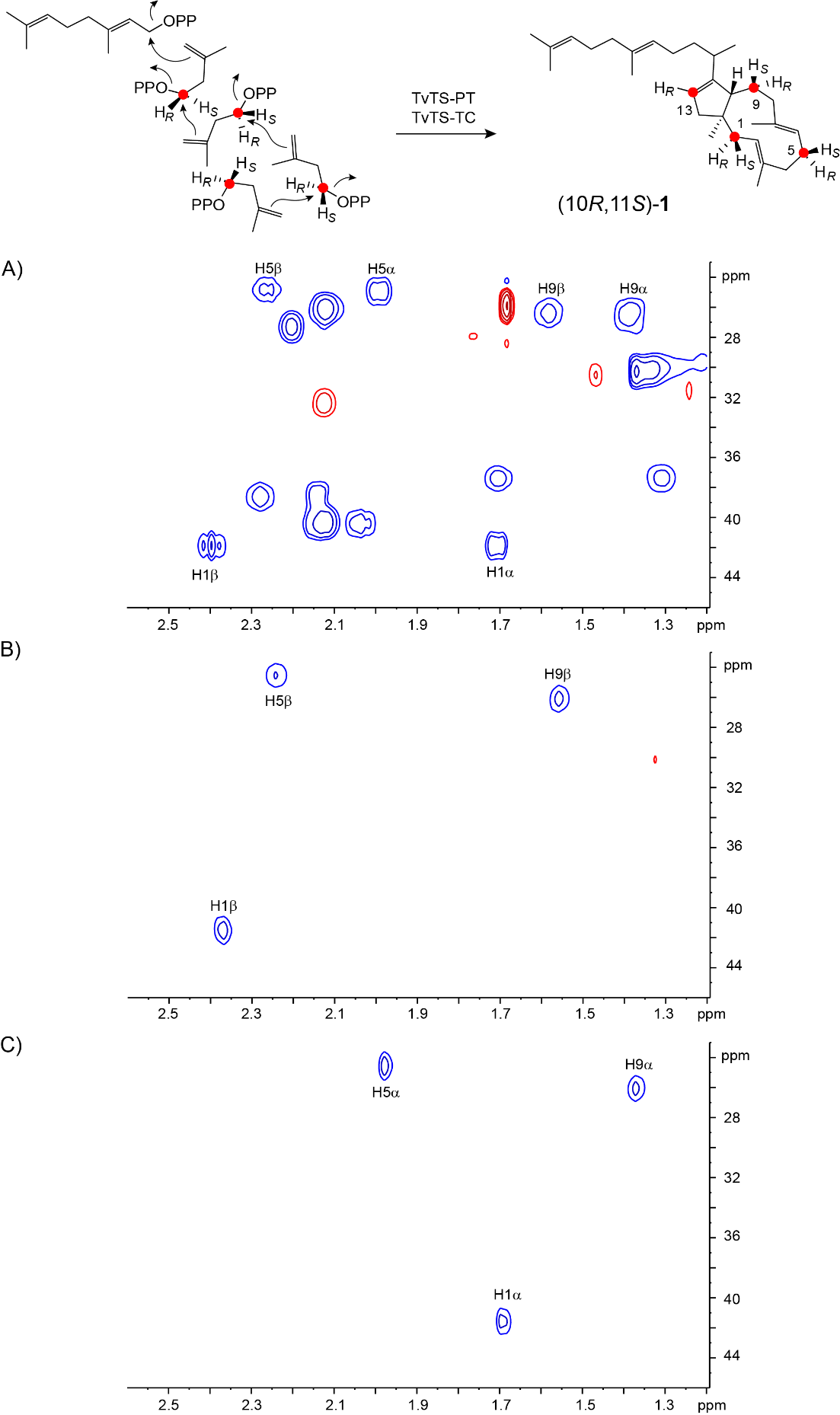


**Supplementary Figure 17 │ The absolute configuration of 1.** Partial HSQC spectra of A) unlabeled **1** and from incubation experiments of TvTS with GPP and B) (*R*)-(1-^13^C,1-^2^H)IPP and C) (*S*)-(1-^13^C,1-^2^H)IPP. Red dots indicate ^13^C-labelled carbon atoms. Key NOESY correlations to the diastereotopic hydrogens at the labelled carbons used for the assignment of the absolute configuration of **1** are shown in Supplementary Figure 5.


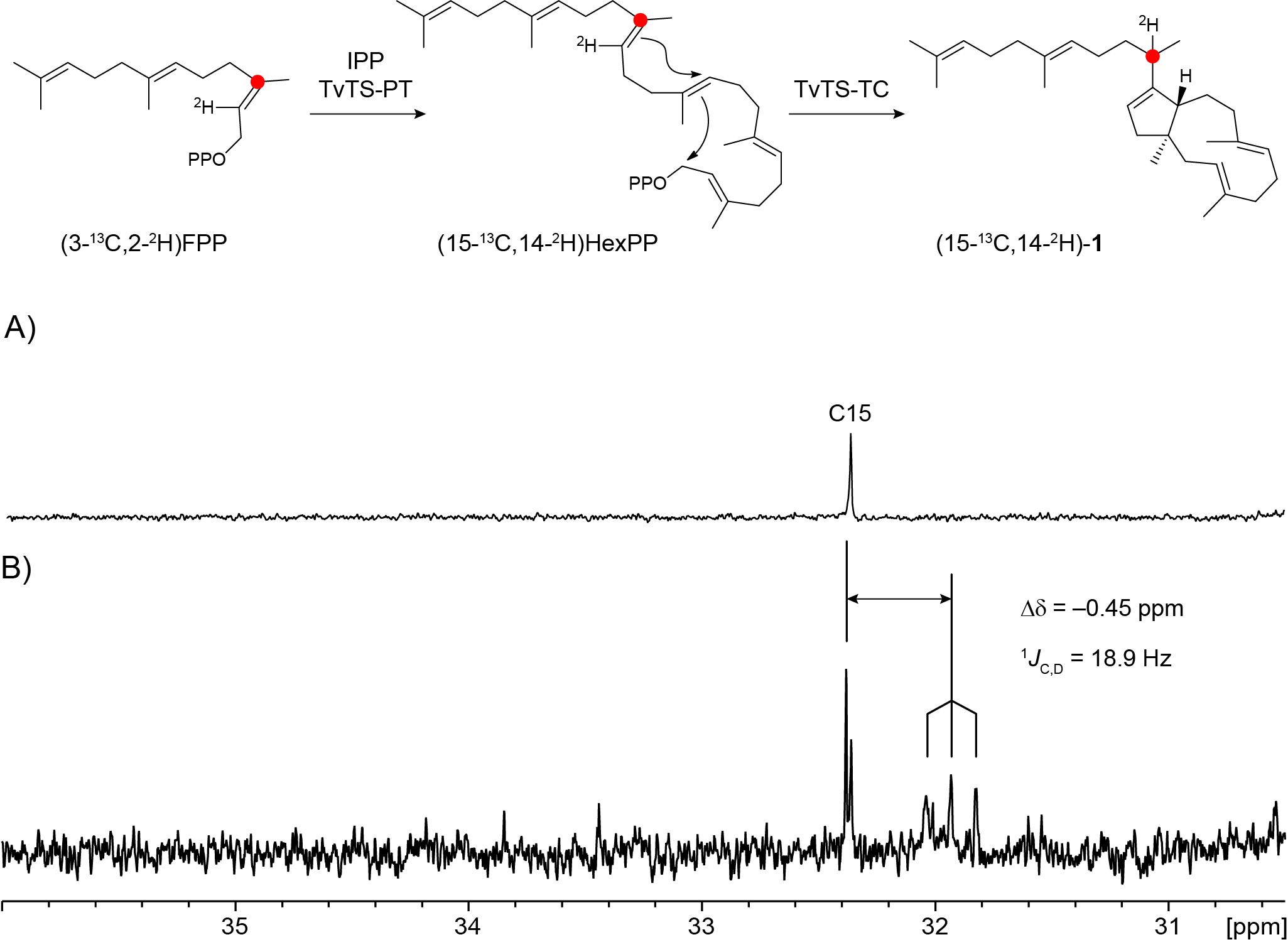


**Supplementary Figure 18 │ The 1,2-hydride shift in the biosynthesis of 1.** ^13^C-NMR spectra of A) unlabeled **1** and B) incubation of TvTS with (3-^13^C,2-^2^H)FPP and IPP. The upfield shifted triplet indicates a direct C-D bond, resulting from the hydride shift.


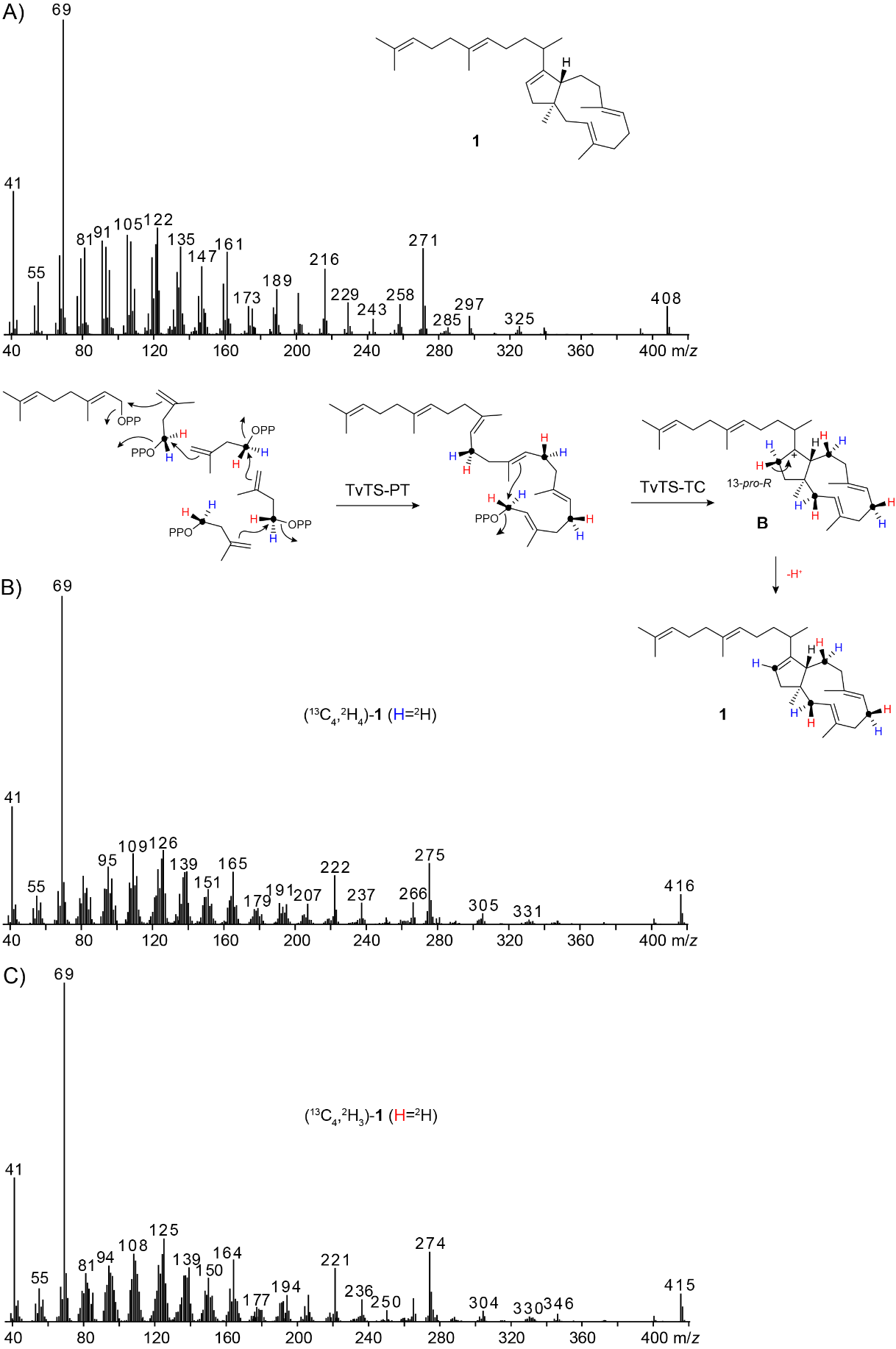


**Supplementary Figure 19 │ The deprotonation step in the biosynthesis of 1.** EI mass spectra of A) unlabeled **1**, and labeled **1** obtained with TvTS-PT and TvTS-TC from GPP and B) (*R*)-(1-^13^C,1-^2^H)IPP (blue H = ^2^H) and C) (*S*)-(1-^13^C,1-^2^H)IPP (red H = ^2^H), revealing specific loss of the 1-*pro*-*S* hydrogen of IPP in the deprotonation step to **1** (equal to the 13-*pro*-*R* in cation **B**).

**
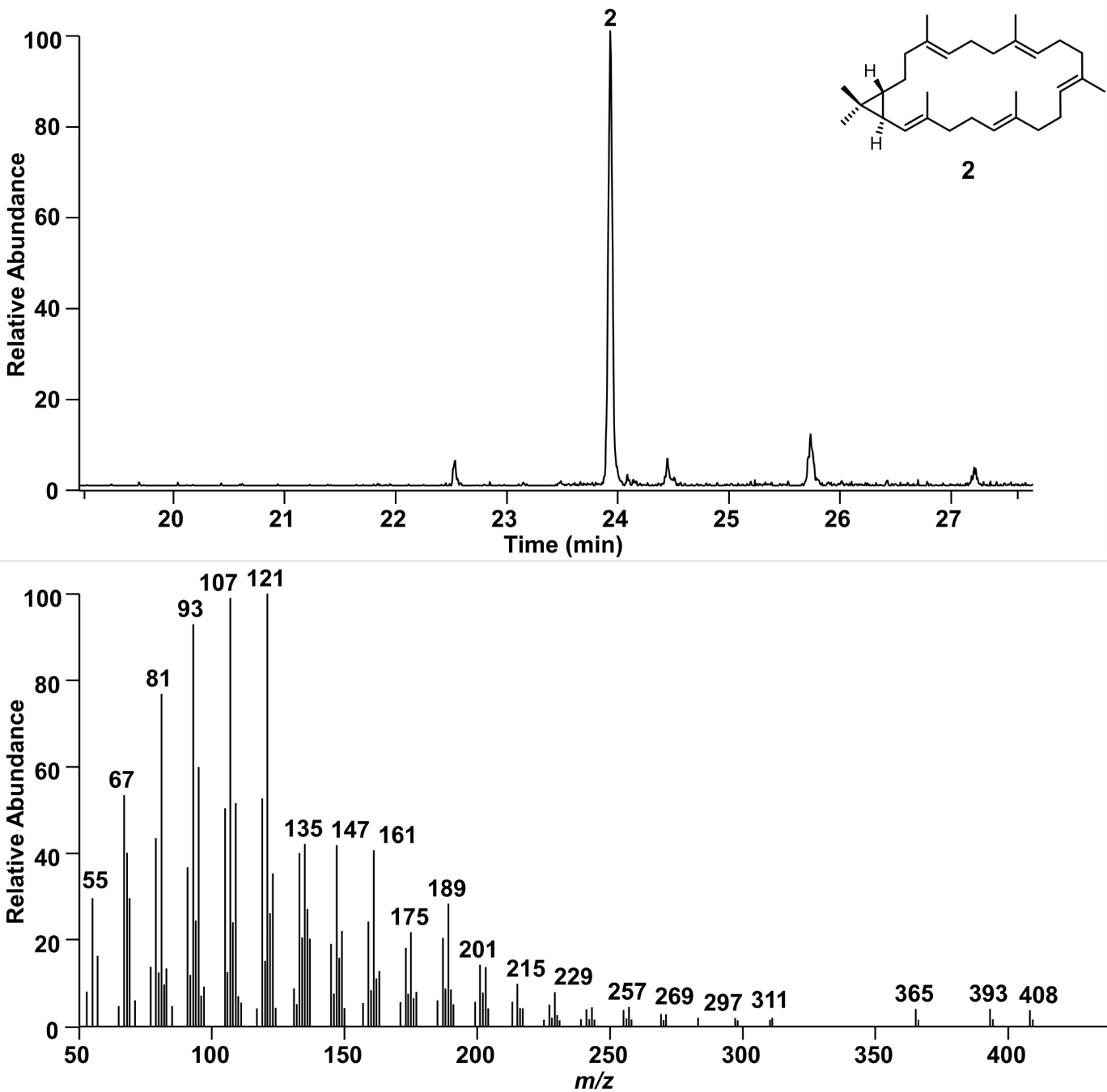
**

**Supplementary Figure 20 │ Production of 2 by *S. cerevisiae* XM018.** Total ion chromatogram of an extract obtained from a culture of *S. cerevisiae* XM018.


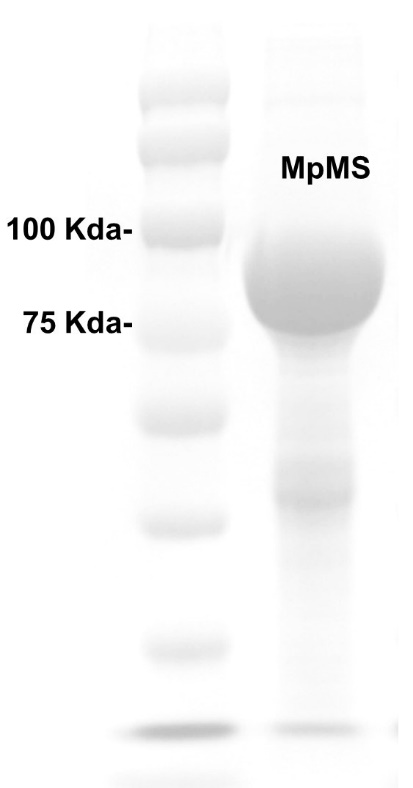


**Supplementary Figure 21 │ SDS-PAGE of MpMS for in vitro assay.** Protein was purified at least three times throughout the course of the work to a similar result.


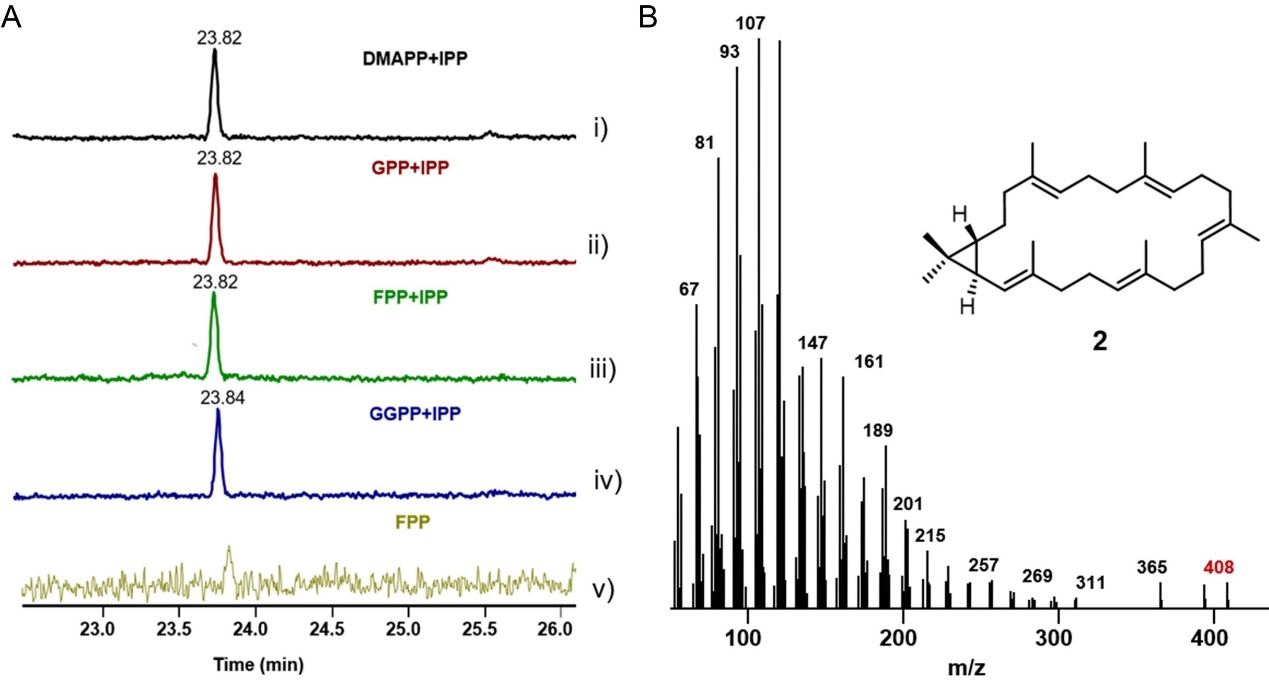


**Supplementary Figure 22 │Functional characterization of MpMS in vitro.** A) GC-EI-MS ion chromatograms of extracts from an incubation of recombinant MpMS with i) DMAPP and IPP, ii) GPP+IPP, iii) FPP+IPP, iv) GGPP+IPP, v) FPP. B) Mass spectrometry of compound 2 detected by GC-EI-MS.

**Supplementary Figure 23 │ Structure elucidation of 2.** Carbon numbering follows HexPP numbering to indicate the biosynthetic origin of each carbon by same number. Bold: ^1^H-^1^H COSY correlations, single headed arrows: key HMBC correlations, double headed arrows: key NOESY correlations.

**Supplementary Table 6 │ NMR data of 2 recorded in C_6_D_6_ (500 MHz, 298 K).**

| C^[a]^ | | type | | ^13^C^[b]^ | | ^1^H^[b]^ | |
| --- | --- | --- | --- | --- | --- | --- | --- |
| 1 | | CH | | 30.69 | | 0.97 (dd, ^3^*J*_H,H_ = 8.1, 5.3 Hz, 1H) | |
| 2 | | CH | | 126.39 | | 5.04 (br d, ^3^*J*_H,H_ = 7.8 Hz, 1H) | |
| 3 | | C_q_ | | 134.73 | | – | |
| 4 | | CH_2_ | | 40.03 | | 2.10 – 2.17 (m, 2H) | |
| 5 | | CH_2_ | | 26.25 | | 2.18 – 2.22 (m, 2H) | |
| 6 | | CH | | 125.04 | | 5.24 – 5.30 (m, 2H) | |
| 7 | | C_q_ | | 134.32 | | – | |
| 8 | | CH_2_ | | 39.83 | | 2.10 – 2.17 (m, 2H) | |
| 9 | | CH_2_ | | 26.40 | | 2.18 – 2.22 (m, 2H) | |
| 10 | | CH | | 125.40 | | 5.24 – 5.30 (m, 2H) | |
| 11 | | C_q_ | | 134.19 | | – | |
| 12 | | CH_2_ | | 39.72 | | 2.10 – 2.17 (m, 2H) | |
| 13 | | CH_2_ | | 25.95 | | 2.18 – 2.22 (m, 2H) | |
| 14 | | CH | | 124.92 | | 5.24 – 5.30 (m, 2H) | |
| 15 | | C_q_ | | 134.49 | | – | |
| 16 | | CH_2_ | | 40.30 | | 2.10 – 2.17 (m, 2H) | |
| 17 | | CH_2_ | | 26.65 | | 2.18 – 2.22 (m, 2H) | |
| 18 | | CH | | 125.68 | | 5.24 – 5.30 (m, 2H) | |
| 19 | | C_q_ | | 134.33 | | – | |
| 20 | | CH_2_ | | 40.34 | | 2.10 – 2.17 (m, 2H) | |
| 21 | | CH_2_ | | 27.47 | | 1.70 (m, 1H, H_β_)  1.30 (m, 1H, H_α_) | |
| 22 | | CH | | 33.30 | | 0.54 (ddd, ^3^*J*_H,H_ = 8.7, 5.5, 5.5 Hz, 1H) | |
| 23 | | C_q_ | | 22.65 | | – | |
| 24 | | CH_3_ | | 21.91 | | 1.09 (s, 3H) | |
| 25 | | CH_3_ | | 23.36 | | 1.11 (s, 3H) | |
| 26 | | CH_3_ | | 15.759 | | 1.58 (s, 3H) | |
| 27 | | CH_3_ | | 15.90 | | 1.60 (s, 3H) | |
| 28 | | CH_3_ | | 15.763 | | 1.58 (s, 3H) | |
| 29 | | CH_3_ | | 15.89 | | 1.60 (s, 3H) | |
| 30 | | CH_3_ | | 16.59 | | 1.72 (d, ^4^*J*_H,H_ = 1.0, 3H) | |

[a] Carbon numbering follows numbering of HexPP and is shown in Supplementary Figure 23. [b] Chemical shifts *δ* in ppm, coupling constants *J* in Hertz, s = singlet, d = doublet, m = multiplet, br = broad. Diastereotopic hydrogens were assigned by labelling experiments (Supplementary Figure 35).


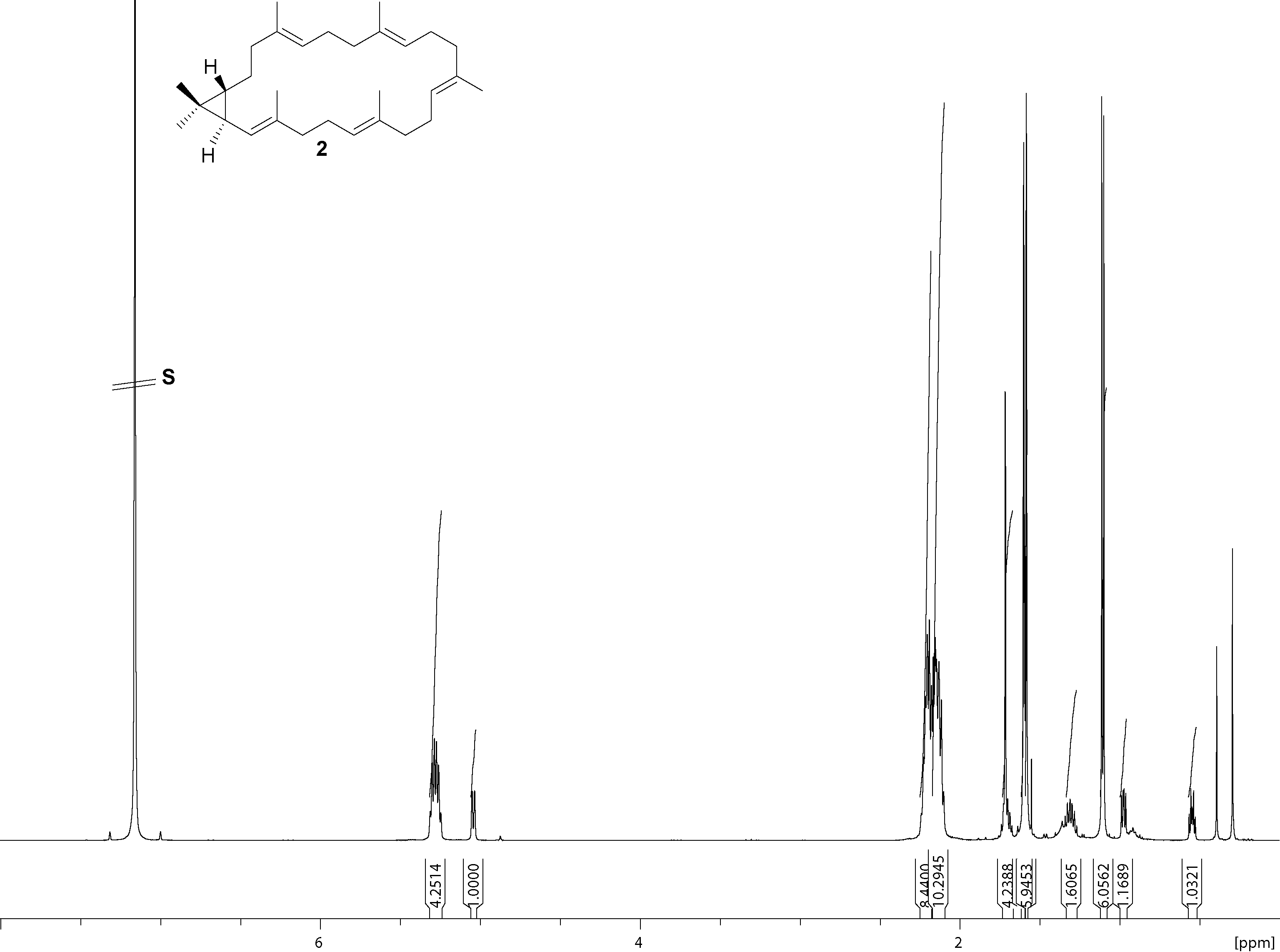


**Supplementary Figure 24 │ ^1^H-NMR spectrum of 2 (C_6_D_6_, 500 MHz).**


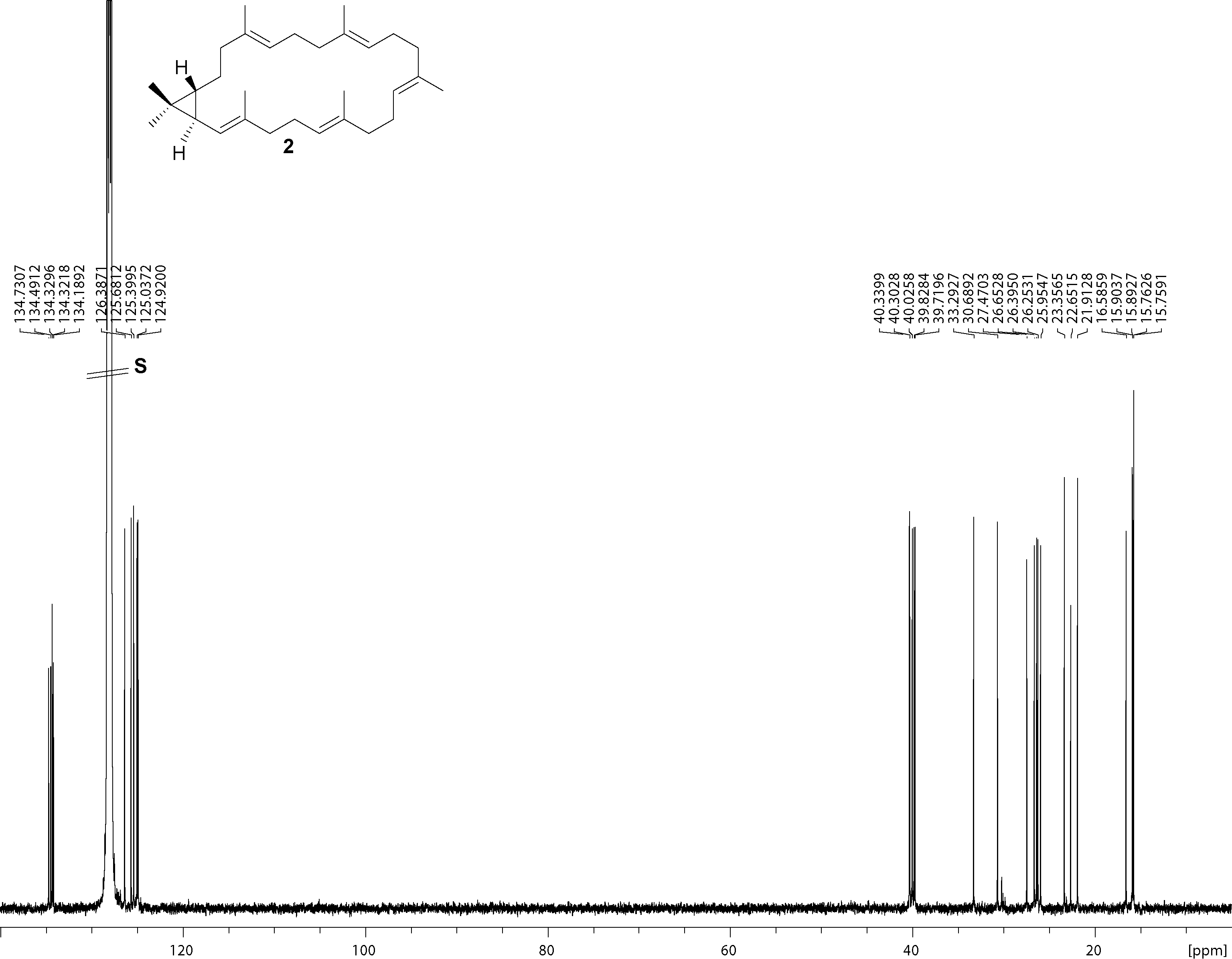


**Supplementary Figure 25 │ ^13^C-NMR spectrum of 2 (C_6_D_6_, 126 MHz).**


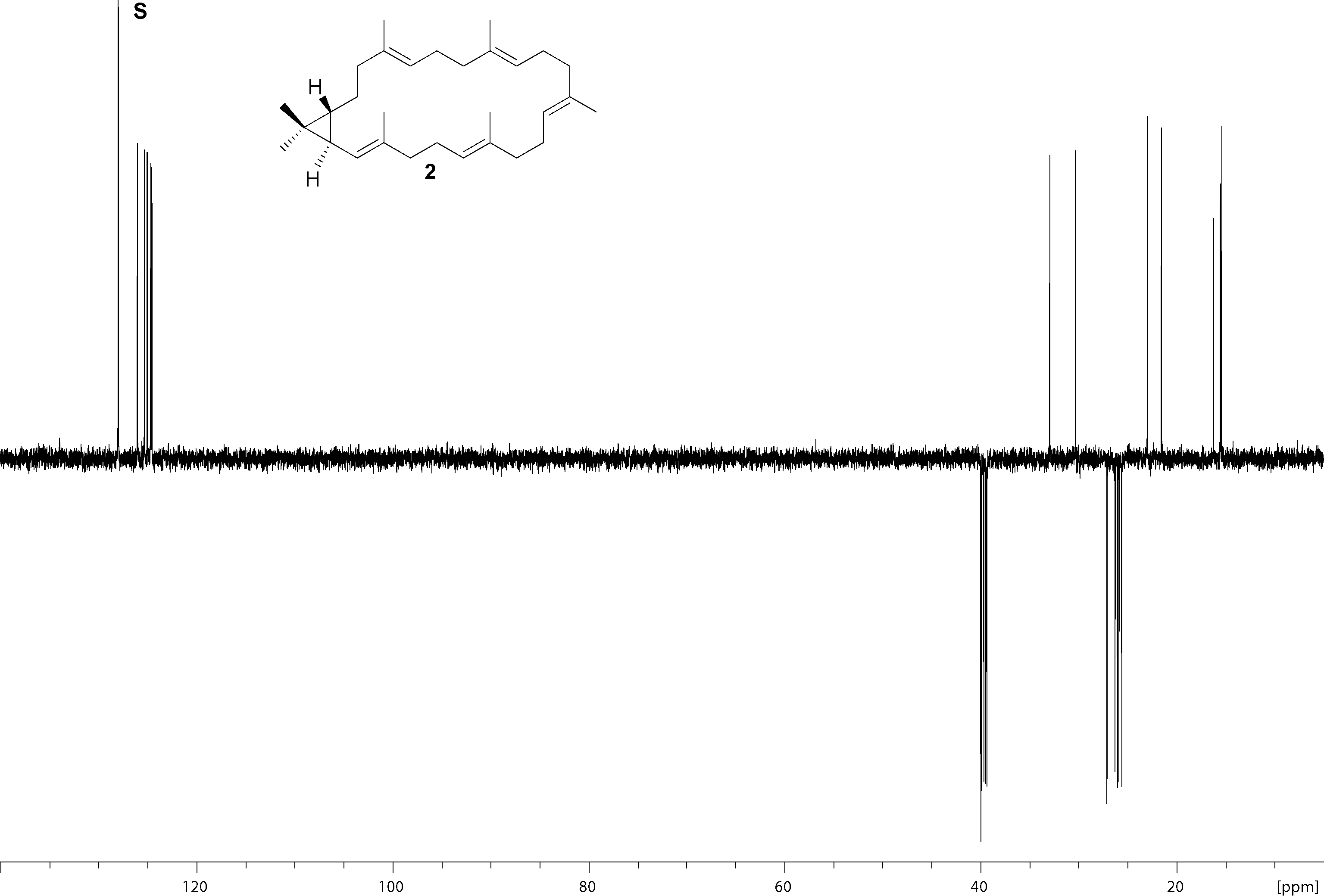


**Supplementary Figure 26 │ ^13^C-DEPT spectrum of 2 (C_6_D_6_, 126 MHz).**


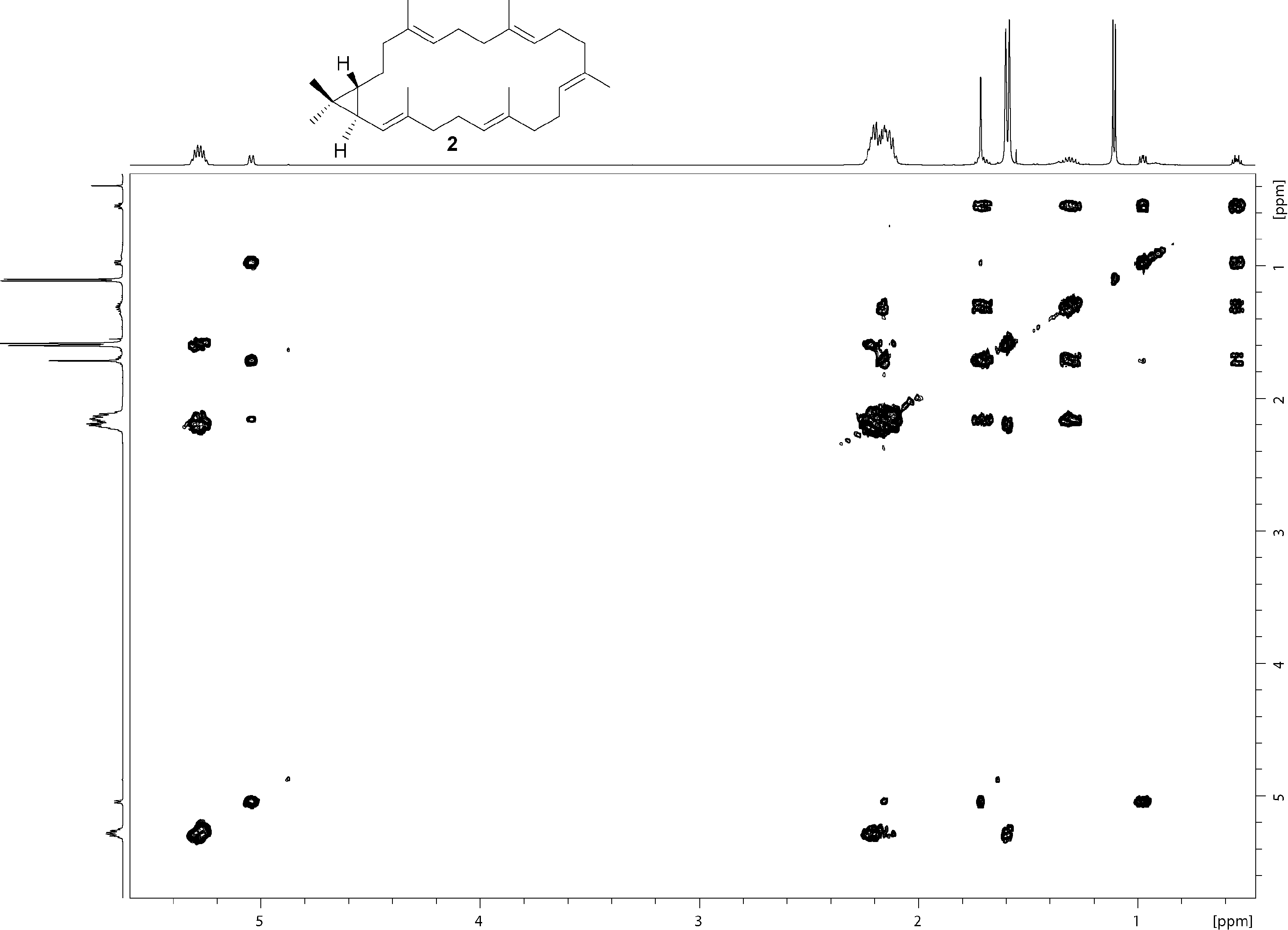


**Supplementary Figure 27 │ ^1^H-^1^H-COSY spectrum of 2 (C_6_D_6_).**


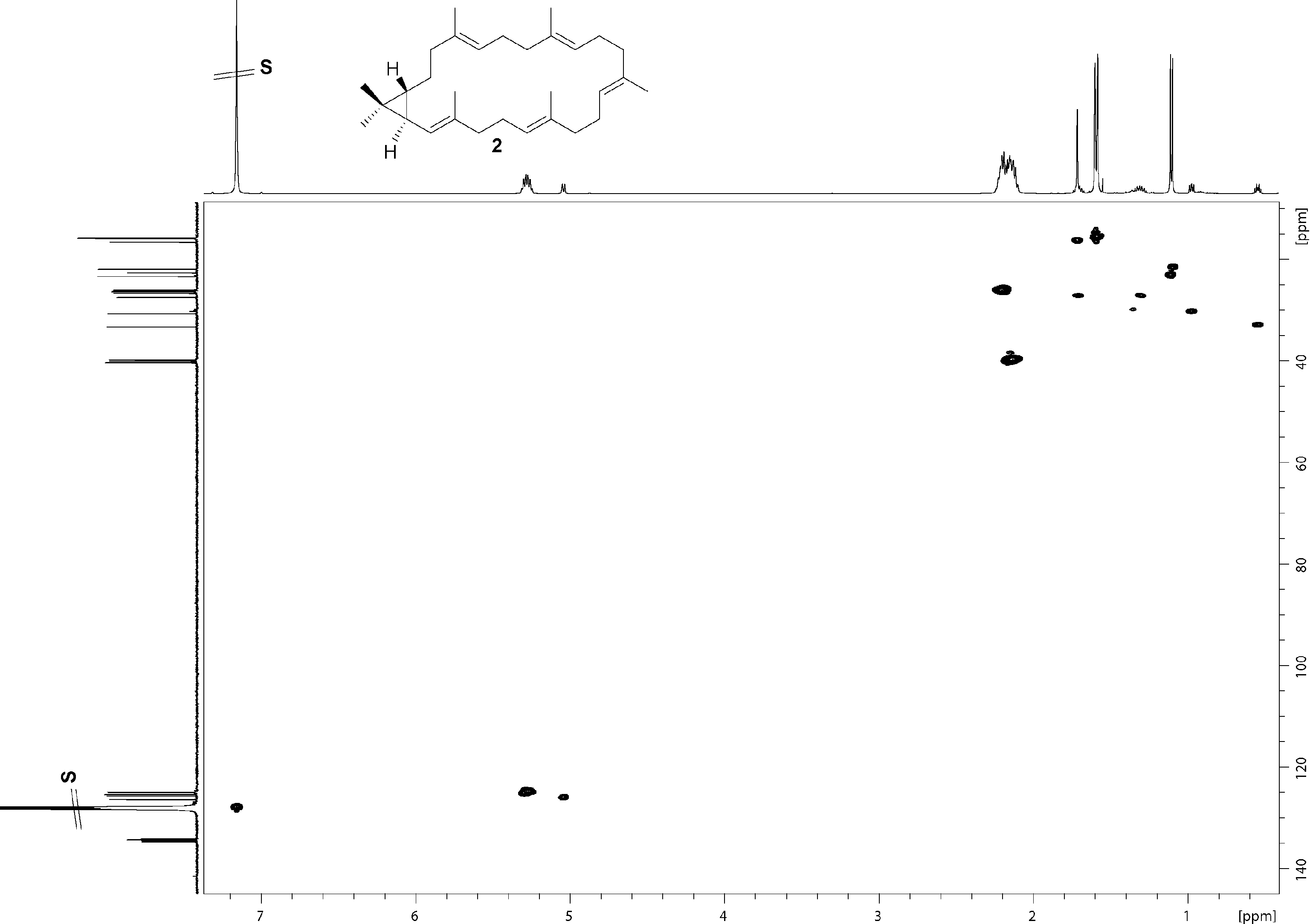


**Supplementary Figure 28 │ HSQC spectrum of 2 (C_6_D_6_).**


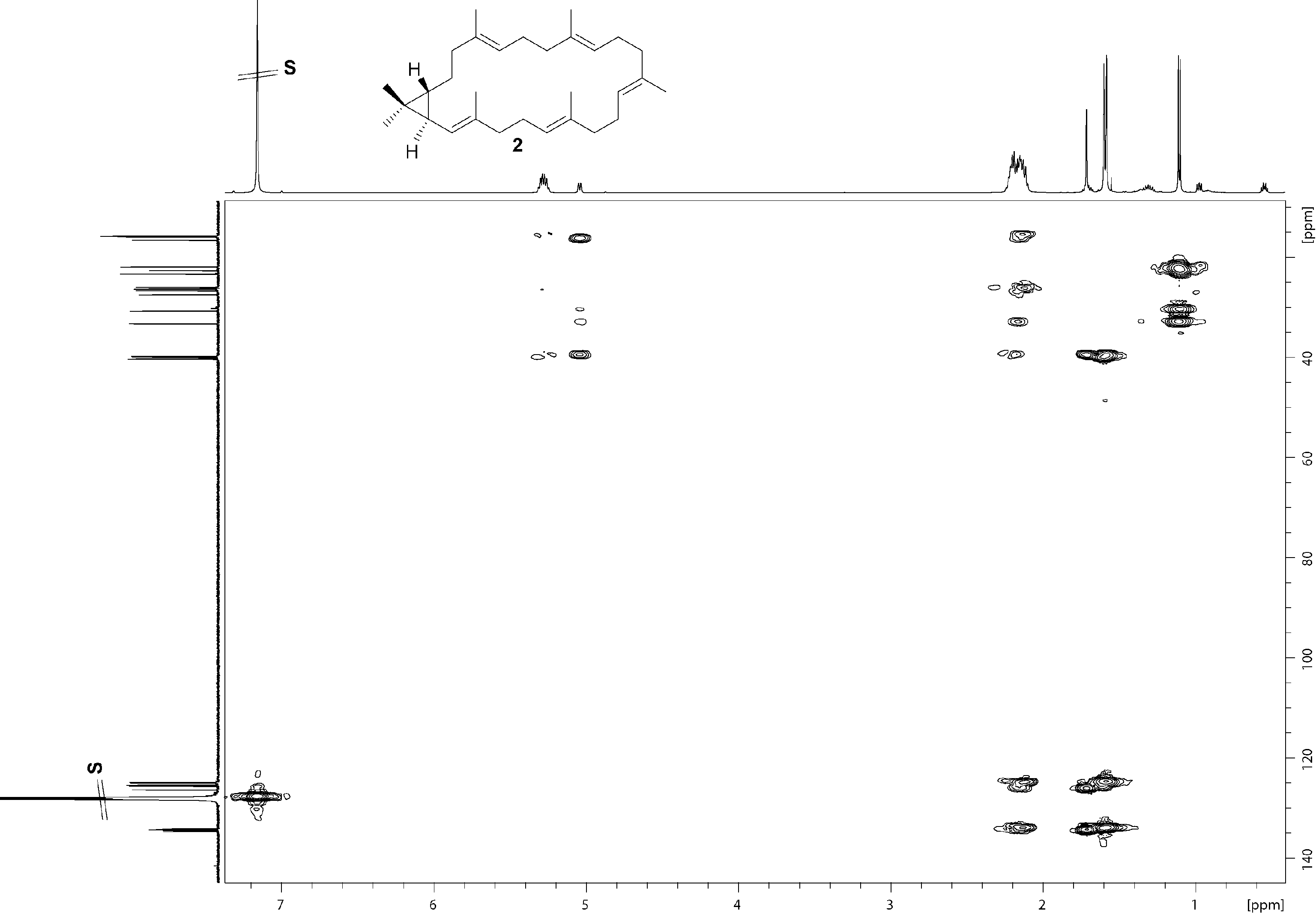


**Supplementary Figure 29 │ HMBC spectrum of 2 (C_6_D_6_).**


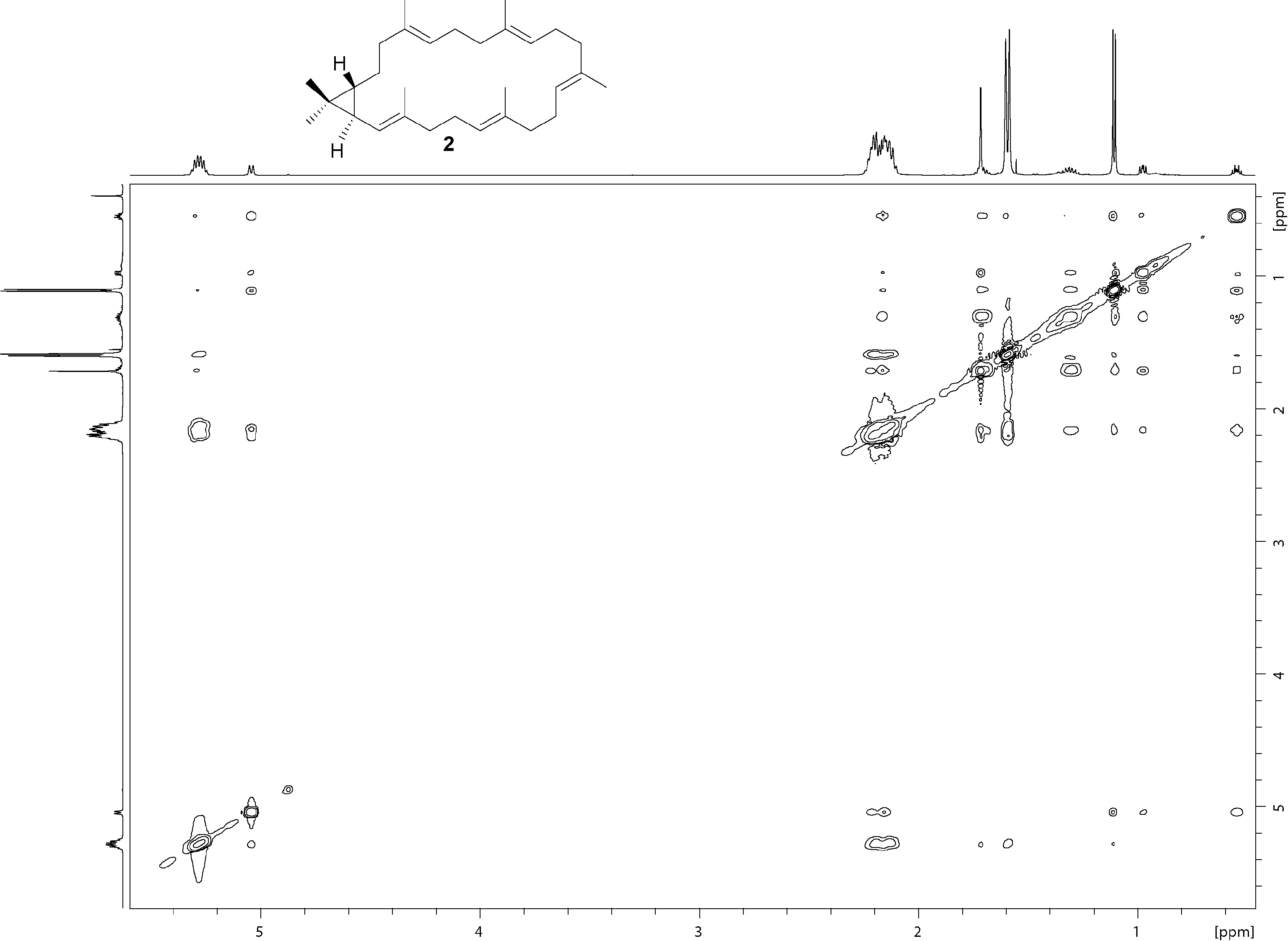


**Supplementary Figure 30 │ NOESY spectrum of 2 (C_6_D_6_).**


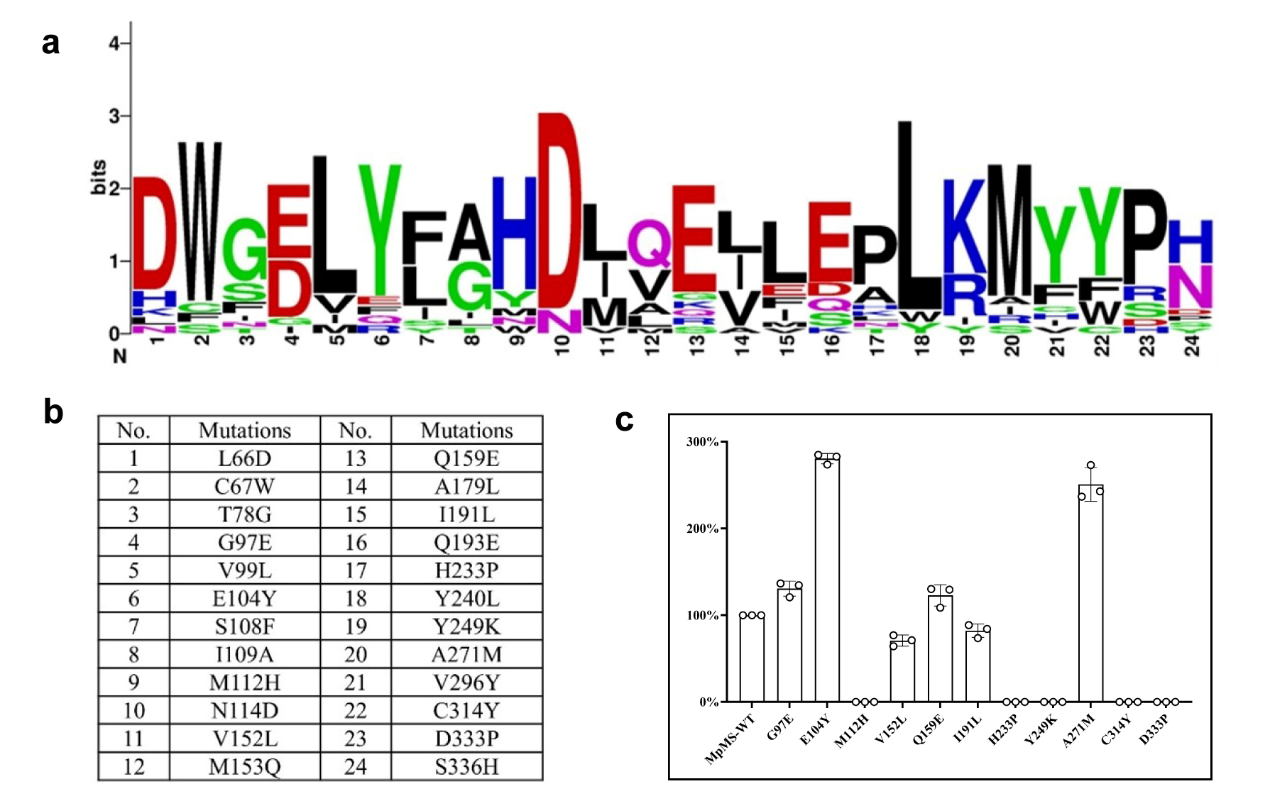


**Supplementary Figure 31 │ Protein engineering of the MPH_02178 gene product.** **a**, Sequence logos of conserved consensus sequences identified in the alignment of MPH_02178 with 50 chimeric TSs. **b**, Summary of enzyme variants constructed in this study. **c**, Relative in vitro production of compound 2 from incubations with DMAPP and IPP (wildtype is set to 100%). Bars and error bars show mean and s.d. from three biological replicates, respectively.

**
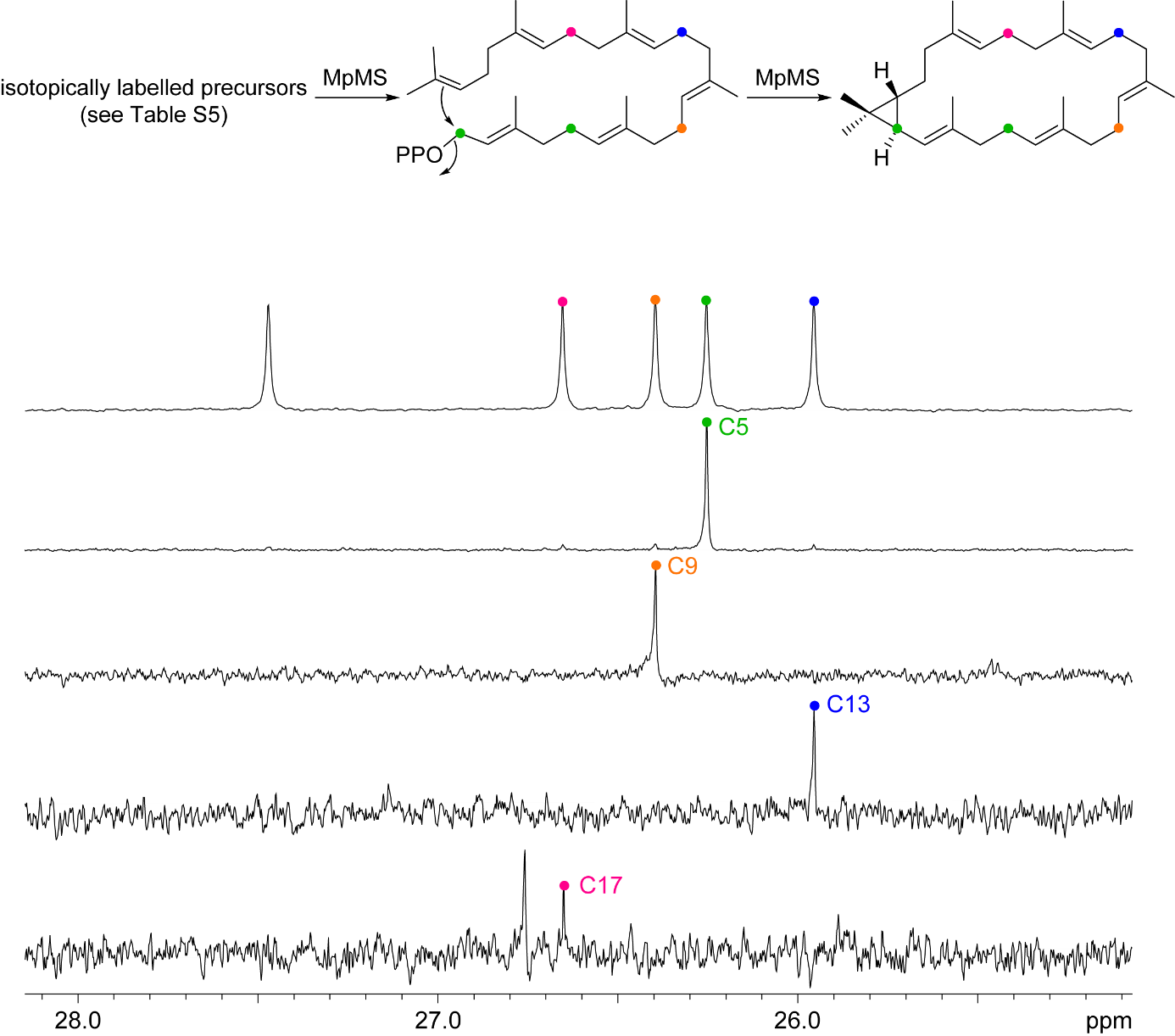
**

**Supplementary Figure 32a | Single isotopic labelling of carbons in the macrocyclic ring of 2.** Excerpts from ^13^C-NMR spectra of isotopically labelled **2**. The carbons in the macrocyclic ring which could not be clearly identified from HMBC correlations to the three-membered ring were isotopically labelled by incubating FPP, GGPP, GFPP and IPP isotopomers together with MpMS. Coloured dots indicate isotopic labelling with ^13^C.


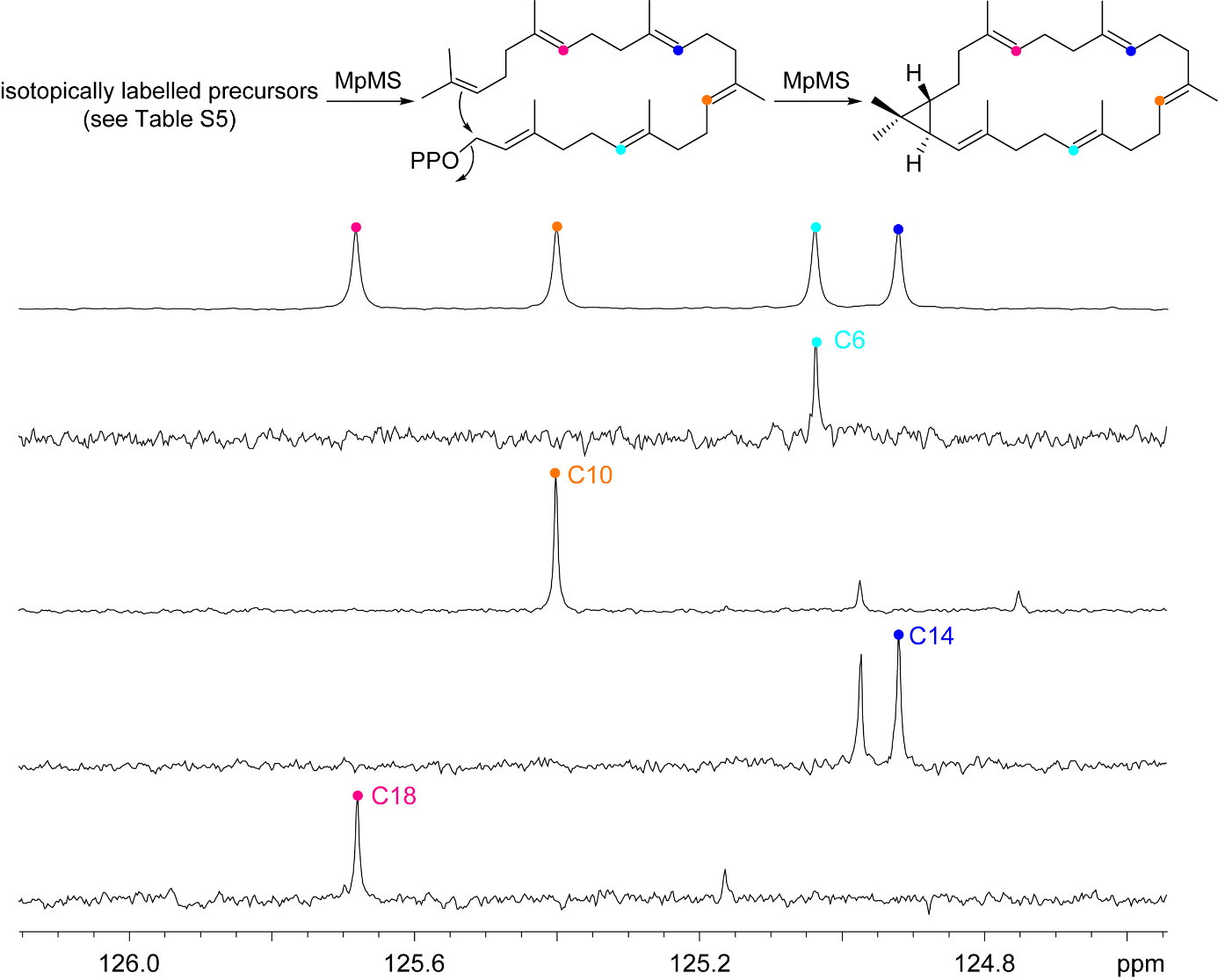


**Supplementary Figure 32b | Single isotopic labelling of carbons in the macrocyclic ring of 2.** Excerpts from ^13^C-NMR spectra of isotopically labelled **2**. The carbons in the macrocyclic ring which could not be clearly identified from HMBC correlations to the three-membered ring were isotopically labelled by incubating FPP, GGPP, GFPP and IPP isotopomers together with MpMS. Coloured dots indicate isotopic labelling with ^13^C.


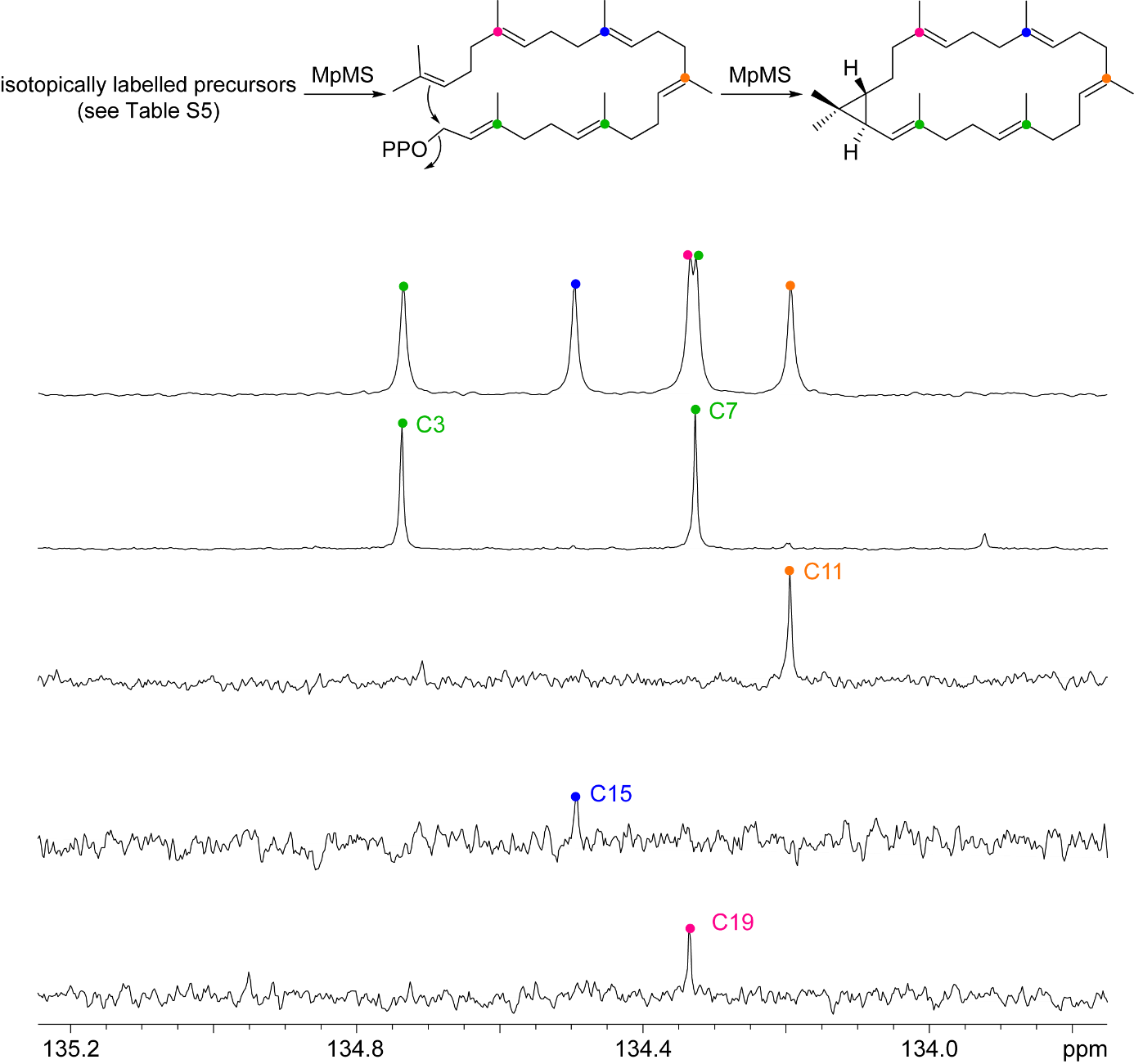


**Supplementary Figure 32c | Single isotopic labelling of carbons in the macrocyclic ring of 2.** Excerpts from ^13^C-NMR spectra of isotopically labelled **2**. The carbons in the macrocyclic ring which could not be clearly identified from HMBC correlations to the three-membered ring were isotopically labelled by incubating FPP, GGPP, GFPP and IPP isotopomers together with MpMS. Coloured dots indicate isotopic labelling with ^13^C.

**
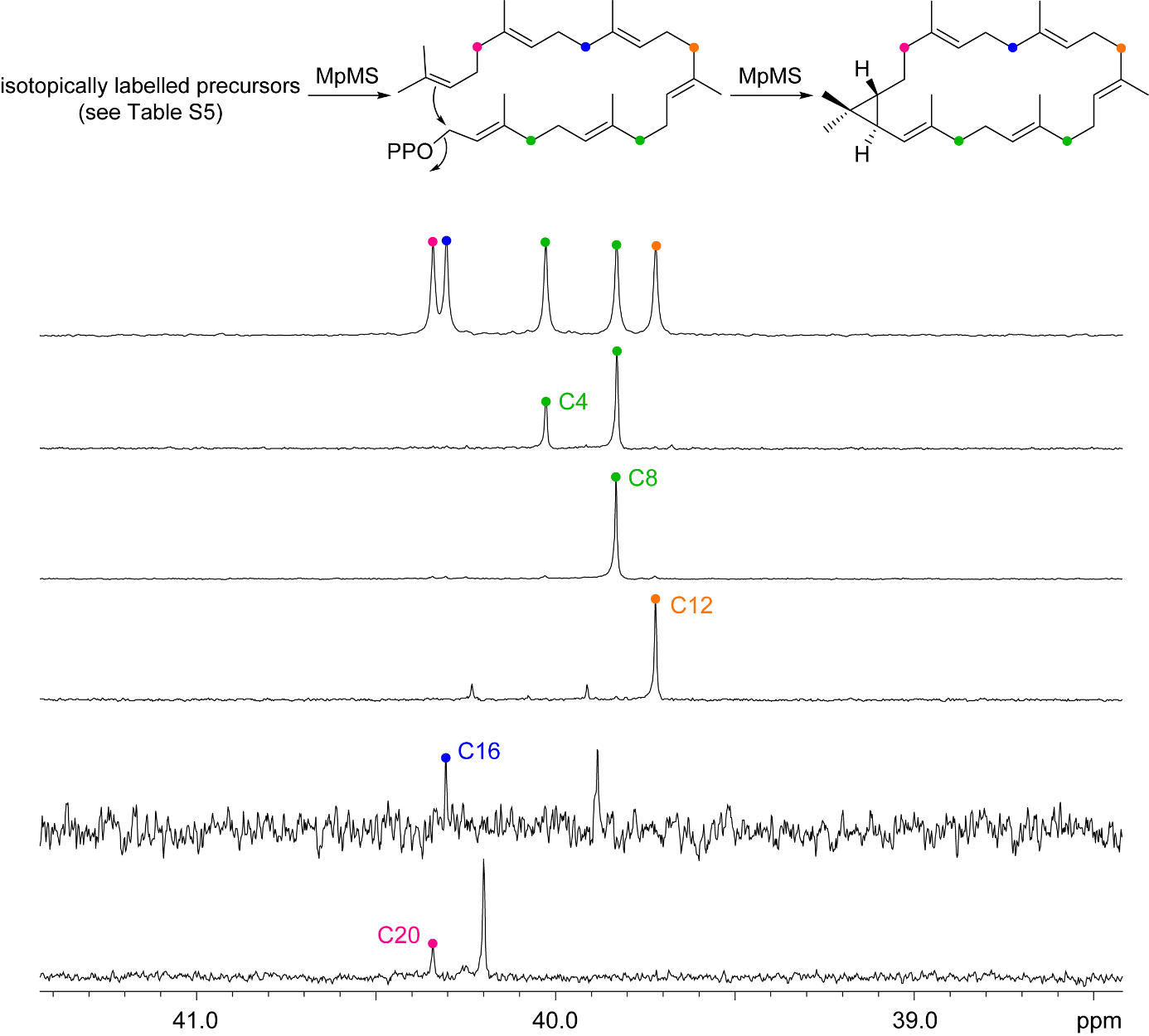
**

**Supplementary Figure 32d | Single isotopic labelling of carbons in the macrocyclic ring of 2.** Excerpts from ^13^C-NMR spectra of isotopically labelled **2**. The carbons in the macrocyclic ring which could not be clearly identified from HMBC correlations to the three-membered ring were isotopically labelled by incubating FPP, GGPP, GFPP and IPP isotopomers together with MpMS. Coloured dots indicate isotopic labelling with ^13^C.

**
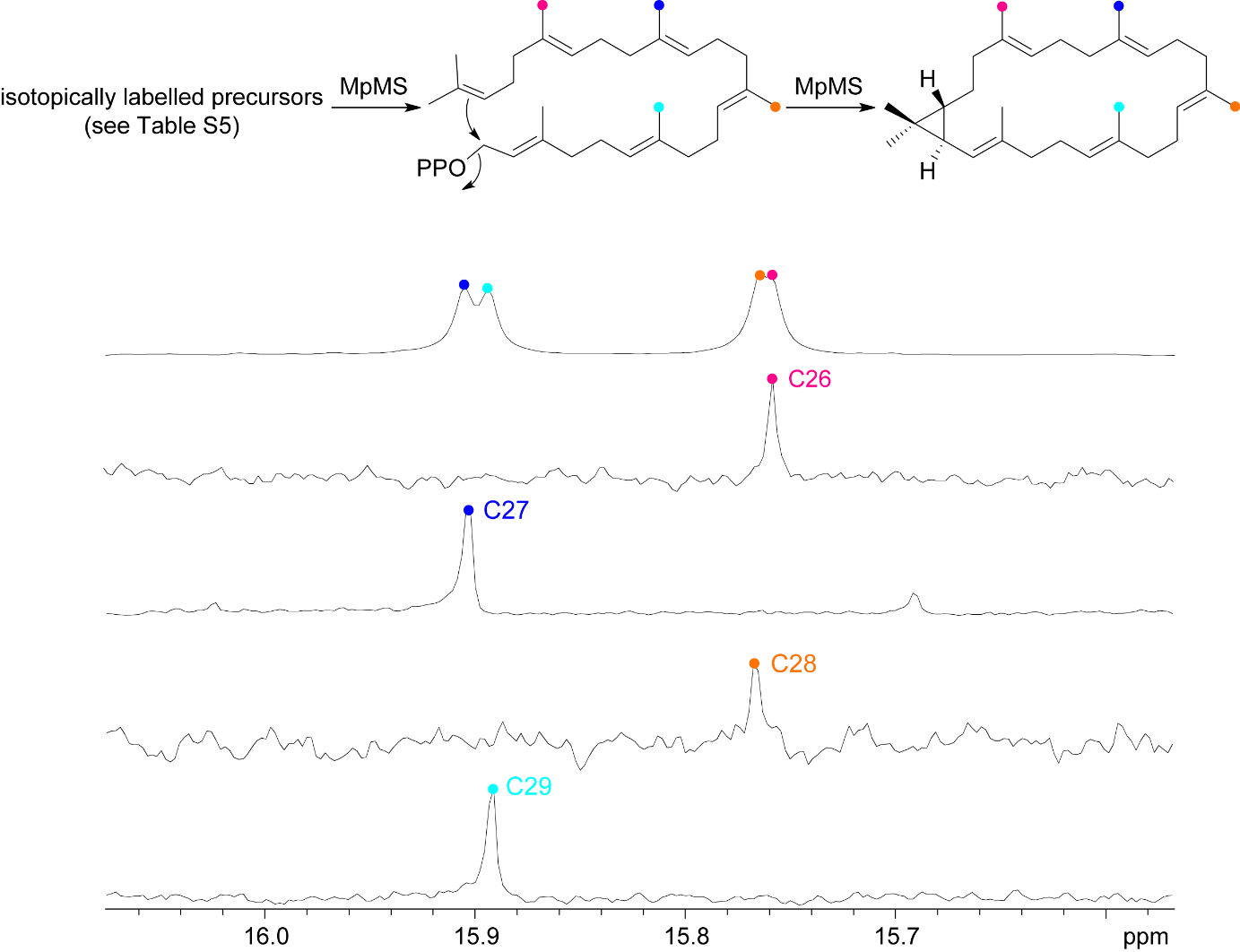
**

**Supplementary Figure 32e | Single isotopic labelling of carbons in the macrocyclic ring of 2.** Excerpts from ^13^C-NMR spectra of isotopically labelled **2**. The carbons in the macrocyclic ring which could not be clearly identified from HMBC correlations to the three-membered ring were isotopically labelled by incubating FPP, GGPP, GFPP and IPP isotopomers together with MpMS. Coloured dots indicate isotopic labelling with ^13^C.


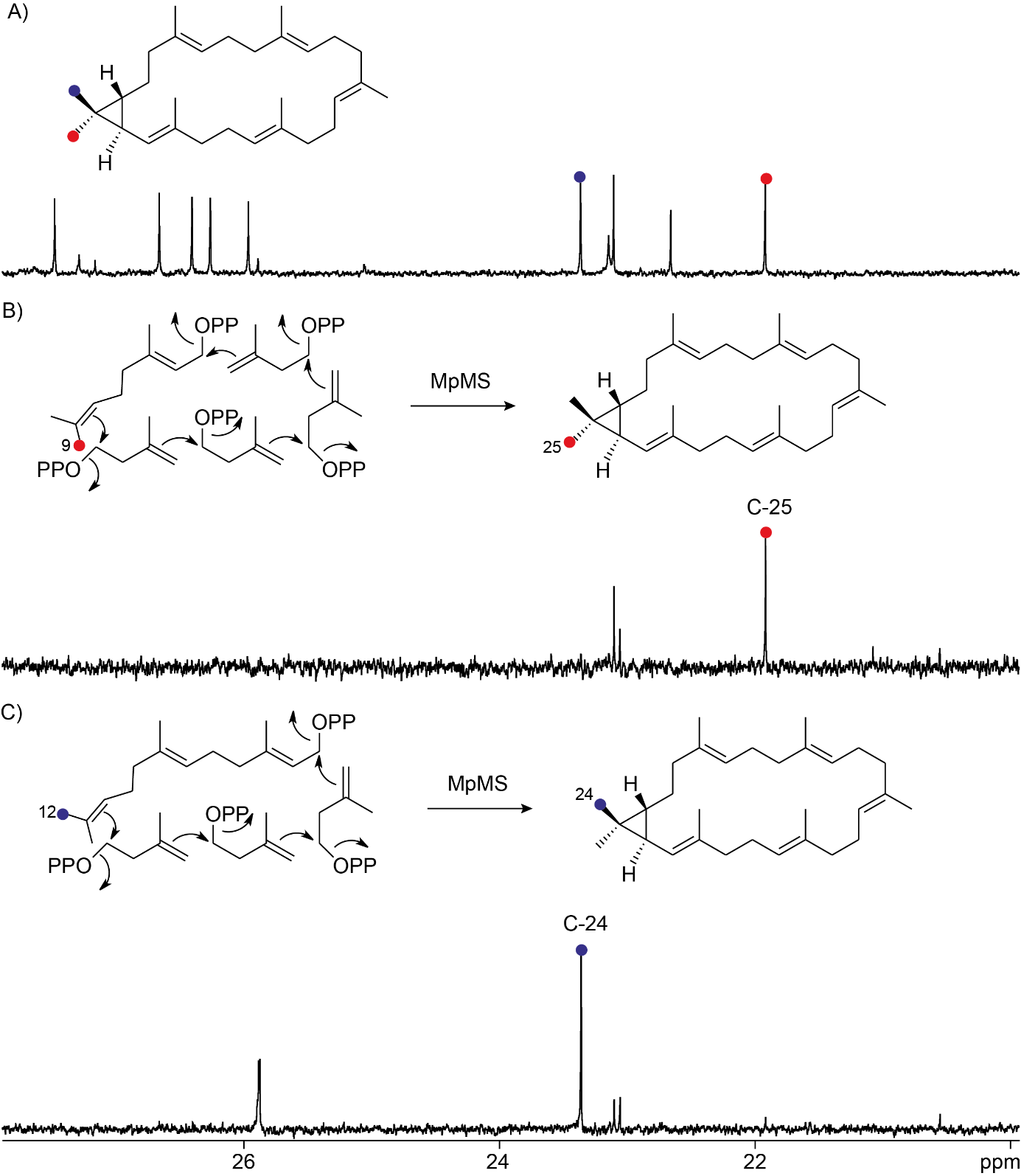


**Supplementary Figure 33 │ The stereochemical course for the geminal Me groups C-24 and C-25.** Partial ^13^C-NMR spectra of A) unlabelled macrophomene, B) the product obtained from an incubation of (9-^13^C)GPP and IPP with MpMS, and C) the product obtained from an incubation of (12-^13^C)FPP and IPP with MpMS. The appearance of only one peak corresponding to unlabelled **2** indicates a clear stereochemical course for the attack at C-23. Couloured dots indicate ^13^C-labeled carbons and corresponding peaks in the spectra.


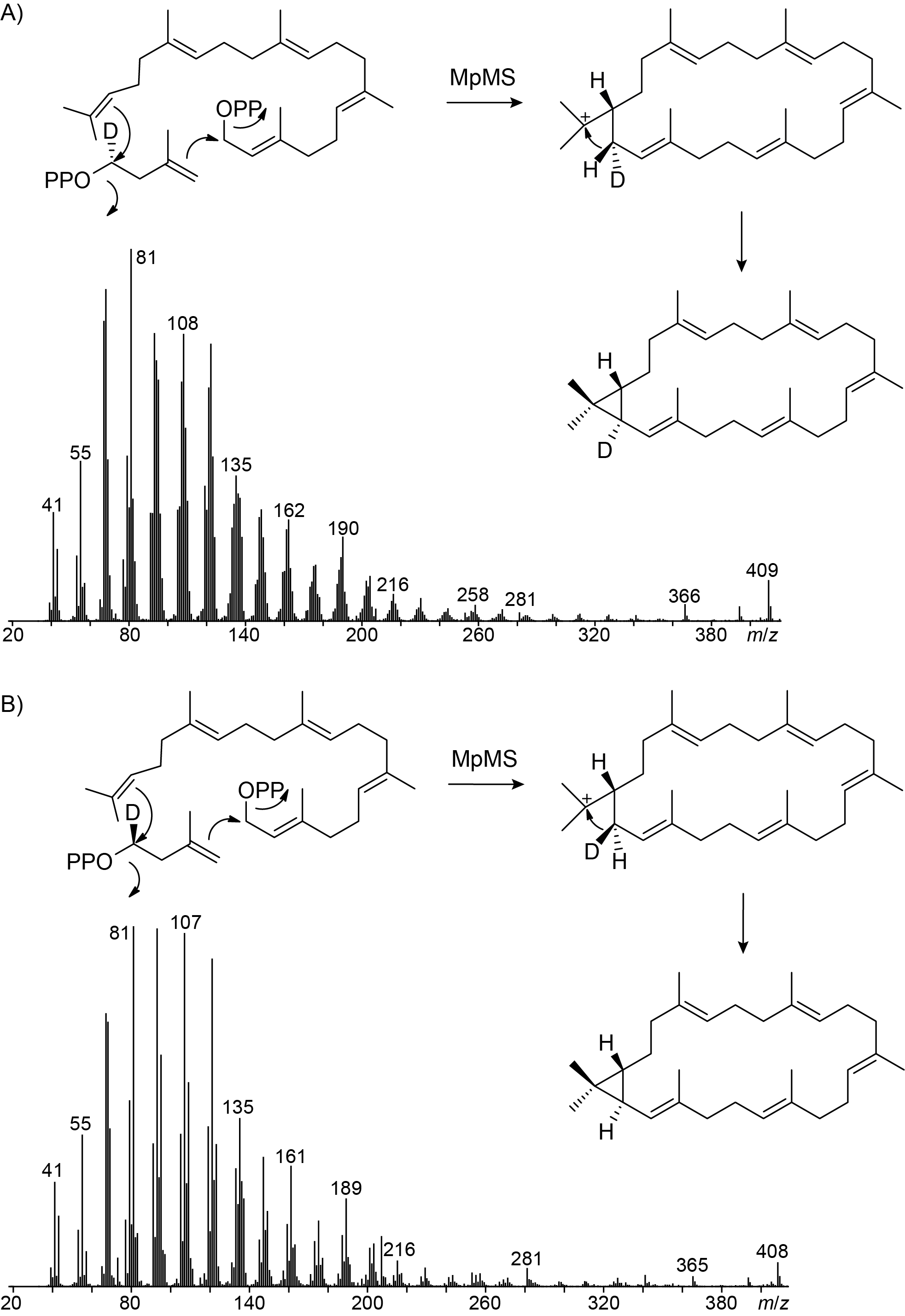


**Supplementary Figure 34 │ The stereochemical course of the deprotonation in the biosynthesis of 2.** Incubation of MpMS with GFPP and A) (*R*)- and B) (*S*)-(1-^2^H)IPP shows that deuterium is retained for the *R* enantiomer (*m*/*z* = 409), but lost for the S enantiomer (*m*/*z* 408).

**
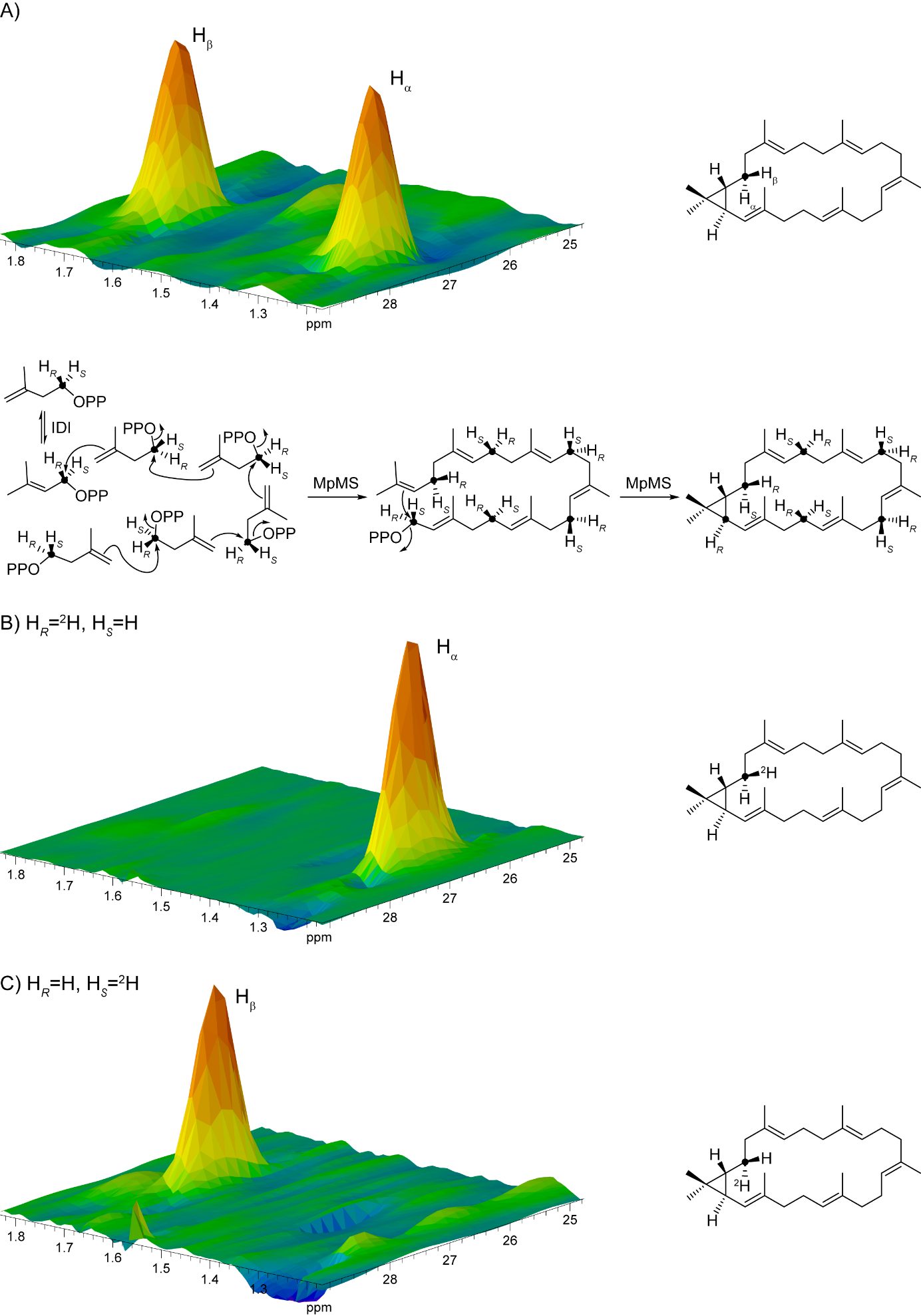
**

**Supplementary Figure 35 │ Absolute configuration of 2.** HSQC spectra of A) unlabelled **2** and from incubation of MpMS with B) with (*R*)-(1-^13^C,1-^2^H)IPP and C) (*S*)-(1-^13^C,1-^2^H)IPP, showing the absolute configuration of (1*R*,22*R*)-**2**. Black dots indicate relevant isotopically labelled positions.

**Synthesis of 2,3-dihydro-HexPP (S15)**

**Supplementary Scheme 2 │ Synthesis of 2,3-dihydro-HexPP (S15).** Reaction conditions: a) 2-iodoxybenzoic acid, DMSO, room temperature, 30 min, 83%; b) Pd(PPh_3_)_4_, Bu_3_SnH, acetic acid, benzene, room temperature, 1 h, 70%; c) NaBH_4_, MeOH/THF (1:4), 0 °C, 1 h, 93%; d) DMAP, TsCl, CH_2_Cl_2_, room temperature, overnight, 80%; e) (NBu_4_)_3_HP_2_O_7_, acetonitrile, 65%.

**Synthesis of (2*E*,6*E*,10*E*,14*E*,18*E*)-3,7,11,15,19,23-hexamethyltetracosa-2,6,10,14,18,22-hexaenal (S11)**

2-Iodoxybenzoic acid (0.27 g, 0.95 mmol, 1.4 eq) was dissolved in DMSO (4 mL), and **S8** (0.29 g, 0.68 mmol) was added dropwise. After stirring the mixture at room temperature for 30 min, the reaction mixture was diluted with Et_2_O (30 mL) and cooled to 0 °C, followed by the addition of sat. NaHCO_3_ (30 mL). The organic layer was separated, and the aqueous layer was extracted with Et_2_O (2 x 40 mL). The organic layers were combined, washed with brine, dried with MgSO_4_ and concentrated under reduced pressure. The concentrate was purified by silica gel chromatography to give compound **S11** (0.24 g, 0.57 mmol, 83%) as a colourless oil. EI-MS (70 eV): *m*/*z* (%) = 424 (1), 355 (5), 281 (7), 245 (1), 217 (2), 204 (4), 189 (5), 175 (3), 161 (8), 149 (10), 135 (20), 121 (17), 107 (18), 93 (23), 81 (52), 69 (100), 55 (15), 41 (29). GC (HP5-MS): *I* = 3172. HRMS (APCI): *m*/*z* = 425.3769 (calc. for [C_30_H_48_O + H]^+^: 425.3778). ^1^H NMR (700 MHz, C_6_D_6_): *δ* = 9.88 (d, ^3^*J*_H,H_ = 7.7 Hz, 1H), 5.84 (dq, ^3^*J*_H,H_ = 7.7 Hz, ^4^*J*_H,H_ = 1.3 Hz, 1H), 5.33 – 5.28 (m, 2H), 5.28 – 5.23 (m, 2H), 5.03 (tq, ^3^*J*_H,H_ = 7.1 Hz, ^4^*J*_H,H_ = 1.3 Hz, 1H), 2.23 – 2.09 (m, 14H), 2.06 – 2.03 (m, 2H), 1.94 – 1.90 (m, 2H), 1.82 – 1.77 (m, 2H), 1.68 (d, ^4^*J*_H,H_ = 1.3 Hz, 3H), 1.62 (s, 6H), 1.61 (d, ^4^*J*_H,H_ = 1.5 Hz, 3H), 1.57 (d, ^4^*J*_H,H_ = 1.4 Hz, 3H), 1.54 (d, ^4^*J*_H,H_ = 1.3 Hz, 3H), 1.49 (d, ^4^*J*_H,H_ = 1.3 Hz, 3H) ppm. ^13^C NMR (176 MHz, C_6_D_6_): *δ* = 189.73 (CH), 161.24 (C_q_), 136.30 (C_q_), 135.30 (C_q_), 135.13 (C_q_), 135.06 (C_q_), 131.16 (C_q_), 127.78 (CH), 124.99 (CH), 124.84 (CH), 124.81 (CH), 124.60 (CH), 123.25 (CH), 40.47 (CH_2_), 40.27 (2 x CH_2_), 40.26 (CH_2_), 40.11 (CH_2_), 27.30 (CH_2_), 27.21 (CH_2_), 27.19 (CH_2_), 27.06 (CH_2_), 25.93 (CH_3_), 25.90 (CH_2_), 17.79 (CH_3_), 16.96 (CH_3_), 16.20 (CH_3_), 16.19 (CH_3_), 16.18 (CH_3_), 16.09 (CH_3_) ppm.

**Synthesis of (6*E*,10*E*,14*E*,18*E*)-3,7,11,15,19,23-hexamethyltetracosa-6,10,14,18,22-pentaenal (S12)**

Acetic acid (34 mg, 0.57 mmol, 1.0 eq) and **S11** (0.24 g, 0.57 mmol) were added to benzene (2 mL), followed by the addition of Pd(PPh_3_)_4_ (32.9 mg, 0.03 mmol, 0.05 eq). Then Bu_3_SnH (331 mg, 1.14 mmol, 2.0 eq) was added to the mixture, and the reaction mixture was stirred at room temperature for 1 h. The reaction was quenched by pouring onto ice-water (100 mL), and the product was extracted with Et_2_O (3 x 40 mL). The combined extracts were washed with brine, dried with MgSO_4_ and concentrated under reduced pressure. The product **S12** (0.17 g, 0.40 mmol, 70%) was purified via flash chromatography (cyclohexane/ethyl acetate, 20:1, *R*_f_ = 0.42) and obtained as a colourless oil. EI-MS (70 eV): *m*/*z* (%) = 426 (3), 408 (1), 341 (2), 297 (1), 285 (1), 269 (1), 259 (3), 243 (1), 229 (3), 220 (8), 203 (11), 189 (7), 175 (6), 161 (10), 147 (17), 135 (11), 121 (23), 107 (29), 93 (29), 81 (49), 69 (100), 55 (19), 41 (33). GC (HP5-MS): *I* = 3097. HRMS (APCI): *m*/*z* = 427.3928 (calc. for [C_30_H_50_O + H]^+^: 427.3934). ^1^H NMR (700 MHz, C_6_D_6_): *δ* = 9.37 (t, ^3^*J*_H,H_ = 2.1 Hz, 1H), 5.33 – 5.28 (m, 3H), 5.24 (ddq, ^3^*J*_H,H_ = 8.4 Hz, ^3^*J*_H,H_ = 5.7 Hz, ^4^*J*_H,H_ =1.4 Hz, 1H), 5.17 (tq, ^3^*J*_H,H_ = 7.1 Hz, ^4^*J*_H,H_ = 1.4 Hz, 1H), 2.25 – 2.16 (m, 8H), 2.15 – 2.08 (m, 8H), 1.96 – 1.87 (m, 3H), 1.87 – 1.78 (m, 1H), 1.74 (ddd, ^2^*J*_H,H_ = 15.8 Hz, ^3^*J*_H,H_ = 7.8 Hz, ^3^*J*_H,H_ = 2.4 Hz, 1H), 1.68 (d, ^4^*J*_H,H_ = 1.3 Hz, 3H), 1.63 (s, 3H), 1.62 (s, 3H), 1.61 (d, ^4^*J*_H,H_ = 1.3 Hz, 3H), 1.58 (d, ^4^*J*_H,H_ = 1.3 Hz, 3H), 1.57 (d, ^4^*J*_H,H_ = 1.3 Hz, 3H), 1.23 – 1.16 (m, 1H), 1.10 – 1.04 (m, 1H), 0.75 (d, ^3^*J*_H,H_ = 6.6 Hz, 3H) ppm. ^13^C NMR (176 MHz, C_6_D_6_): *δ* = 200.77 (CH), 135.27 (C_q_), 135.16 (C_q_), 135.09 (C_q_), 135.04 (C_q_), 131.15 (C_q_), 124.99 (CH), 124.86 (CH), 124.85 (CH), 124.76 (CH), 124.68 (CH), 50.99 (CH_2_), 40.28 (CH_2_), 40.27 (CH_2_), 40.26 (CH_2_), 40.23 (CH_2_), 37.17 (CH_2_), 27.81 (CH), 27.30 (CH_2_), 27.21 (CH_2_), 27.19 (CH_2_), 27.13 (CH_2_), 25.90 (CH_3_), 25.71 (CH_2_), 19.86 (CH_3_), 17.79 (CH_3_), 16.21 (CH_3_), 16.20 (CH_3_), 16.18 (CH_3_), 16.12 (CH_3_) ppm.

**Synthesis of (6*E*,10*E*,14*E*,18*E*)-3,7,11,15,19,23-hexamethyltetracosa-6,10,14,18,22-pentaen-1-ol (S13)**

Aldehyde **S12** (170 mg, 0.40 mmol) was added to methanol/THF (1:4, 3 mL). The mixture was cooled to 0 °C, followed by the addition of NaBH_4_ (21 mg, 0.56 mmol, 1.4 eq). After stirring at 0 °C for 1 h, the reaction was quenched by adding sat. NH_4_Cl (40 mL). The product was extracted with Et_2_O (3 x 40 mL). The extracts were dried with MgSO_4_ and concentrated under reduced pressure. The product (160 mg, 0.37 mmol, 93%) was purified by column chromatography (cycolhexane/ethyl acetate, 6:1, *R*_f_ = 0.31) and obtained as a colourless oil. EI-MS (70 eV): *m*/*z* (%) = 428 (1), 385 (1), 359 (3), 291 (1), 247 (2), 221 (1), 208 (3), 159 (5), 175 (3), 161 (8), 149 (9), 135 (20), 121 (18), 109 (15), 95 (28), 81 (63), 69 (100), 55 (15), 41 (27). GC (HP5-MS): *I* = 3096. HRMS (APCI): *m*/*z* = 429.4084 (calc. for [C_30_H_52_O + H]^+^: 429.4091). ^1^H NMR (700 MHz, C_6_D_6_): *δ* = 5.34 – 5.29 (m, 3H), 5.28 – 5.23 (m, 2H), 3.46 – 3.36 (m, 2H), 2.25 – 2.16 (m, 8H), 2.11 (p, *J* = 7.2 Hz, 8H), 2.08 – 1.98 (m, 2H), 1.68 (q, ^4^*J*_H,H_ = 1.3 Hz, 3H), 1.63 – 1.61 (m, 12H), 1.57 (d, ^4^*J*_H,H_ = 1.4 Hz, 3H), 1.56 – 1.52 (m, 1H), 1.49 – 1.43 (m, 1H), 1.39 – 1.32 (m, 1H), 1.22 – 1.17 (m, 2H), 0.84 (d, ^3^*J*_H,H_ = 6.7 Hz, 3H), 0.48 (t, ^3^*J*_H,H_ = 5.2 Hz, 1H) ppm. ^13^C NMR (176 MHz, C_6_D_6_): *δ* = 135.07 (C_q_), 135.06 (C_q_), 135.03 (C_q_), 134.85 (C_q_), 131.15 (C_q_), 125.33 (CH), 125.00 (CH), 124.89 (CH), 124.87 (CH), 124.86 (CH), 60.89 (CH_2_), 40.29 (2 x CH_2_), 40.28 (CH_2_), 40.27 (CH_2_), 40.26 (CH_2_), 37.69 (CH_2_), 29.55 (CH), 27.30 (CH_2_), 27.22 (CH_2_), 27.19 (CH_2_), 27.17 (CH_2_), 25.90 (CH_3_), 25.90 (CH_2_), 19.77 (CH_3_), 17.79 (CH_3_), 16.21 (CH_3_), 16.19 (CH_3_), 16.17 (CH_3_), 16.15 (CH_3_) ppm.

**Synthesis of (6*E*,10*E*,14*E*,18*E*)-3,7,11,15,19,23-hexamethyltetracosa-6,10,14,18,22-pentaen-1-yl 4-methylbenzenesulfonate (S14)**

Alcohol **S13** (108 mg, 0.25 mmol) and DMAP (100 mg, 0.82 mmol, 3.3 eq) were added into CH_2_Cl_2_ (4 mL). The mixture was cooled to 0 °C, followed by the addition of TsCl (120 mg, 0.63 mmol, 2.5 eq, in 4 mL CH_2_Cl_2_). The reaction was stirred overnight at room temperature and quenched by pouring into ice-cold NH_4_Cl solution (60 mL sat. NH_4_Cl with 100 mL ice-water). The product was extracted with Et_2_O (3 x 60 mL). The combined organic layers were dried with MgSO_4_ and concentrated under reduced pressure. The product **S14** (119 mg, 0.20 mmol, 80%) was purified via silica gel chromatography (cyclohexane/ethyl acetate, 10:1, *R*_f_ = 0.47) and obtained as a colourless oil. HRMS (APCI): *m*/*z* = 583.4171 (calc. for [C_37_H_58_O_3_S + H]^+^: 583.4179). ^1^H NMR (500 MHz, C_6_D_6_): *δ* = 7.82 – 7.75 (m, 2H), 6.75 – 6.68 (m, 2H), 5.34 – 5.28 (m, 3H), 5.25 (ddp, ^3^*J*_H,H_ = 8.5 Hz, ^3^*J*_H,H_ = 5.8 Hz, ^4^*J*_H,H_ = 1.4 Hz, 1H), 5.15 (tq, ^3^*J*_H,H_ = 7.0 Hz, ^4^*J*_H,H_ = 1.3 Hz, 1H), 4.02 – 3.87 (m, 2H), 2.25 – 2.15 (m, 8H), 2.15 – 2.06 (m, 8H), 1.97 – 1.79 (m, 2H), 1.84 (s, 3H), 1.68 (d, ^4^*J*_H,H_ = 1.2 Hz, 3H), 1.63 (d, ^4^*J*_H,H_ = 1.3 Hz, 3H), 1.63 – 1.61 (m, 6H), 1.57 (s, 6H), 1.50 – 1.35 (m, 2H), 1.18 – 1.10 (m, 2H), 1.03 – 0.94 (m, 1H), 0.64 (d, ^3^*J*_H,H_ = 6.6 Hz, 3H) ppm. ^13^C NMR (126 MHz, C_6_D_6_): *δ* = 144.05 (C_q_), 135.14 (C_q_), 135.08 (2 x C_q_), 135.04 (C_q_), 134.73 (C_q_), 131.15 (C_q_), 129.79 (2 x CH), 128.20 (2 x CH), 124.99 (CH), 124.88 (CH), 124.86 (2 x CH), 124.81 (CH), 68.68 (CH_2_), 40.29 (CH_2_), 40.27 (3 x CH_2_), 37.05 (CH_2_), 35.93 (CH_2_), 29.19 (CH), 27.30 (CH_2_), 27.22 (CH_2_), 27.19 (2 x CH_2_), 25.90 (CH_3_), 25.61 (CH_2_), 21.15 (CH_3_), 19.09 (CH_3_), 17.80 (CH_3_), 16.22 (CH_3_), 16.20 (CH_3_), 16.18 (CH_3_), 16.13 (CH_3_) ppm.

**Synthesis of trisammonium 2,3-dihydroHexPP (S15)**

To an acetonitrile (0.3 mL) solution of (NBu_4_)_3_HP_2_O_7_ (69 mg, 0.08 mmol, 1.5 eq) was added **S14** (30 mg, 0.05 mmol, in 0.8 mL acetonitrile) dropwise. The mixture was stirred overnight at room temperature, and the solvent was removed under reduced pressure. The product **S15** (21 mg, 0.03 mmol, 65%) was purified via silica gel chromatography (*i*PrOH/25% NH_4_OH/H_2_O, 6:2.5:0.5, *R*_f_ = 0.1) and obtained as colourless powder. HRMS (ESI): *m*/*z* = 587.3266 (calc. for [C_30_H_53_O_7_P_2_]^–^: 587.3272). ^1^H NMR (700 MHz, D_2_O): *δ* = 5.33 – 5.14 (m, 5H), 4.09 (d, ^3^*J*_H,P_ = 39.1 Hz, 2H), 2.23 – 2.01 (m, 18H), 1.87 – 1.66 (m, 2H), 1.77 (s, 3H), 1.73 (s, 3H), 1.71 (s, 3H), 1.70 (s, 3H), 1.69 (s, 6H), 1.57 – 1.44 (m, 2H), 1.33 – 1.27 (m, 1H), 1.07 (d, ^3^*J*_H,H_ = 6.5 Hz, 3H) ppm. ^13^C NMR (176 MHz, D_2_O): *δ* = 134.31 (C_q_), 134.25 (2 x C_q_), 134.03 (C_q_), 130.47 (C_q_), 125.16 (CH), 124.40 (CH), 124.36 (CH), 124.31 (CH), 124.23 (CH), 64.40 (d, ^2^*J*_C,P_ = 5.3 Hz, CH_2_), 39.95 (CH_2_), 39.78 (CH_2_), 39.71 (2 x CH_2_), 37.95 (CH_2_), 37.21 (CH_2_), 29.54 (CH), 27.00 (CH_2_), 26.77 (CH_2_), 26.73 (CH_2_), 26.61 (CH_2_), 25.50 (CH_2_), 25.46 (CH_3_), 18.76 (CH_3_), 17.38 (CH_3_), 15.80 (CH_3_), 15.76 (2 x CH_3_), 15.74 (CH_3_) ppm. ^31^P NMR (162 MHz, D_2_O): *δ* = – 10.21 (m), –10.68 (d, ^2^*J*_P,P_ = 19.4 Hz) ppm.


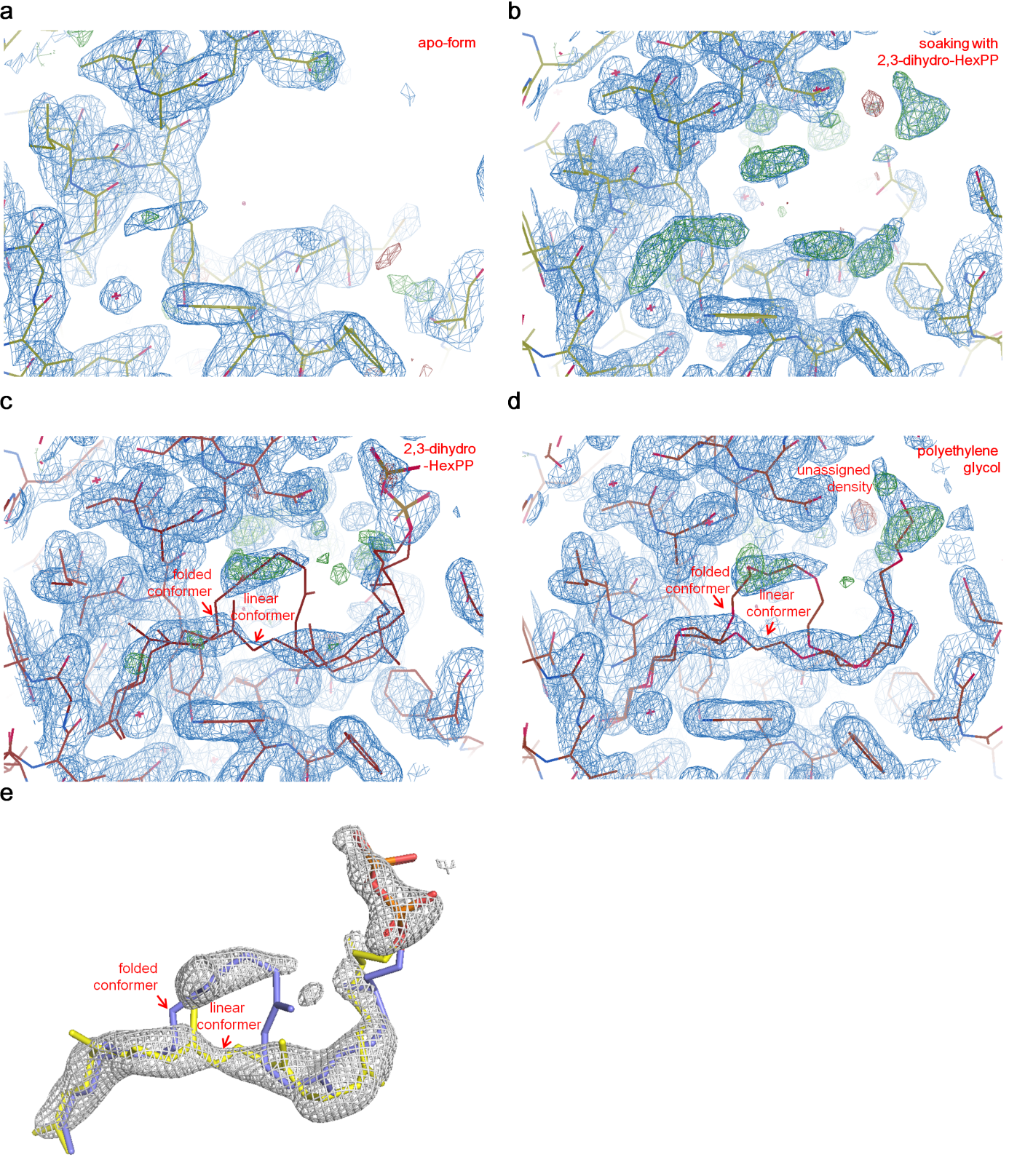


**Supplementary Figure 36 │** **Additional electron density observed after soaking of TvTS with the non-reactive substrate surrogate 2,3-dihydro-HexPP.** Electron density map of **a**, TvTS (apo) and **b**, TvTS after soaking with 2,3-dihydro-HexPP. The additional electron density observed close to the DDXXD motif is likely from 2,3-dihydro-HexPP, but could not be assigned with certainty. The 2mFo-DFc (blue mesh) and the mFo-DFc maps (positive: green mesh, negative: red mesh) are represented and contoured at 1.2 and 3.5 σ, respectively. **c**, Fitting of 2,3-dihydro-HexPP reveals that some of isoprenoid methyl groups stick out, while for **d**, fitting of polyethylene glycol (PEG) as used in the crystallization buffer density close to DDXXD motif remained unassigned. The 2mFo-DFc and mFo-DFc maps are represented and contoured at 0.8 and 3.5 σ, respectively. Since in **c** and **d** both possible ligands did not fit perfectly, no modelled ligand was added to the deposited structure (7VTB). **e**, For further structure based experimental work, docking of 2,3-dihydro-HexPP in two possible conformations to the TvTS active site was performed. The Fo-Fc polder omit map of ligands are represented as a gray mesh, contoured at +3.0 σ.


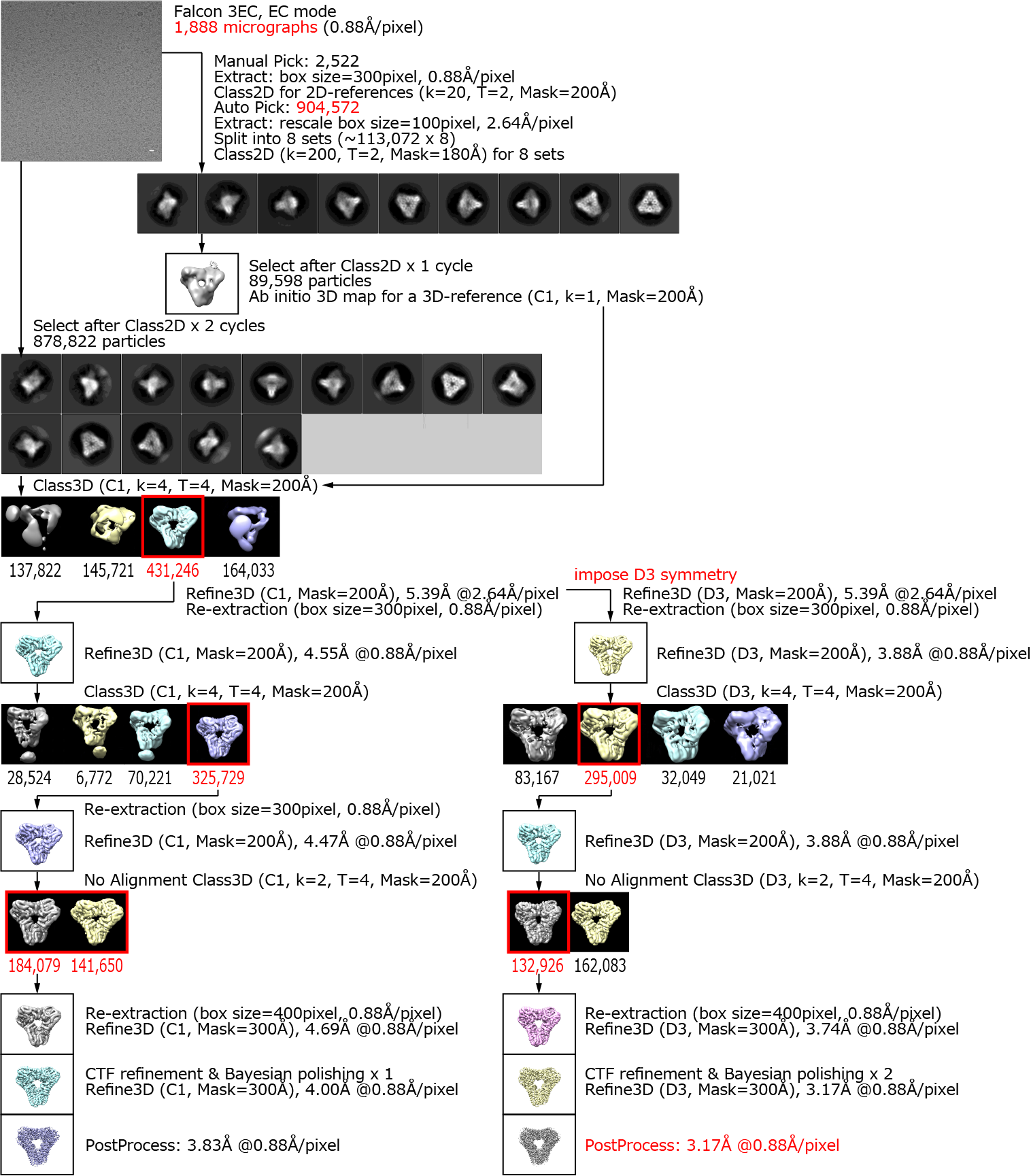


**Supplementary Figure 37 │** **Workflow for cryo-EM data-processing of MpMS.** Data processing workflow used to obtain the final cryo-EM map of MpMS.


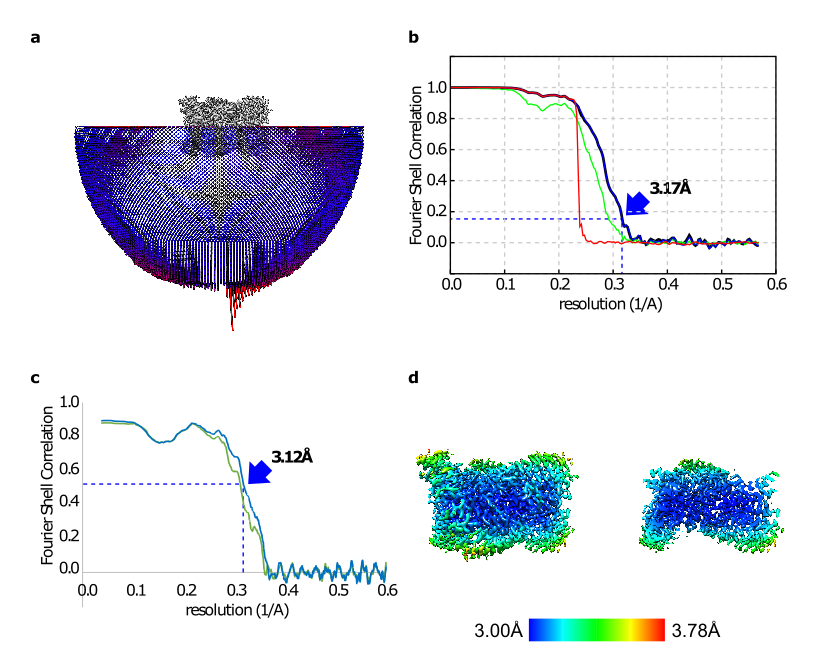


**Supplementary Figure 38 │** **Cryo-EM data-processing of MpMS. a**, Orientation distribution, **b**, half- sets FSC curves, **c**, map-to-model FSC curve, and **d**, outer (left) and inner (right) local resolutions of the cryo-EM maps of MpMS. In the half-sets FSC curves, FSC corrected (black), FSC unmasked (green), FSC masked (blue), and corrected FSC phase randomized (red) maps are shown.


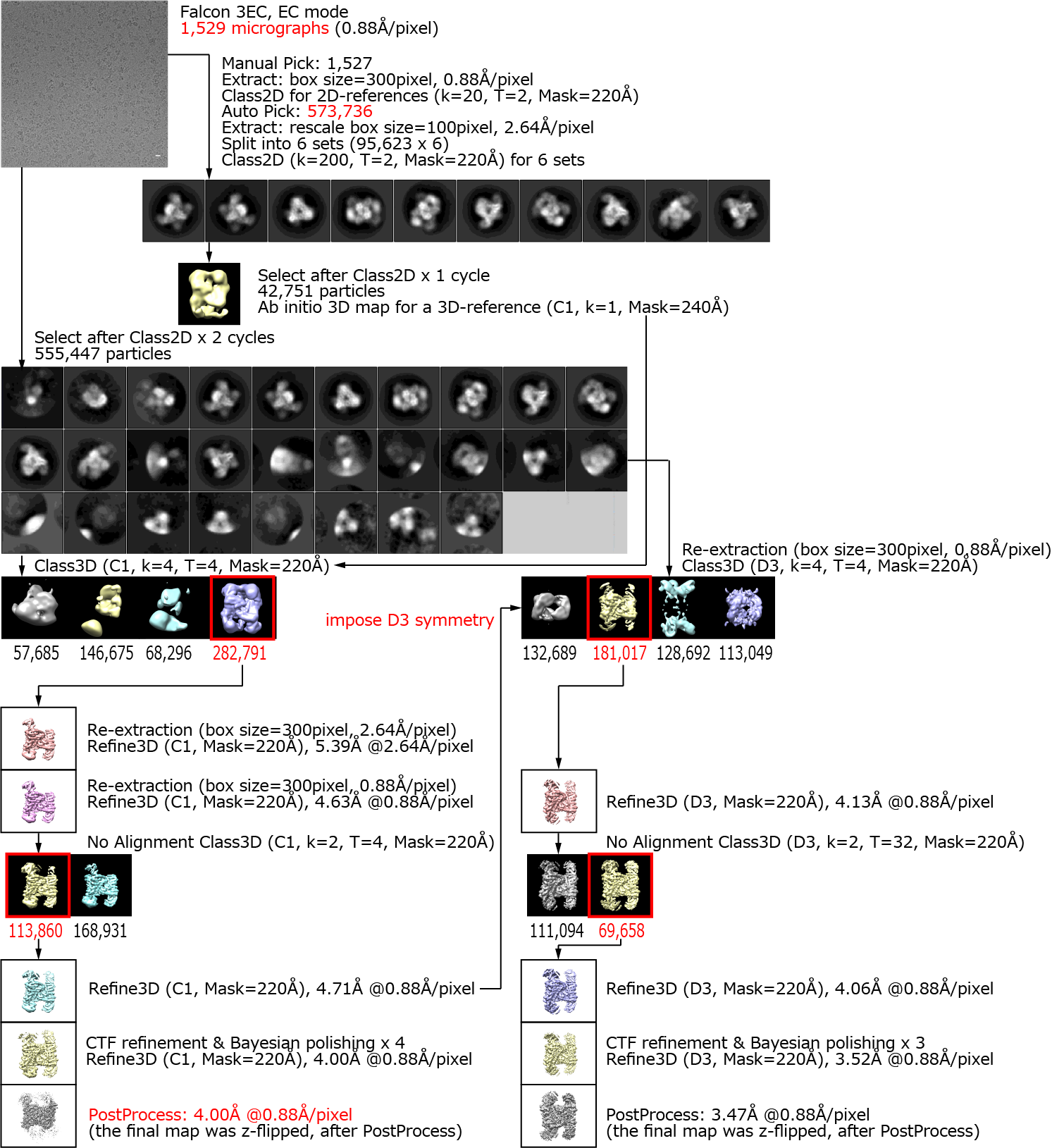


**Supplementary Figure 39 │** **Workflow for cryo-EM data-processing of crosslinked MpMS.** Data processing workflow used to obtain the final cryo-EM map of crosslinked MpMS.


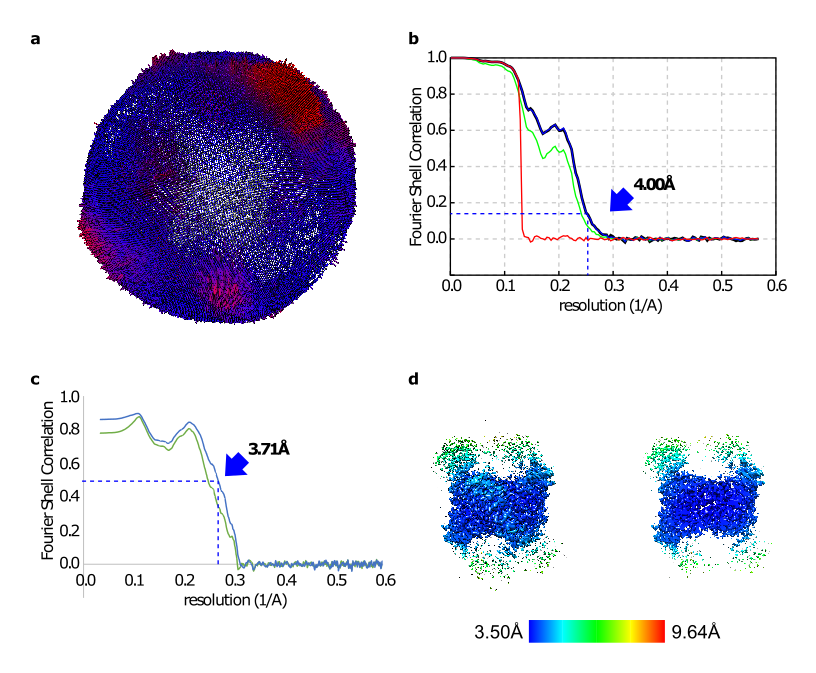


**Supplementary Figure 40 │** **Cryo-EM data-processing of crosslinked MpMS. a**, Orientation distribution, **b**, half- sets FSC curves, **c**, map-to-model FSC curve, and **d**, outer (left) and inner (right) local resolutions of the cryo-EM maps of crosslinked MpMS. In the half-sets FSC curves, FSC corrected (black), FSC unmasked (green), FSC masked (blue), and corrected FSC phase randomized (red) maps are shown.


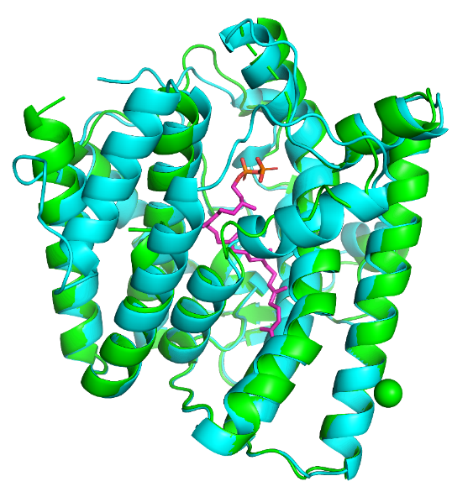


**Supplementary Figure 41 │ The alignment of crystal and predicted structure of TvTS-TC.** Crystal structure (green) and the AlphaFold2 predicted structure (blue) of TvTS-TC shows high consistency with each other with 0.59 Å Root Mean Square Deviation (RMSD).


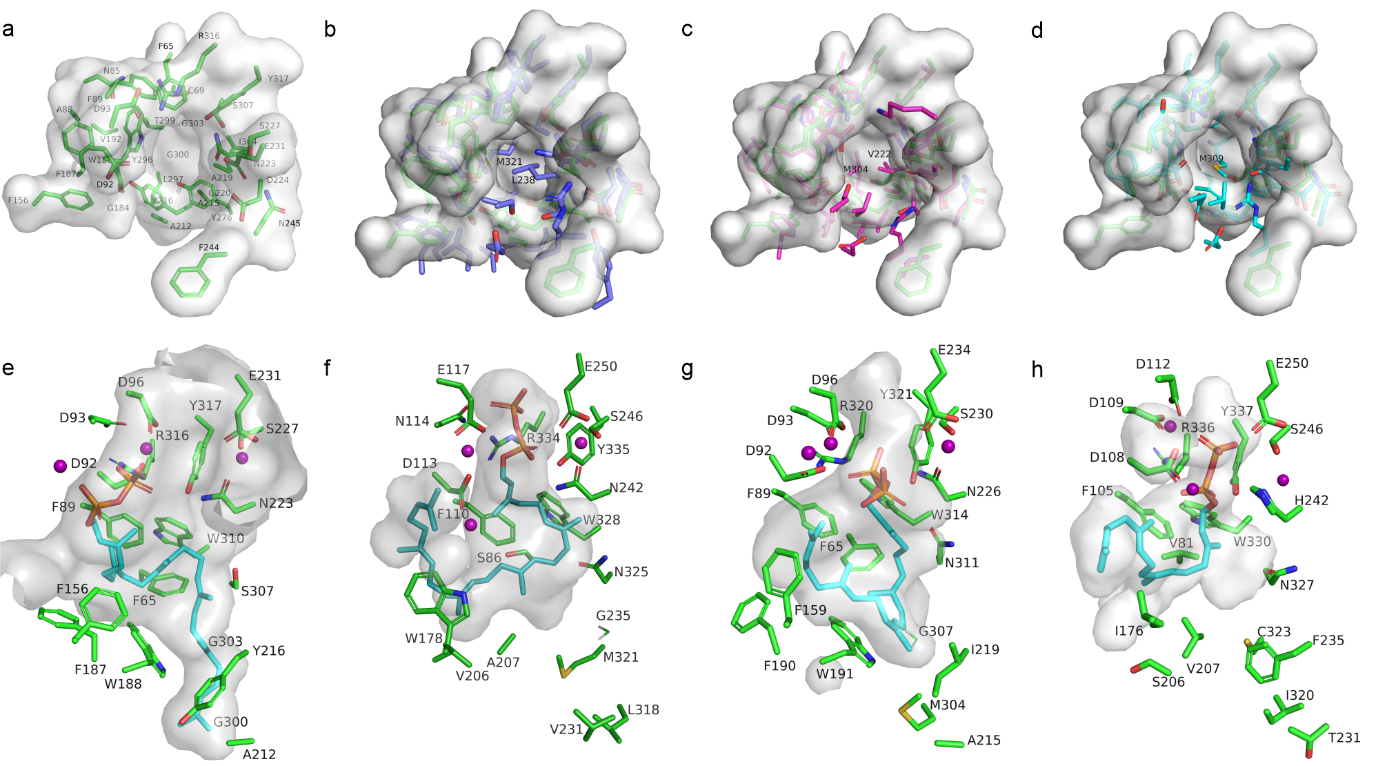


**Supplementary Figure 42 │ AlphaFold2-based structure prediction and the substrate docking.** **a**) The surface of residues around the binding pocket of TvTS-TC (green sticks), **b**) the alignment of binding pockets between TvTS-TC and MpMS-TC (blue sticks), **c**) the alignment of binding pockets between TvTS-TC and FgMS-TC (magenta sticks), **d**) the alignment of binding pockets between TvTS-TC and CgDS-TC (cyan sticks), the residues extend out the TvTS-TC binding pocket surface were labled. **e**) TvTS-TC docking with HexPP, **f**) MpMS-TC docking with HexPP, **g**) FgMS-TC docking with GFPP, and **h**) CgDS-TC docking with GGPP. The green sticks in **e-h** represent residues of receptors, cyan sticks represent ligands and purple balls represent Mg^2+^.


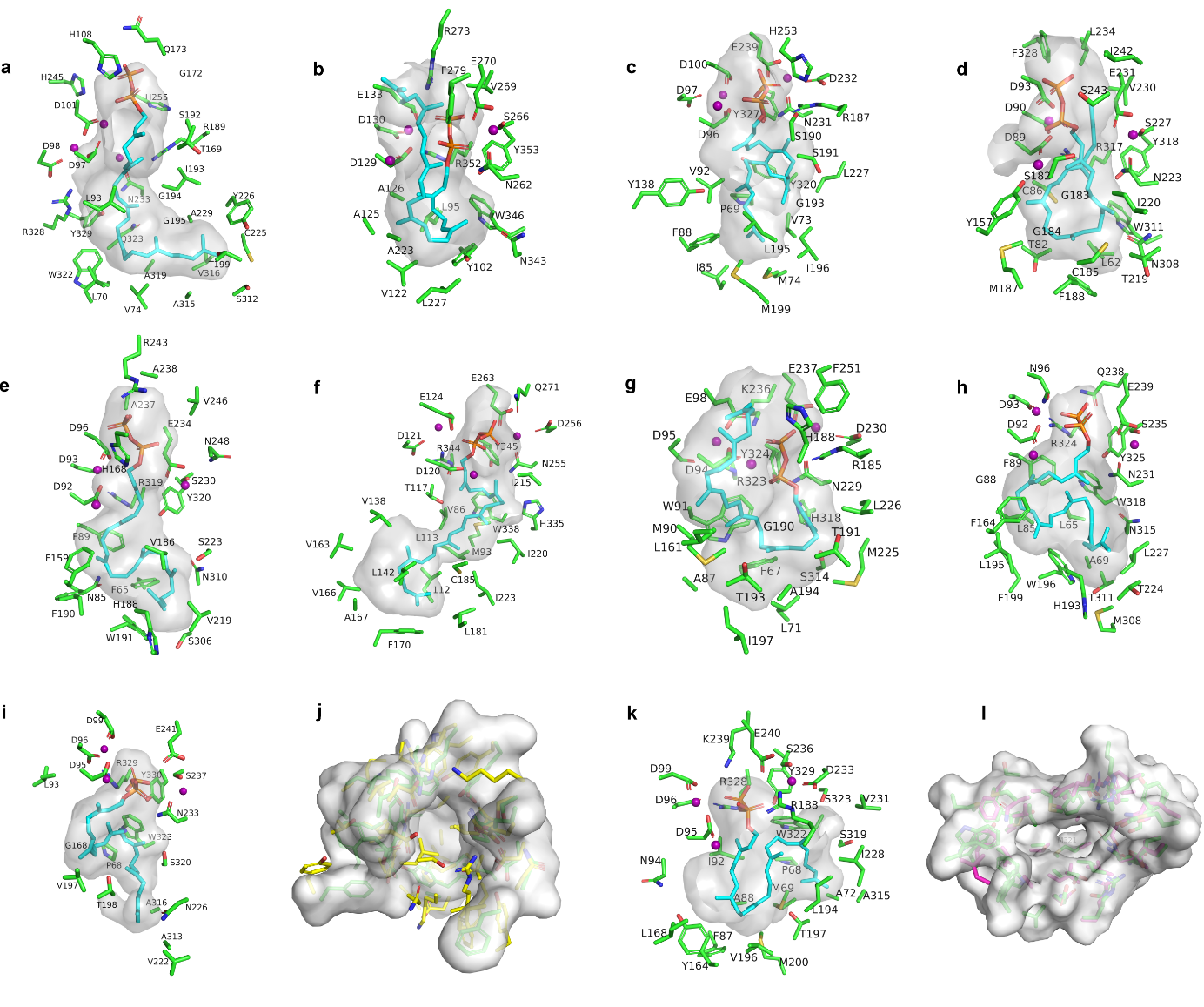


**Supplementary Figure 43 │ AlphaFold2-based structure prediction and the substrate docking of class I triterpene candidates.** AlphFold2 predicted substrate binding pocket docking with HexPP of **a**) PTTC027-TC, **b**) PTTC035-TC, **c**) PTTC044-TC, **d**) PTTC058-TC, **e**) PTTC059-TC, **f**) PTTC060-TC, **g**) PTTC062-TC, **h**) PTTC114-TC, **i**) Cgl13855-TC and **k**) PTTC074-TC. **j**) the alignment of binding pockets between TvTS-TC (green sticks, showed as surface) and Cgl13855-TC (yellow sticks), **l**) the alignment of binding pockets between MpMS (green sticks, showed as surface) and PTTC074-TC (magenta sticks). Green sticks in **a**-**i** and **k** represent residues of receptors, cyan sticks represent ligands and purple balls represent Mg^2+^.

**Supplementary Table 7 │ The docking results between predicted TC domains and ligands.**

| TC domain | ligand | Volume^a^ (Å^3^) | Aera^b^ (Å^2^) | Depth^c^ (Å) | Docking score |
| --- | --- | --- | --- | --- | --- |
| TvTS | HexPP | 584.6 | 528.1 | 23.5 | -10.6 |
| MpMS | HexPP | 573.1 | 566.6 | 16.3 | -6.0 |
| FgMS | GFPP | 510.6 | 417.5 | 17.3 | -8.8 |
| CgDS | GGPP | 422.1 | 411.7 | 15.8 | -7.4 |
| Cgl13855 | HexPP | 589.5 | 549.1 | 23.3 | -8.6 |
| PTTC027 | HexPP | 577.4 | 550.6 | 20.3 | -9.8 |
| PTTC035 | HexPP | 589.6 | 535.8 | 14.1 | -9.6 |
| PTTC044 | HexPP | 591.7 | 507.6 | 17.8 | -9.0 |
| PTTC058 | HexPP | 585.2 | 509.3 | 14.2 | -8.2 |
| PTTC059 | HexPP | 583.3 | 508.9 | 18.0 | -9.8 |
| PTTC060 | HexPP | 585.7 | 538.6 | 21.4 | -8.4 |
| PTTC062 | HexPP | 595.9 | 530.4 | 14.8 | -9.0 |
| PTTC074 | HexPP | 597.1 | 501.4 | 19.4 | -8.0 |
| PTTC114 | HexPP | 595.1 | 526.9 | 18.1 | -9.9 |

^a^ The enclosed volume of ligand surface in the docking complex; ^b^ The surface area of ligand surface in the docking complex; c The distance between β carbon atom of the third Asp in DDXXD domain and the carbon atom of ligand at the bottom of binding pocket.


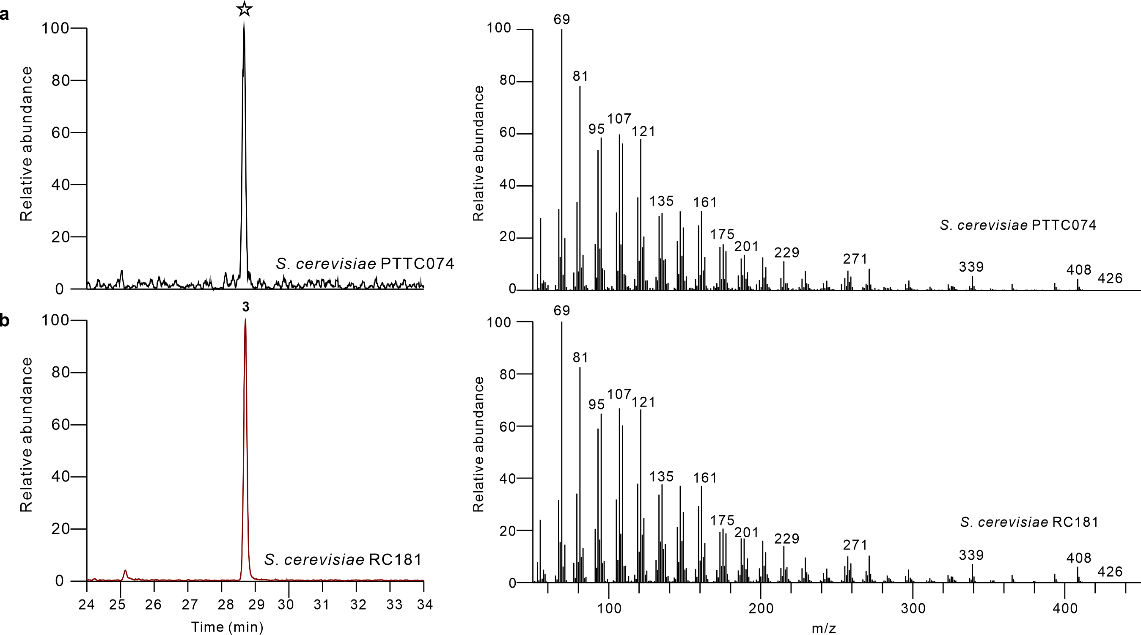


**Supplementary Figure 44 │ GCMS detection of 3 produced by a) *S. cerevisiae* PTTC074 and b) *S. cerevisiae* RC181.** *S. cerevisiae* RC181 harboring the Cgl13855 (CgCS) from *C. gloeosporioides* ES026. White asterisks indicate the peak produced by PTTC074 with same retention time and mass fragment as compound 3 (colleterpenol) that produced by CgCS.

**Supplementary Figure 45 │ Structure elucidation of 3.** Carbon numbering follows HexPP numbering to indicate the biosynthetic origin of each carbon by same number.


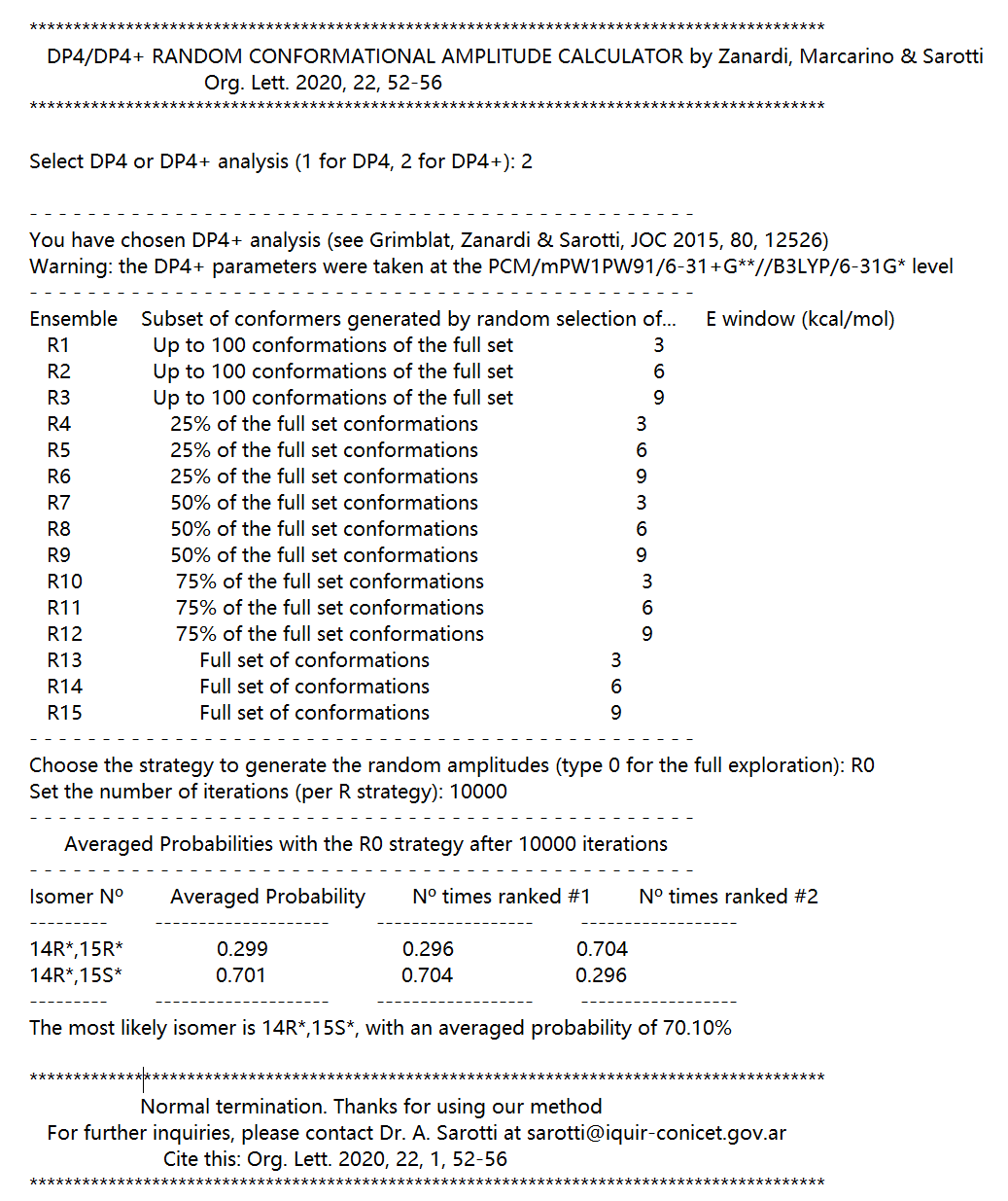


**Supplementary Figure 46 │ DP4+ analysis of (14*R**,15*S**)-3 and (14*R**,15*R**)-3 based on random conformational amplitudes.**


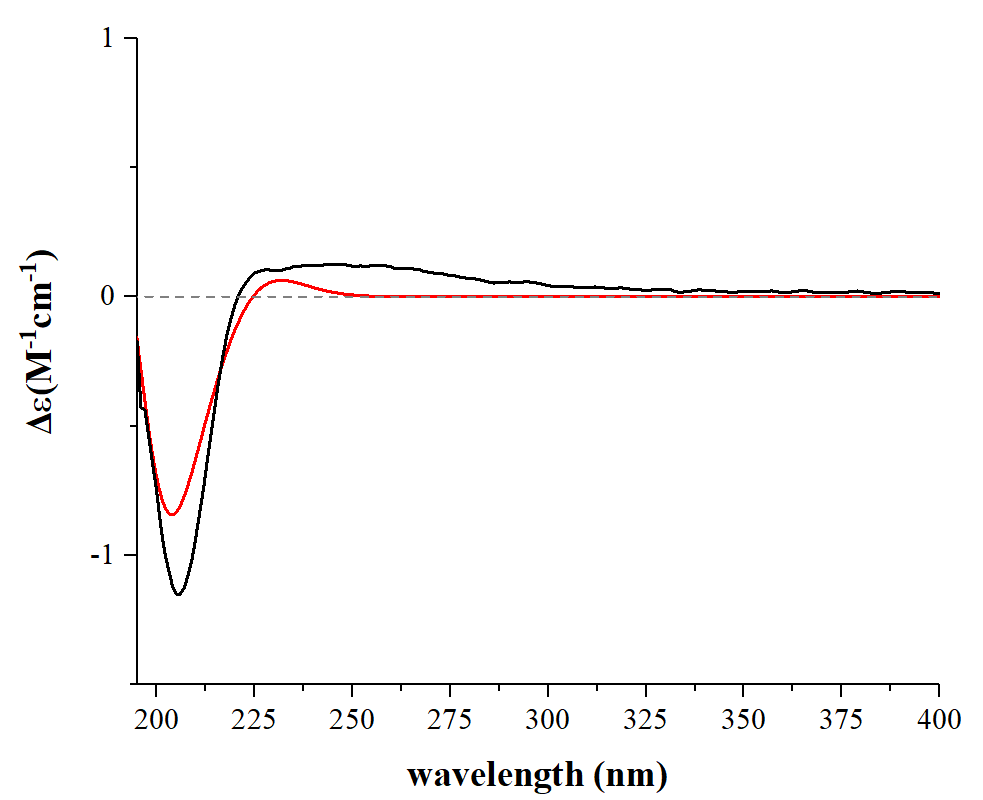


**Supplementary Figure 47 │ The experimental ECD spectrum of 3 (black curve), and the calculated ECD spectrum of (14*R*,15*S*)-3 (red curve).**

**Supplementary Table 8 │ NMR data of 3 recorded in CDCl_3_ (400 MHz, 298 K)**

| Colleterpenol (**3**) | | | | 15-hydroxy-*α*-cericerene^28^ | |
| --- | --- | --- | --- | --- | --- |
| Numb | type | ^13^C | ^1^H | ^13^C | ^1^H |
| 1 | CH_2_ | 27.83 | 2.22 (1H, m)  1.93 (1H, m) | 27.7 | 2.19 (1H, m)  1.91 (1H, m) |
| 2 | CH | 126.36 | 5.15 (1H, m) | 126.2 | 5.12 (1H, t, 7.0) |
| 3 | C_q_ | 133.36 | - | 133.3 | - |
| 4 | CH_2_ | 39.61 | 2.10 (1H, m)  2.01 (1H, m) | 38.9 | 2.11 (2H, m) |
| 5 | CH_2_ | 24.78 | 2.23 (1H, m)  2.17 (1H, m) | 24.7 | 2.20 (1H, m)  2.14 (1H, m) |
| 6 | CH | 126.03 | 4.97 (1H, m) | 125.9 | 4.95 (1H, t, 6.8) |
| 7 | C_q_ | 133.10 | - | 133.0 | - |
| 8 | CH_2_ | 38.99 | 2.14 (2H, m) | 39.5 | 2.08 (1H, m)  1.99 (1H, m) |
| 9 | CH_2_ | 24.11 | 2.13 (2H, m) | 24.0 | 2.10 (2H, m) |
| 10 | CH | 125.14 | 5.04 (1H, m) | 125.0 | 5.02 (1H, t, 6.5) |
| 11 | C_q_ | 134.07 | - | 134.0 | - |
| 12 | CH_2_ | 37.62 | 2.10 (1H, m)  2.03 (1H, m) | 37.5 | 2.07 (1H, m)  2.00 (1H, m) |
| 13 | CH_2_ | 28.39 | 1.62 (1H, m)  1.27 (1H, m) | 28.3 | 1.60 (1H, m)  1.25 (1H, m) |
| 14 | CH | 46.66 | 1.45 (1H, m) | 46.6 | 1.42 (1H, m) |
| 15 | C_q_ | 75.80 | - | 75.6 | - |
| 16 | CH_2_ | 40.12 | 1.54 (2H, m) | 40.0 | 1.51 (2H, m) |
| 17 | CH_2_ | 22.32 | 2.08 (2H, m) | 22.3 | 2.05 (2H, m) |
| 18 | CH | 124.37 | 5.16 (1H, m) | 124.7 | 5.11 (1H, t, 6.0) |
| 19 | C_q_ | 135.40 | - | 131.7 | - |
| 20 | CH_2_ | 39.84 | 2.09 (1H, m)  2.01 (1H, m) | 15.5 | 1.57 (3H, s) |
| 21 | CH_2_ | 26.77 | 2.09 (1H, m)  2.02 (1H, m) | 15.2 | 1.57 (3H, s) |
| 22 | CH | 124.37 | 5.11 (1H, m) | 15.6 | 1.56 (3H, s) |
| 23 | C_q_ | 131.54 | - | 24.3 | 1.14 (3H, s) |
| 24 | CH_3_ | 15.64 | 1.59 (3H, m) | 17.7 | 1.63 (3H, s) |
| 25 | CH_3_ | 15.36 | 1.59 (3H, m) | 25.7 | 1.69 (3H, s) |
| 26 | CH_3_ | 15.76 | 1.59 (3H, m) | - | - |
| 27 | CH_3_ | 24.41 | 1.16 (3H, s) | - | - |
| 28 | CH_3_ | 16.20 | 1.65 (3H, s) | - | - |
| 29 | CH_3_ | 17.84 | 1.62 (3H, s) | - | - |
| 30 | CH_3_ | 25.85 | 1.70 (3H, s) | - | - |

Chemical shifts *δ* in ppm, coupling constants *J* in Hertz, s = singlet, d = doublet, m = multiplet.

**Supplementary Table 9 │ Calculated NMR chemical shifts analysis of (14*R**,15*S**)-3 and (14*R**,15*R**)-3.**

| **parameters** | **data type** | **(14*R**,15*S**)-3** | **(14*R**,15*R**)-3** |
| --- | --- | --- | --- |
| R^2^ | C data | 0.9983 | 0.9983 |
|  | H data | 0.9849 | 0.9757 |
| MAE | C data | 2.1 | 2.3 |
|  | H data | 0.17 | 0.20 |
| CMAE | C data | 1.6 | 1.5 |
|  | H data | 0.11 | 0.13 |
| DP4+ | C data | 66.02% | 33.98% |
|  | H data | 100% | 0% |
|  | All data | 100% | 0% |


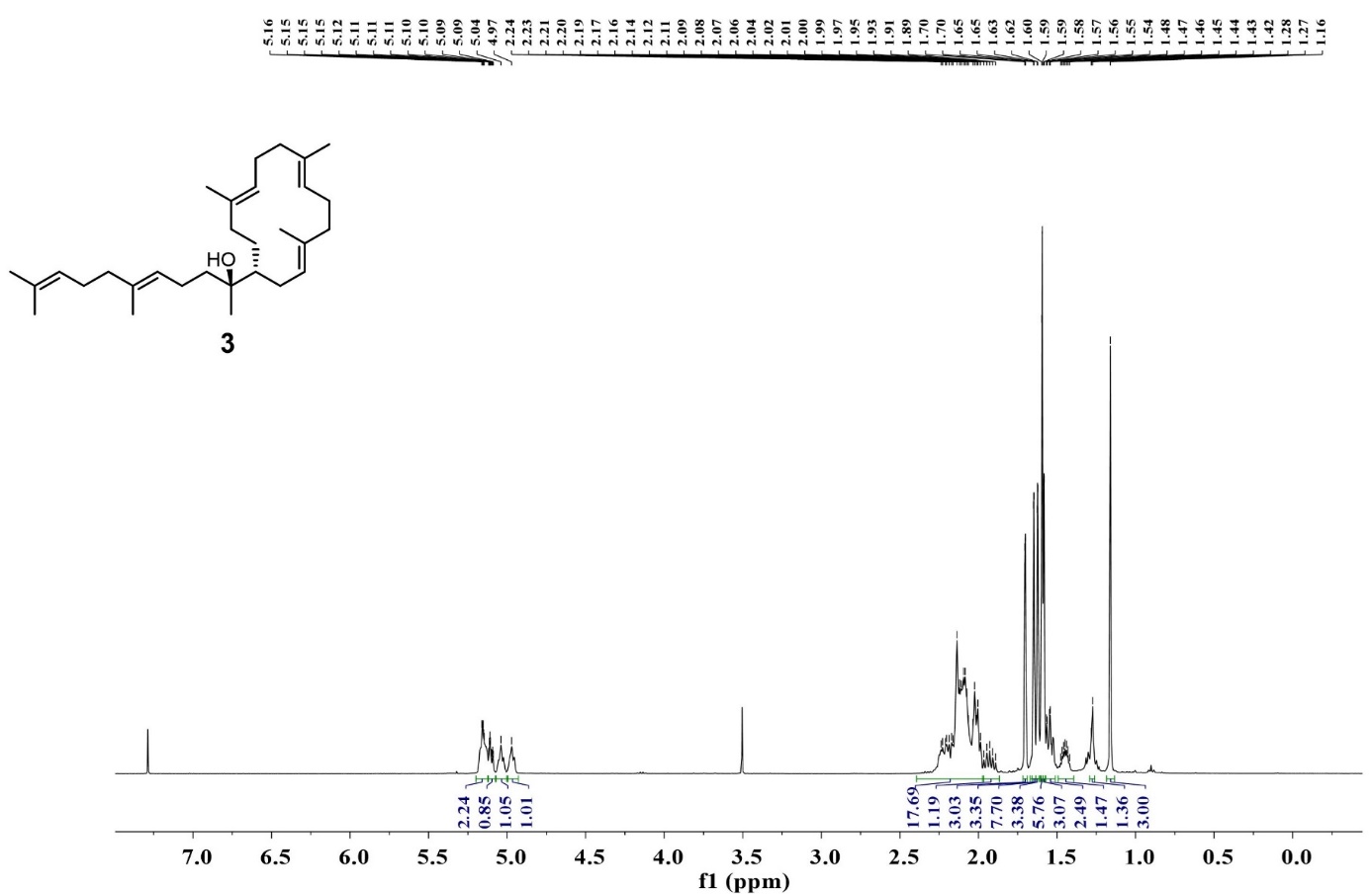


**Supplementary Figure 48 │ ^1^H-NMR spectrum of compound 3 (CDCl_3_, 400MHz).**


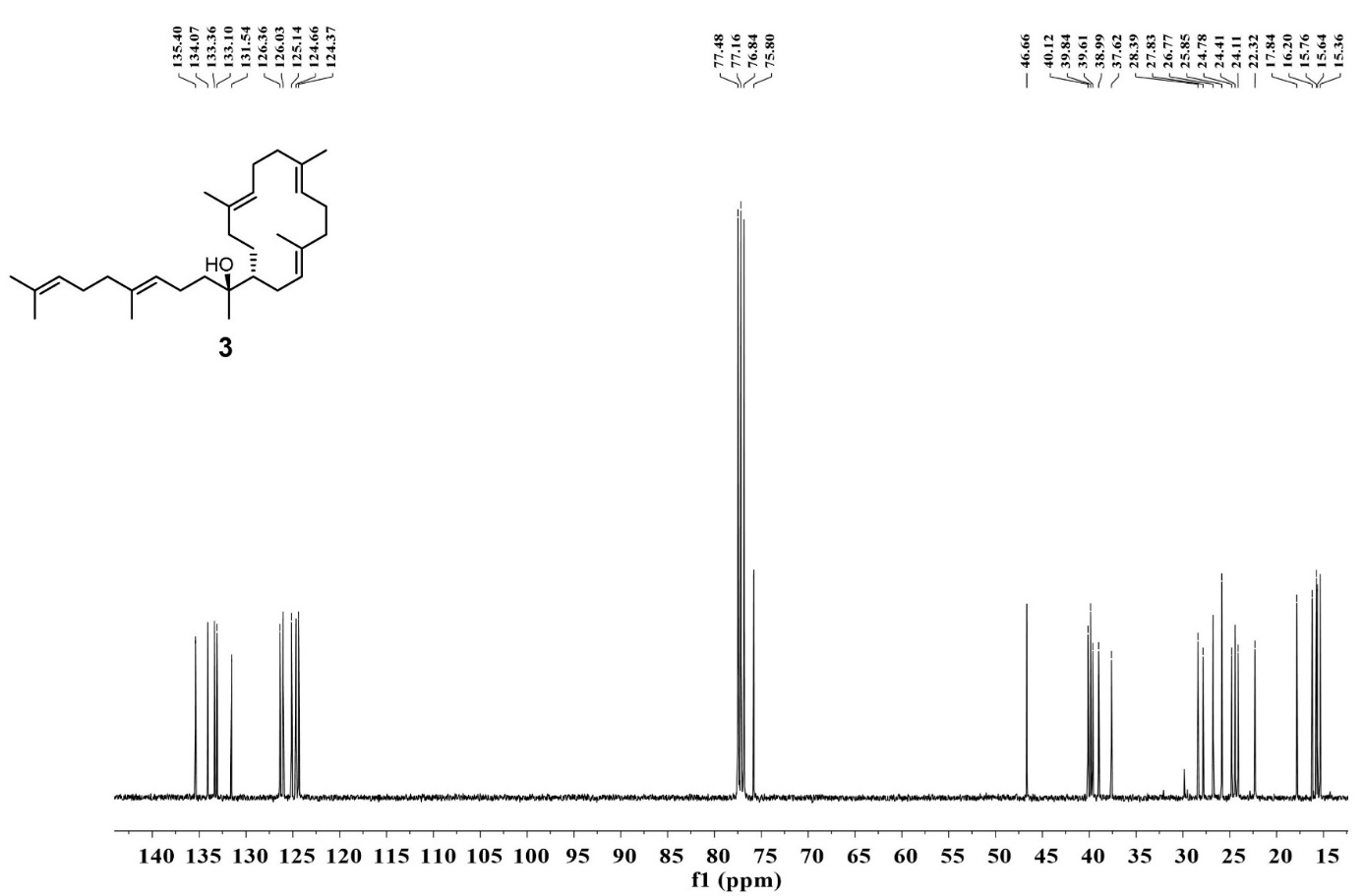


**Supplementary Figure 49 │ ^13^C-DEPT spectrum of compound 3 (CDCl_3_, 100MHz).**


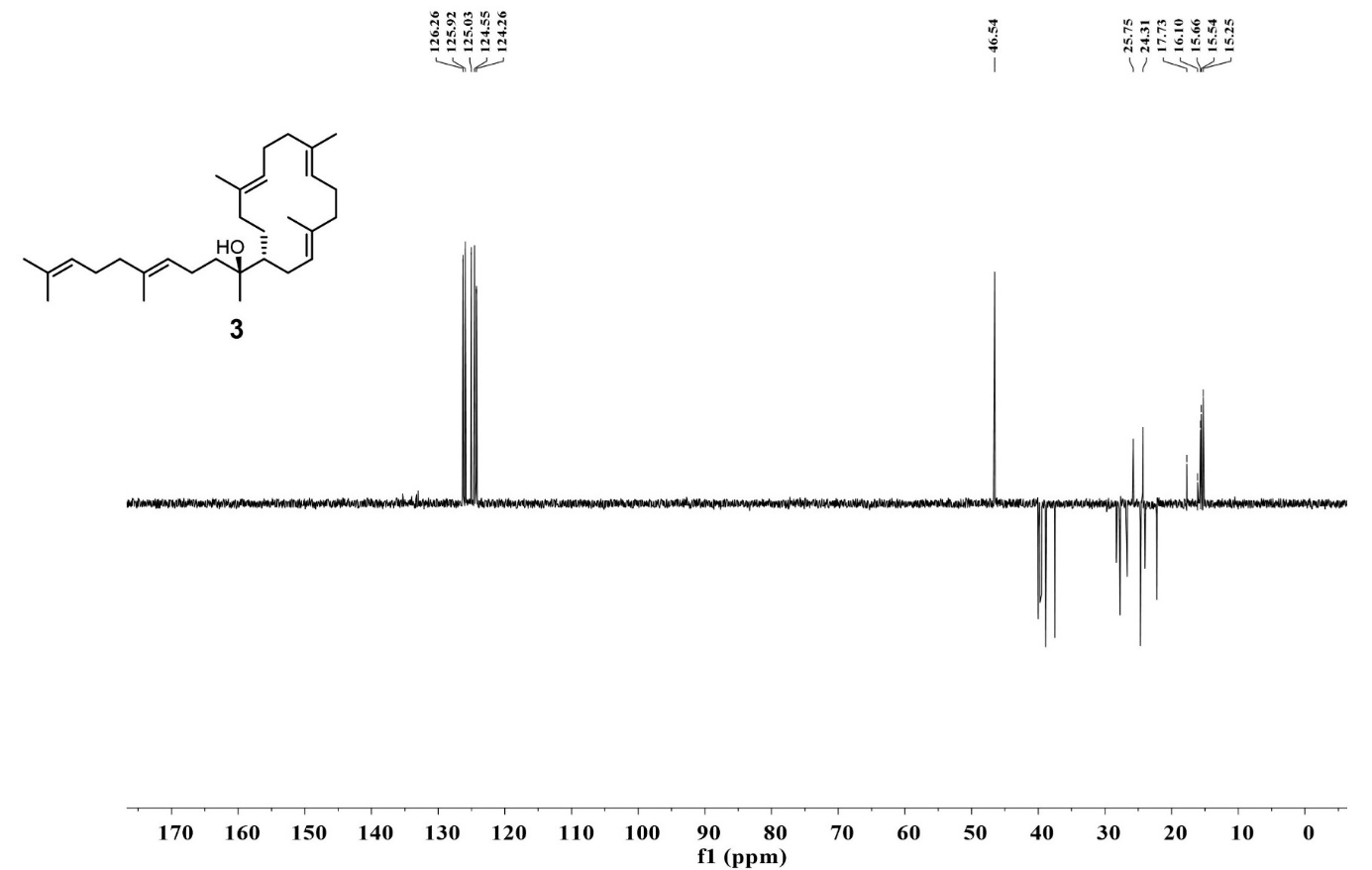


**Supplementary Figure 50 │ ^13^C-NMR spectrum of compound 3 (CDCl_3_, 100MHz).**


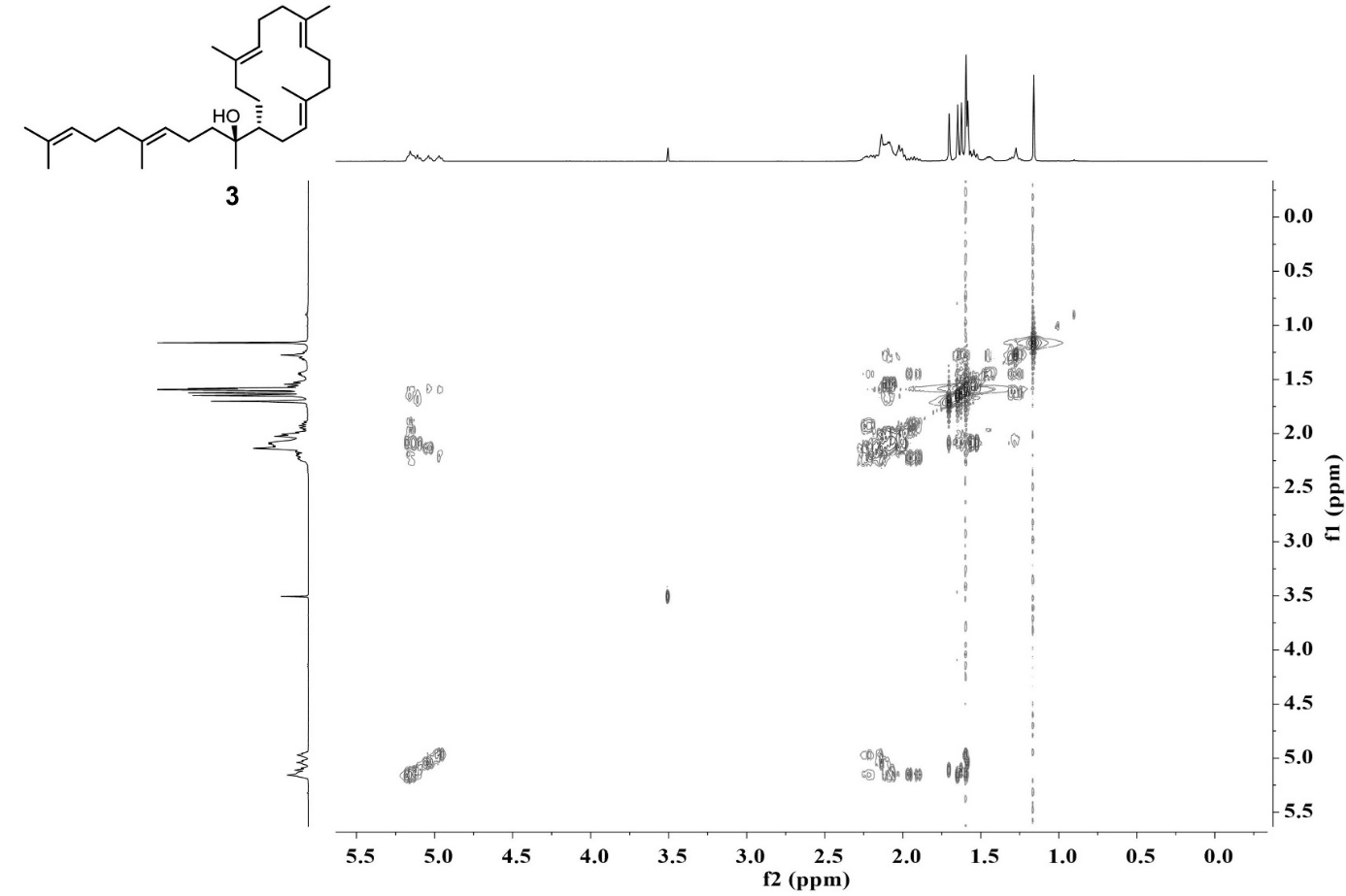


**Supplementary Figure 51 │ ^1^H,^1^H -COSY spectrum of compound 3 (CDCl_3_, 400 MHz).**


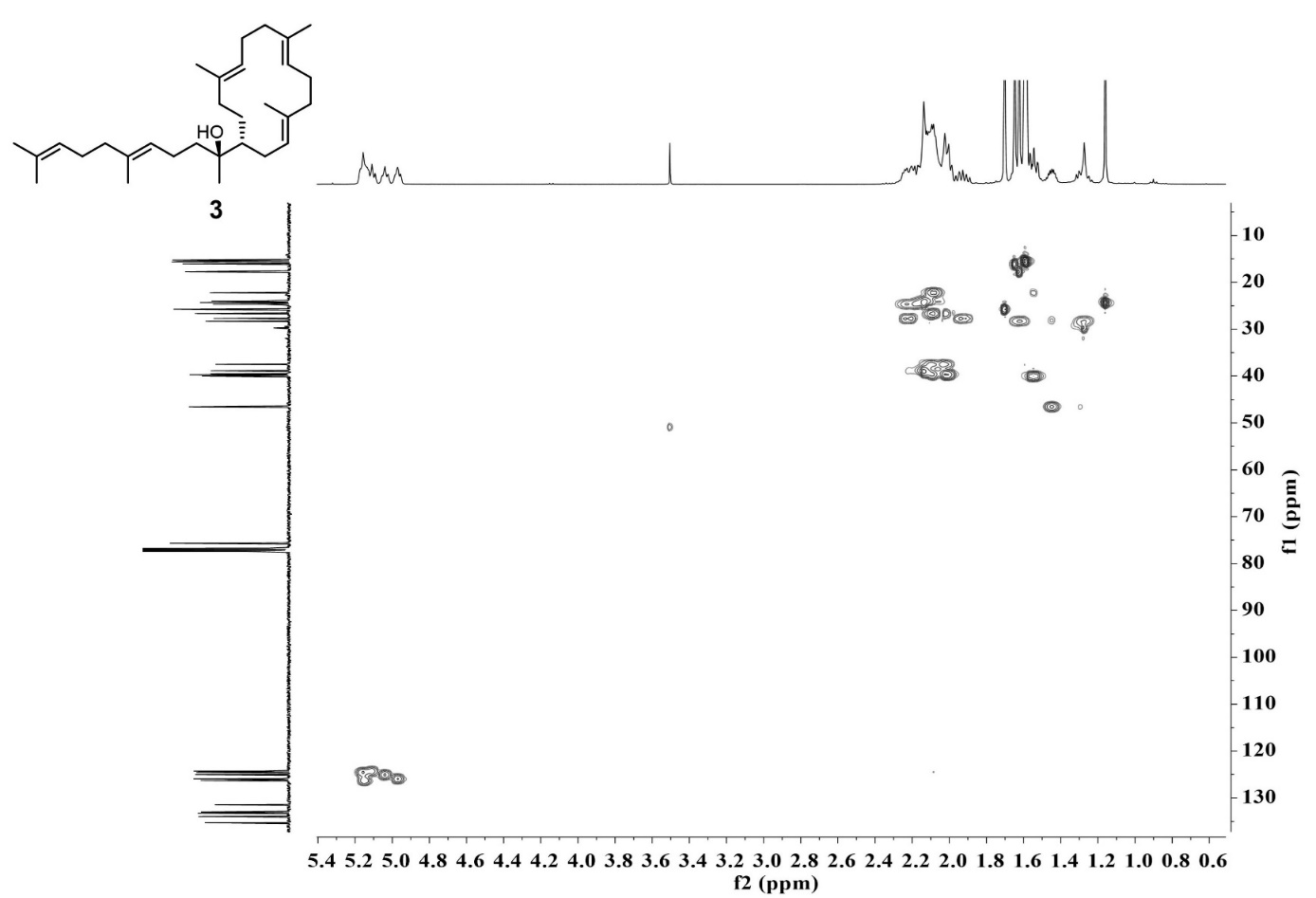


**Supplementary Figure 52 │ HSQC spectrum of compound 3 (CDCl_3_, 400 MHz).**


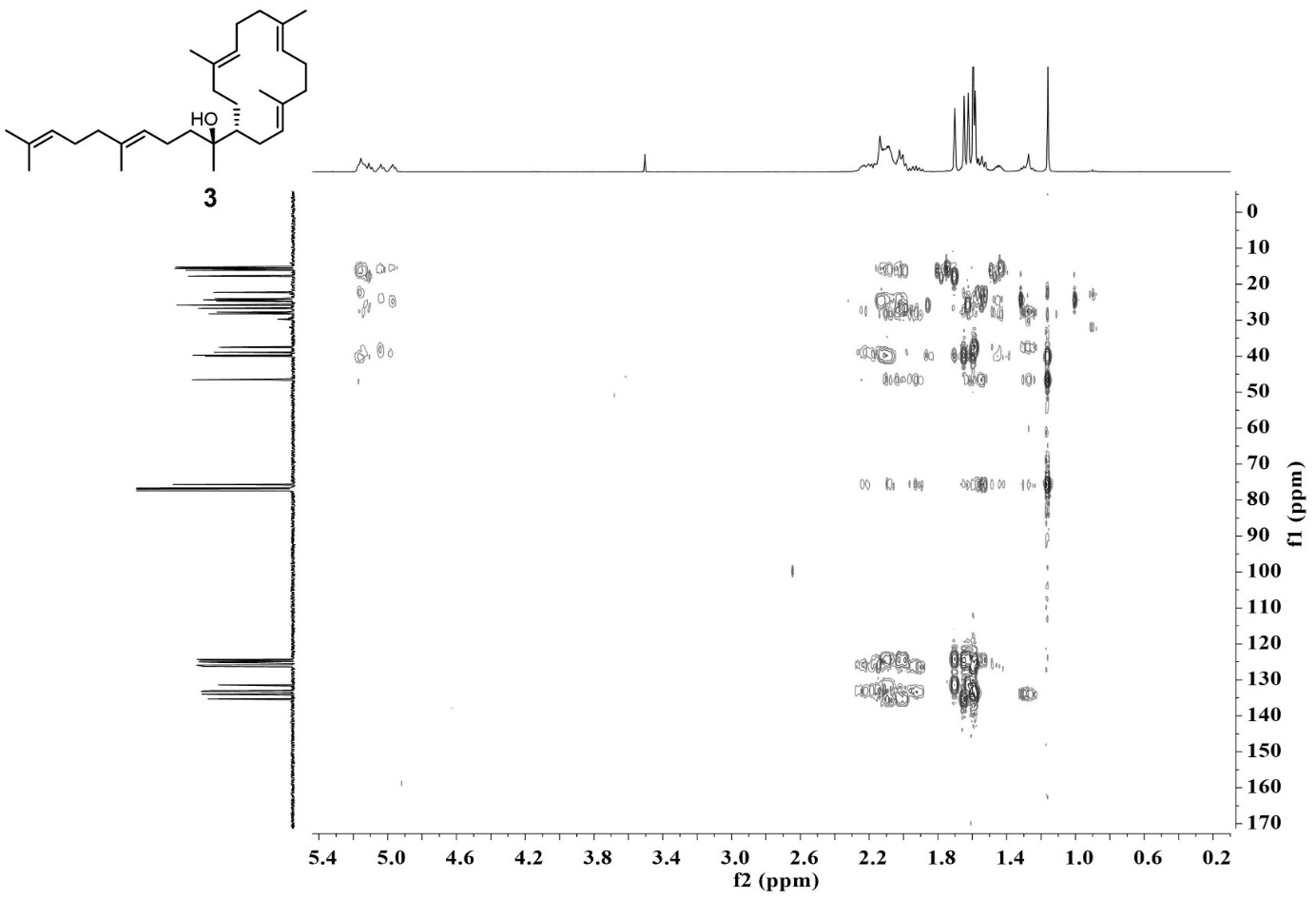


**Supplementary Figure 53 │ HMBC spectrum of compound 3 (CDCl_3_, 400 MHz).**


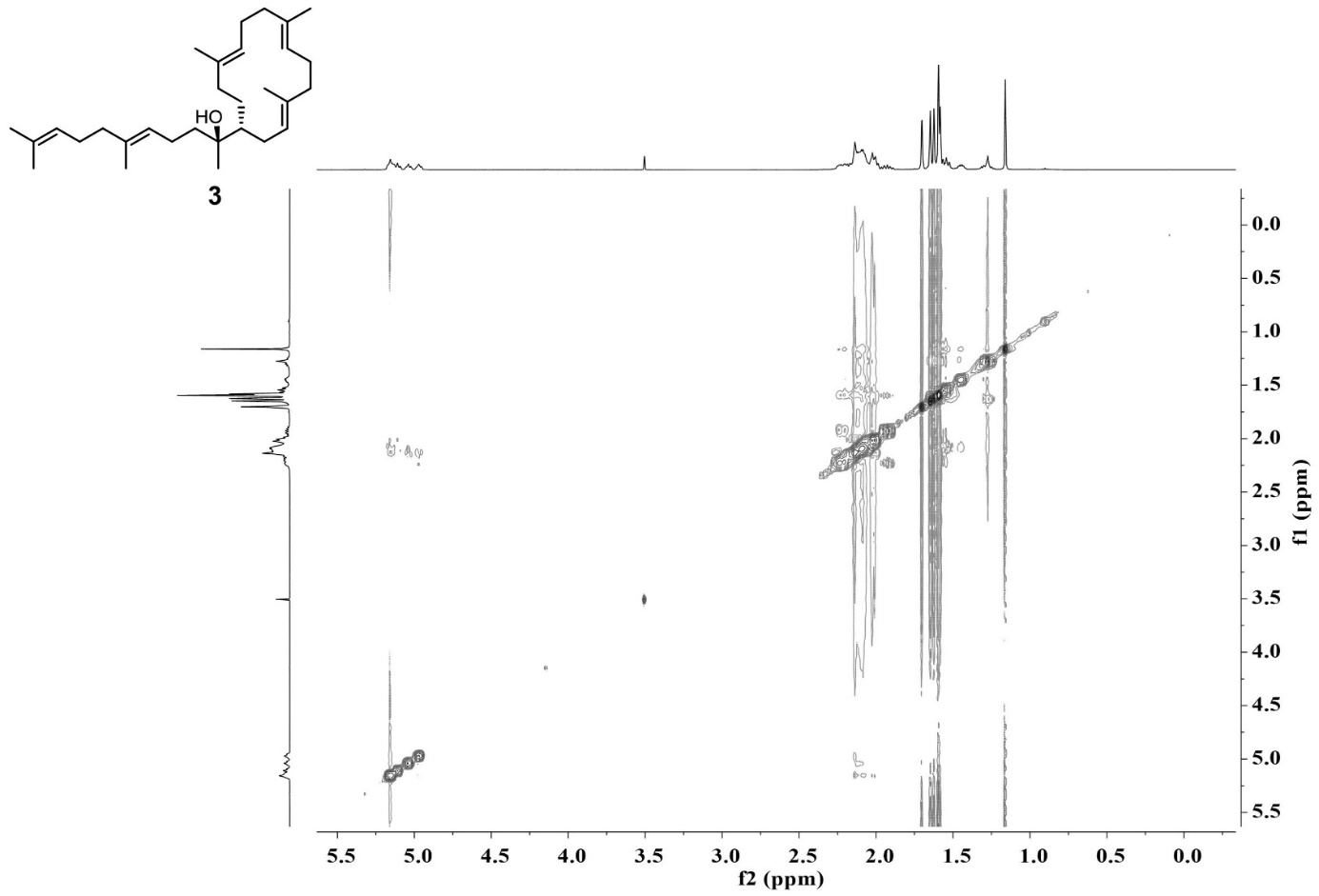


**Supplementary Figure 54 │ ROESY spectrum of compound 3 (CDCl_3_, 400 MHz).**


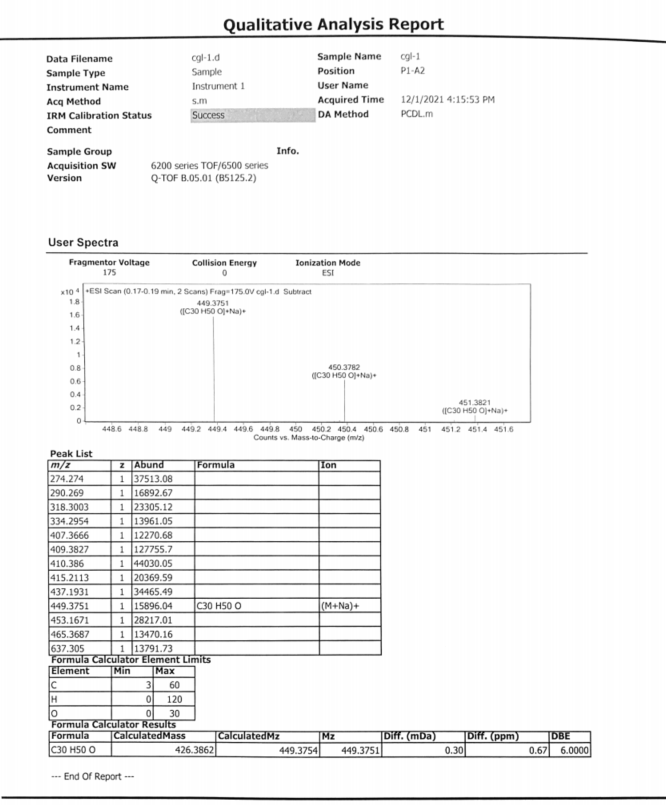


**Supplementary Figure 55 │ HR-ESI spectrum of compound 3.**


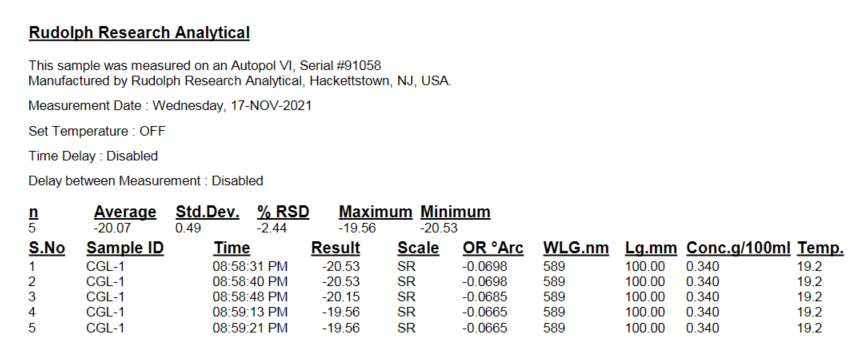


**Supplementary Figure 56 │Optical rotation spectrum of compound 3**.


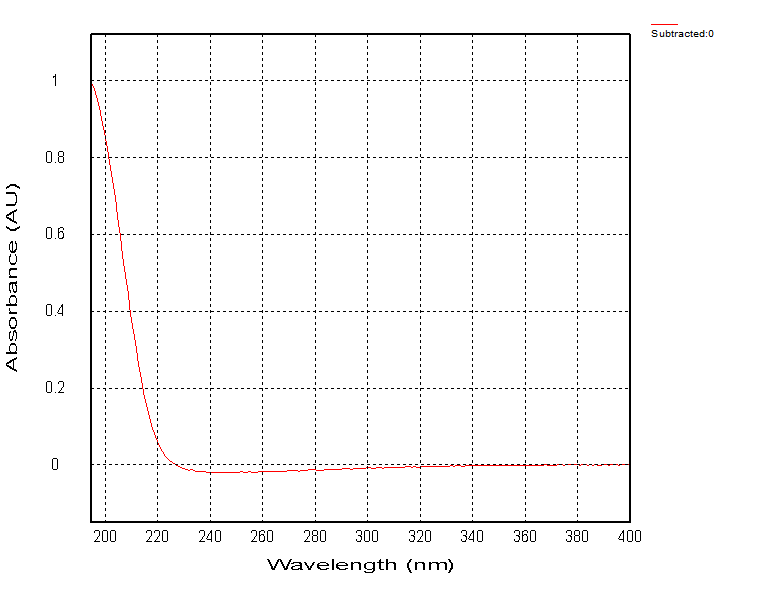


**Supplementary Figure 57 │ UV spectrum of compound 3.**


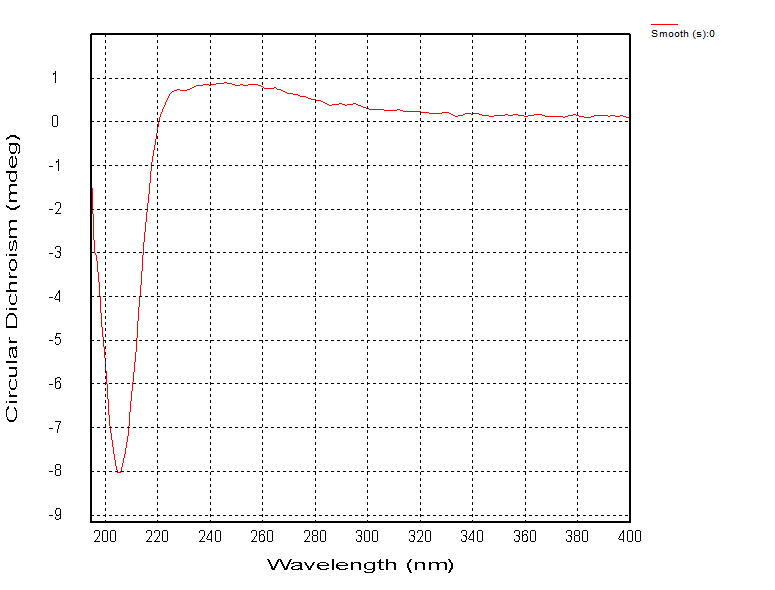


**Supplementary Figure 58 │ CD spectrum of compound 3.**


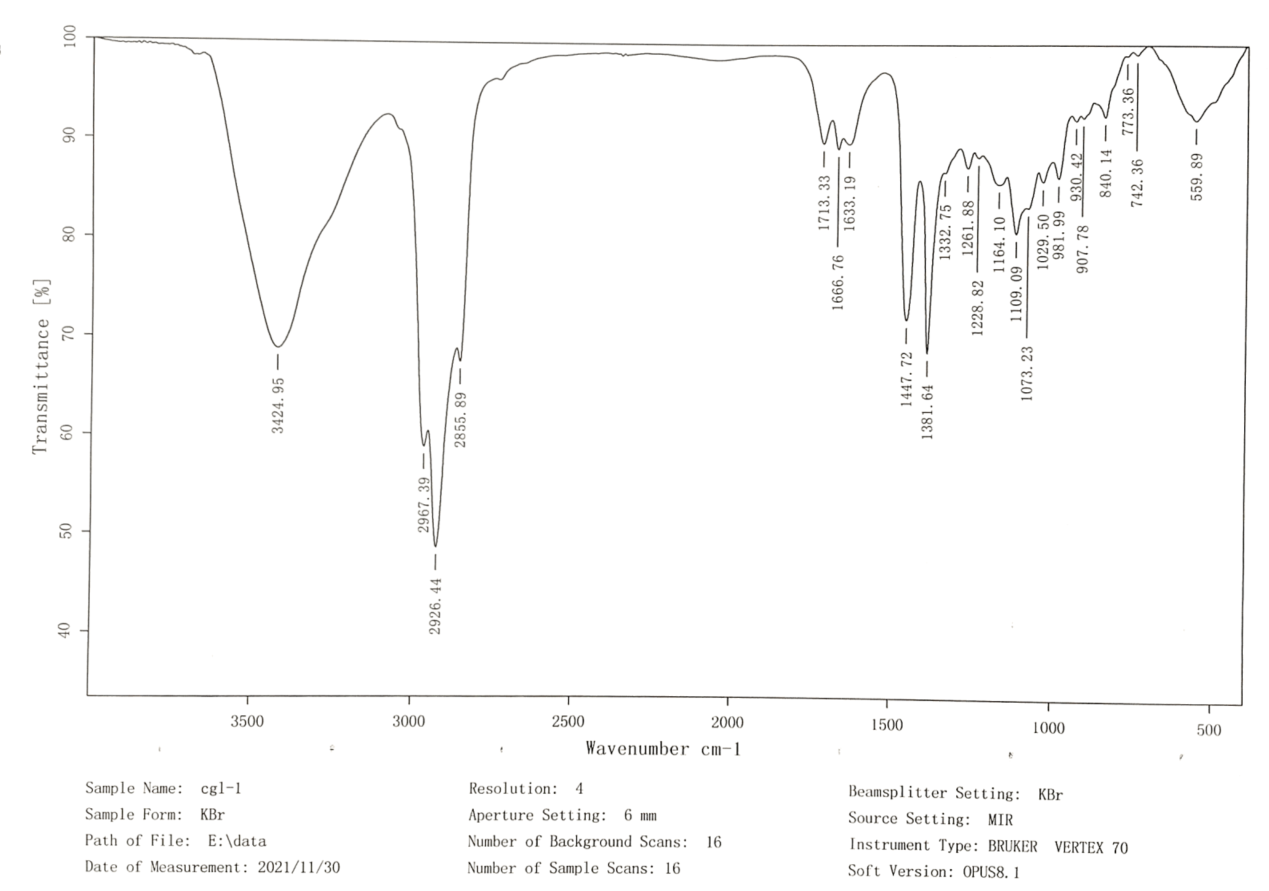


**Supplementary Figure 59 │ IR spectrum of compound 3.**

**Amino acid sequences for AlphaFold2 prediction.**

**QIH97829** (PTTC027)

MYTLDDFPFKYSSLPNPEAERPAKFFNSLPYRVRTIEDDCGSTIDRVVAEWKAITGKGVPDAVGGHASDLIRYVIPECPVEFVAPVTRLIYILLLWDDATDILDPVSHENLMLDFRVGVVSQMKLGYCQCELEMNRIYIQSVLALVVQGAGRNLDGGDFKRGLEVFDSTARGQAPPPQTITWEEYKRHRTTSIGGRLITALIPSMRKLRLDQDALDSVSHLAEICYLIAGLSNDFYSFHKEFEEHARAGSLDGIHNGMAVLMSRYGYEESEAEEIMKKEILSAERTLMEKYEEWKSSSAPKSDELRHFVLISILAVGGASYWQSFSPRYQRANLSTTVEDRAQLVGRSYTAGLRLPNYPVPVAMTKSTNGYSESLTNRQASLITKDLLAPFEKAPAEEVVLAPWKYTQSLPGKRTVGRMIECLQAWFSLPVESIKIISEITTMLFNATLMLDDIQDESQLRRGKPAAHAVFGQAQTINSATYLYVKGSRRLRGVKHADECADALLDELETLAFGQALELQWKYQKICPSTKQYLVMIDNKTGGFFRLVLRLLGAEAQSQPVPELMHLLTLLGRYYQIRDDYMNLASDEYTAKKGFCEDLSEGNFSFPLLHLLQHSPNADIVRGVMFHREDKADLSIEMKQFILAEMKEVGSLGYTMNLLEELFSAMLGALDNVEAKLGLNKKLRIFLLLLKV*

**KFX89132** (PTTC035）

MKSQVTFQKRSIRKLLRILSRKQPKAQATPEEEPFIYSKPLQDAYPEPSGSFTVLPCRIGLQDDGVQAAIRRLDDDGTVIGKLNNGTNEPSIAKLNSPIGHYVTLTFPECIPTRLEIVTLFVETAALLDDVLEDDSNAKAKQDFGDALMGAKLPAITSPRKAAMQKWAAKSSLEVVTIYGKEGRVLLERCQSSFQDQWATETDVEIKATKFEDYLSQRIRNFAMPGLWATTAFSIGVLLDPAEYKLIQPLTDIATRIIIQTNDYFSWDVERSRGEGLAFNGIFCLMKEHHITEEHAKSRLKSYIMQDEQAYVSMLDDFYETHPNLPIHVRKYVSACAFVVSGNHHWSAACPRYHASQTDEKKELRDQSVEIETKSMKLEPEGVAIDNPSISGPASNKYHSLTEIDEAILGSSALLAPQRYIQSLPSKNIRSKLVDAFNIWFQLPEDLIDTIKGIIDDLHNATLMLDDIQDESILRRGSSAAHCIFGPAQCINSSTYMVVQAAGRINARYTESPQLMGIFLEGLRSLAIGQSWDLNWKFNSYCPSIAEYMVMIDGKTGAMFRMLVNLMKSLSSLQTWPVADFDRLIQLLGRWYQVRDDYQNLKDPEYTEQKGFCEDLDEGKLSYPVVLTCNSDPVARTIILGIFRRKDTGTPLPRSVKMQILDLIQKSGALVKTWQLVQQLEKEVEDTLSALETVLGEQNPSLRLITKLLSDILPP*

**CRG86078** (PTTC044)

MSILDDFPYQHSRTLEQSELPEGYFHILPFRVSGPREGMLEAARAVKSEWAKTVGKEDTPLTADEKDSPLMYVMPECPPDMVAPIVRFGTLVVLWDDETDALGGEQHRAMADDYCLGLISEYKLGKRSPCDFKINNLYLDSVFQLSKQVASDDHTYKRALQAVEHTMRAQPVPPLHEVTFEEYKKHRTVSSAGKLIEHMLHSLHGLKISQEEQDSVSPLIEHGCLALGLVNDLYSFPKEFEEHTQSGNIDVIHNAMAVLMSNYGYTESESKEILKQEILSIERDVLDEYEAWKNSPVYKSPDMRRYMVLAMLAFGGGCYYQARSPRYHGRKLTTSEIDRAQLVGRSHTAWRLPGHAPPKSFTKDGTLLTAEAESLKPEKEEDECTTASEIPDILAPFSKAPAEELCMAPYNYTKSLPGKKTVGRLVECLRVWFQLPSDSITIIEDVVTTLFHATLMIDDIEDGSILRRGKPAAHIVFGMSQTVNSATKVFLDELETLVFGQTFDIYWKFHKTCPKTQEYLTMVDNKTGGFFRMALRLIQVEASAEPCHDLSHLITLMGRYYQIRDDYLNLTSEEYTAKKGFCDDLSEGKFSFPLIHFLQNSSTADIVRGLLFRQENGSSDLTPEVKTYIVSEMRDAGSLTHTRDTLKDLFDAMMEALERAESSLGTNKRLRAFLILLKIE*

**NMDCN0000RG9** (PTTC058)

MELLYSDPVEPQIVARSCPTRLAVRKSRHSHIADLATREFMEEWNGADGQGLCNSAGPCGSLIALALPEAKPERIASVTKATEFLCAVDDVVDSMESIESVSTRNRETRDLVWKLLLAKGRHDWGTRKTQLCTKILLELLAIDPVRGQVVIEALNQYRSSLGGTRADQIRDWDKWLAYRWESGGCGMFLVFIFYACELDLTQNDLDTVRHITWKAMAITILSNDLYSFEVEAMLGIQASGNISNGVWHLMKDRNITVEEAKHILLTEKLRPLEEQFLEEKAGFLQDHGKELPQLAHYLDLVELAASGNWYWSSQCYRYHSWRENVTQFGDRPISSFNIQILDETTAKCHDYLQAAANNDQASRIEEAETSIKDQTAILNNGSGNPREDQRQYLDQKLVLAPISYLECLPSKKIREIVIEGLSSWFTIPKDTLDRLERVISNIHNASLLIDDIEDGSPLRRGRPAAYRVFGRAQTINSANFLWVTTTEETFHFNKNSREVFIDELKVLCIGQSYDIYWTHTASCPSLEEYLEVCRSKTGRLFSMIGRVLHSESEQPRAINVDDLENFLLLLGQFFQIRDDYINLASEKYEKEKGFAEDLDEGKFSLPMIHLLNNSPDRLMIEHILHQRSRDGGMSPEMKMLVLEKMHEVGSLEFTRETLRSVETKLEGGELDIVVWFMRMVAFWPQANRLLATVT*

**KAF2708718** (PTTC059)

MEYQYSKILSPSEYRTDGLCDGIEVRKNIAADLEEIGTIQAQEDWSNLVERLEGYKGGLGPMHSFMAVSLPECLPERFEIVCYANEFAFLHDDMTDVVDKQASDAANNDMLDAFHHNQETKTSDCIRSGKRQMQAKILNTMMQLDKDRTMTAMKAWASFLEQGSGRQHDTAFRTLEEYLPYRCKDVGHMFWHALVTFGCALTIPENEFELCEELVLPAVIAASLTNDLFSYEKELEAAQKSGRLDVVNGLWYLMLEHKLTLEEAKVRCRNRIKVEVAKYVQTVQEVRYRVDLSDDTKRYIELMQYSISGNVVWSQQCPRYNKQATYNQRQRLRMTHGDPQYPTIQQLPTNSQVALPCCETRETVEQGKAEGSEASPEPLEVTLGEVISLSDSGPFPMKRSESICACNTADMAFCMPLPLSAVDVILEPFNYISALPSKGVRDMVINALECWLKVPEESGAIIGTVINIMHTSSLMLDDVQDRSPLRRGQPSAHTIFGESQTINSATFQYLEAIAQLRRLNNPKCIDIFIEDVRSIFVGQSHDLLWVWEQICPSVSEYLQMVDGKTGGTFRLLIRLMAAEAGFGVVARLDRLCRLLGRYFQIRDDYQNLVSREYNAQKGMCEDLDEGKYSLPLIHALSHTVKSHQIRGLISQRHIAGGLTVEQKRLMLDLMHEAGSFAYTVEVLYALHSELMTEIERLEEMFGKANHEFRLLLEILKV*

**NMDCN0000RGA** (PTTC060)

MKLKVKIRKALSGRSRSSILSTVPDADVFLHSEPLENVHQRPSDAFTVLPSRLHKQDAQTVESAKLFSTEMMAAISGRRSDHPCHVGSCVGHMSSLLYPECLPERLDTVAMILEGGTIHDDIVERDDLERAMDHHRMVTSILGTTQGVGAIQSVRGLRGKEFVSKVALKFTAKYGAEGKVLGEKCLEYWLQPMEFVVKSNPTSFEDSVSNRIINIGTRAIWGILEFSLGILLSDDEMKQIKHIVDVADRIFANTNDYFSWEVERDNGNRVQNSIKVLMEAEGRSEEQAKETLKNAILKDEKTYQHLSKQFFLSSPNAPPHVQRFIAMLEVLLGGHHIWCAACERYKFHTLSDGKGINICTVEEKSENMAPQYSSCSTLDDSALLDPVHYIQSLPSKNMRSKVIDALNIWCRLPDDQLDTVKSVVDDIHNSTLILDDIQDASFLRRGFATAHHVFGPGQCINSATYMVARAASRVATHQDQHPKLIHILLHGLNQLAVGQSWDLNWKDTGHCPSTAEYLAMVDGKTGAMFALIIQMMHDFSSIPDWPISELNQLAKLLGRWYQIRDDYQNLQDEQYTSQKGFCEDLDEGKLSYPLTVCCGLDPMAQRVIMGIFRQRQSGTPLALNVKTQILDILRRTGAFQQTWEVLKNLERNVGAALSNLEAITGEPNPKFRAIVSLLGDIARPTQDCE*

**NMDCN0000RGB** (PTTC062)

MTIHQYSVEVDPEIVKSVSCFTSLPIRISTSNKLADDLCNRFLEGLRNLRSPSVYSPTLCQSSVGNFLALLLPECPPDRFQPIAQIANLMWFVDDSVEYAPTRDEKTSTHENTISILSLFSHPTKRPFNSKPDYMQFISKIFLECLERPNEEFFLNCIKSLFDNHGTLLSDPTDCRTLDEYMSLRWIHGGTLTAILILCHAMDIQLTDAEKKNIDAVINPLVKSMLLTNDYYSYRKEELLHRLRKNPGHPFNAVSVLVVERNISEEDALELLKRIIIEAEEQHAVIFDEMQRSNELSGEQLRYVEAVRLSAAGSSLWHAASPRYSSALPPAADGTVPEFEKTIDNDTRSNDLIPEDQNGCRLVERNHFAPLSLEERLLDPSRLKPSDEIVTAPFDYVASFPSKRIRELFIHSLCAWFDVCIPDVDQITSIVGQVHNTTLMLDDIEDNSTLRREQPCAHLIFGNPQTINSANYAFLLLMDMVQKLSNPESLAILLDEIKNLGRGQGLELHWRHCKICPTMDEYVTMVDNKTGSLLSLGMRLVQAQSSTNCDIPEITALTTLFGRYFQIRDDLKNLTSDEYTAQKGFCEDLDEGKYSFPLIHCLNSTTNDKSMILGMLYNSPRDGLTVEMKRFIQTHMREKTGSFAFTQDILEKMKDEVFRLIGVAEDKTGKENPMLRLLIRGLSR*

**KAH9237577** (PTTC074)

MPTFAQPVPDDIVASSGLRSKFRPHVHGNYQICVEPSKGMETFYNDAMSTQLESKTLPDIPGLGLVHPMALAMANCLPERLPAITRFADFTILNDDYYDIAKRDEIEKVNNDIQDALQDASAPGKTQSSGSSDIDFKPKQMQAALVLELIMLDQQLAMDIMSSYSQGLDVATFAPDNLRTLDEYLPVRKVNSGLDVTAEMVCFGMGLRISPSDKAKLRPVVDLANFAITVVNDLYSWPKEIKCHLETPGSELPFNAVAVLMRHGGYSEPEAFRILYAKQAELEAEHLRQLDALRAQEGGRLPENQELYVENAQRAVCGSELWSVYTRRYPSKADLRQPEVEFVDGAFRYVADGEVSGEEKEHITNVKEVCEDETDGVRDMSDISQKSKKKIDTLLPEGPGLTTYASRLEAAPDHAVIAPIKYLATLPSKGVRDTFIDALNWWLEVPEDSLRTIKTIISMLHDSSLILDDIEDDSTLRRGSPAAHMIFGTAQCINAANHIFVMVLAELQKLRSPLKTAILIEELESLFVGQADDLHWKYHVDCPSTEDYMEMIDNKTGGLFRLCVRLLQAESTRTDVLDLDPRPFVRQLSLFFQIRDDYQNLVSDAYAKQKGFAEDLDEGKISLPIILTLQRARTRPEIMGVLKHKQPGPMAMEMKQYIVREMEKCGALESTRELLQGMQEDLIAELRRLEGDFGAKNATLELVLRRLWIS*

**KIK55704** (PTTC114)

MEFQYSTVIDPSTYDTEGLCDGIDFRRNNFTWLEDRGAIRAQADWTKYVSPATGHRGVLGPQYSLLSSAIPECSPERLEVISYALEFGFLLDDVINATDQEQGTIESNDMMQAFLEGVQTGKITKNDAQTKREGKRKIQSQLLLEMFSIDRERAIAFVKAWAEFAEVGSGRQHHENFATLEEYLPYRIVDAGHALWYTFITFGMGLNIPQWEREKCDELTQSATAALVLQNDLFSWEQEYATAQSNHQSHVTNALRVLMREHNIGIQEAQQMCRKLIKQHVSDYIQIVENVKHNESLSADLRKYIEAMQYTISGNIAWSMNCPRYHPQASLNETQLEWMHSGVPDKLTFSLPSPPASPEVLGSLSPSWSVHSDSSRSSTPPLEEPITATKNLLMNLELPSPPPPSEIVEAPYQYIASLPSKGIRDKFIDAVNQWLKVPENIVEQIKALTNRLHQASLLLDDFEDSSPLRRGKPAAHTIFGAPQAINSAGYCIVKAIGELQALGASQIITSKLILTSSDKILSLFKGQALDLHWTYNGICPTPAEYIQMIDCKTGAQFDLVVDMMLAHSNASVKPDLKKLTTLLGRYFQIADDYKNLVSADYRKQKGFCEDLDEGKYSLPLIHLLQSHPENLQLRNILSTRRAEGKMMYEQKVLVLEYLREAESLEYTHSVLEGLHAKIGQQIDNIEESFGETSIELRVLWELLRV*

**Supplementary References**

1 Bian, G. *et al.* Production of taxadiene by engineering of mevalonate pathway in *Escherichia coli* and endophytic fungus *Alternaria alternata* TPF6. *Biotechnol J* **12(4), 1600697.**, doi:10.1002/biot.201600697 (2017).

2 Gibson, D. G. Synthesis of DNA fragments in yeast by one-step assembly of overlapping oligonucleotides. *Nucleic Acids Res.* **37**, 6984-6990 (2009).

3 Qin, T. *et al.* Protoplast mutant selection of *Glarea lozoyensis* and statistical optimization of medium for pneumocandin B0 yield-up. *Biosci., Biotechnol., Biochem.* **80**, 2241-2246 (2016).

4 Zivanov, J., Nakane, T. & Scheres, S. H. A Bayesian approach to beam-induced motion correction in cryo-EM single-particle analysis. *IUCrJ* **6**, 5-17 (2019).

5 Rosenthal, P. B. & Henderson, R. Optimal determination of particle orientation, absolute hand, and contrast loss in single-particle electron cryomicroscopy. *J. Mol. Biol.* **333**, 721-745 (2003).

6 Scheres, S. H. & Chen, S. Prevention of overfitting in cryo-EM structure determination. *Nat. Methods* **9**, 853-854 (2012).

7 Chen, S. *et al.* High-resolution noise substitution to measure overfitting and validate resolution in 3D structure determination by single particle electron cryomicroscopy. *Ultramicroscopy* **135**, 24-35 (2013).

8 Pettersen, E. F. *et al.* UCSF Chimera—a visualization system for exploratory research and analysis. *J. Comput. Chem.* **25**, 1605-1612 (2004).

9 Grimblat, N. s., Zanardi, M. M. & Sarotti, A. M. Beyond DP4: an improved probability for the stereochemical assignment of isomeric compounds using quantum chemical calculations of NMR shifts. *J. Org. Chem.* **80**, 12526-12534 (2015).

10 Zanardi, M. M., Marcarino, M. O. & Sarotti, A. M. Redefining the impact of Boltzmann analysis in the stereochemical assignment of polar and flexible molecules by NMR calculations. *Org. Lett.* **22**, 52-56 (2019).

11 Pracht, P., Bohle, F. & Grimme, S. Automated exploration of the low-energy chemical space with fast quantum chemical methods. *PCCP* **22**, 7169-7192 (2020).

12 Frisch, M. *et al.* gaussian 09, Revision d. 01, Gaussian. *Inc., Wallingford CT* **201** (2009).

13 Lu, T. & Chen, F. Multiwfn: a multifunctional wavefunction analyzer. *J. Comput. Chem.* **33**, 580-592 (2012).

14 Bian, G. *et al.* Metabolic engineering-based rapid characterization of a sesquiterpene cyclase and the skeletons of fusariumdiene and fusagramineol from *Fusarium graminearum*. *Org. Lett.* **20**, 1626–1629 (2018).

15 Hu, L. *et al.* Draft genome sequence of *Talaromyces verruculosus* (“*Penicillium verruculosum*”) strain TS63-9, a fungus with great potential for industrial production of polysaccharide-degrading enzymes. *J. Biotechnol.* **219**, 5-6 (2016).

16 Islam, M. S. *et al.* Tools to kill: genome of one of the most destructive plant pathogenic fungi Macrophomina phaseolina. *BMC Genomics* **13**, 493 (2012).

17 Huang, A. C. *et al.* Unearthing a sesterterpene biosynthetic repertoire in the Brassicaceae through genome mining reveals convergent evolution. *Proc. Natl. Acad. Sci. U. S. A.* **114**, E6005-E6014 (2017).

18 Lauterbach, L., Rinkel, J. & Dickschat, J. S. Two bacterial diterpene synthases from *Allokutzneria albata* for bonnadiene and for phomopsene and allokutznerene. *Angew. Chem. Int. Ed.* **57**, 8280-8283 (2018).

19 Rinkel, J. & Dickschat, J. S. Addressing the chemistry of germacrene A by isotope labeling experiments. *Org. Lett.* **21**, 2426-2429 (2019).

20 Klapschinski, T. A., Rabe, P. & Dickschat, J. S. Pristinol, a sesquiterpene alcohol with an unusual skeleton from *Streptomyces pristinaespiralis*. *Angew. Chem. Int. Ed.* **55**, 10141-10144 (2016).

21 Rabe, P. *et al.* Mechanistic investigations of two bacterial diterpene cyclases: spiroviolene synthase and tsukubadiene synthase. *Angew. Chem. Int. Ed.* **56**, 2776-2779 (2017).

22 Quan, Z. & Dickschat, J. S. On the mechanism of ophiobolin F synthase and the absolute configuration of its product by isotopic labelling experiments. *Org. Biomol. Chem.* **18**, 6072-6076 (2020).

23 Rabe, P. *et al.* Conformational analysis, thermal rearrangement, and EI‐MS fragmentation mechanism of (1 (10) E, 4E, 6S, 7R)‐germacradien‐6‐ol by 13C‐labeling experiments. *Angew. Chem. Int. Ed.* **54**, 13448-13451 (2015).

24 Mitsuhashi, T., Rinkel, J., Okada, M., Abe, I. & Dickschat, J. S. Mechanistic characterization of two chimericsesterterpene synthases from Penicillium. *Chem. Eur. J.* **23**, 10053-10057 (2017).

25 Bian, G. *et al.* A clade II‐D fungal chimeric diterpene synthase from *Colletotrichum gloeosporioides* produces dolasta‐1 (15), 8‐diene. *Angew. Chem. Int. Ed.* **57**, 15887-15890 (2018).

26 Baj, A. *et al.* Convergent synthesis of menaquinone-7 (MK-7). *Org. Process. Res. Dev.* **20**, 1026-1033 (2016).

27 Cornforth, J., Cornforth, R. H., Popjak, G. & Yengoyan, L. Studies on the biosynthesis of cholesterol: XX. Steric course of decarboxylation of 5-pyrophosphomevalonate and of the carbon to carbon bond formation in the biosynthesis of farnesyl pyrophosphate. *J. Biol. Chem.* **241**, 3970-3987 (1966).

28 Chen, Y. G. *et al.* A cryptic plant terpene cyclase producing unconventional 18‐and 14‐membered macrocyclic C25 and C20 terpenoids with immunosuppressive activity. *Angew. Chem. Int. Ed.* **60**, 25468-25476 (2021).
