# Supplementary material for: Discovery of non-squalene triterpenes
Source: Nature. 2022 Jun 1;606(7913):414–9. doi: 10.1038/s41586-022-04773-3 (PMC9177416; doi:10.1038/s41586-022-04773-3)
Supplement: Supplementary file 2 — Reporting Summary [file 41586_2022_4773_MOESM2_ESM.pdf]

nature portfolio

Corresponding author(s): Tiangang Liu, Jeroen Dickschat, Ikuro Abe

Last updated by author(s): Apr 4, 2022

Reporting Summary

Nature Portfolio wishes to improve the reproducibility of the work that we publish. This form provides structure for consistency and transparency in reporting. For further information on Nature Portfolio policies, see our Editorial Policies and the Editorial Policy Checklist.

Statistics

For all statistical analyses, confirm that the following items are present in the figure legend, table legend, main text, or Methods section.

n/a	Confirmed	
☐	☒	The exact sample size (*n*) for each experimental group/condition, given as a discrete number and unit of measurement
☐	☒	A statement on whether measurements were taken from distinct samples or whether the same sample was measured repeatedly
☒	☐	The statistical test(s) used AND whether they are one- or two-sided<br>*Only common tests should be described solely by name; describe more complex techniques in the Methods section.*
☒	☐	A description of all covariates tested
☒	☐	A description of any assumptions or corrections, such as tests of normality and adjustment for multiple comparisons
☐	☒	A full description of the statistical parameters including central tendency (e.g. means) or other basic estimates (e.g. regression coefficient) AND variation (e.g. standard deviation) or associated estimates of uncertainty (e.g. confidence intervals)
☒	☐	For null hypothesis testing, the test statistic (e.g. *F*, *t*, *r*) with confidence intervals, effect sizes, degrees of freedom and *P* value noted<br>*Give P values as exact values whenever suitable.*
☒	☐	For Bayesian analysis, information on the choice of priors and Markov chain Monte Carlo settings
☒	☐	For hierarchical and complex designs, identification of the appropriate level for tests and full reporting of outcomes
☒	☐	Estimates of effect sizes (e.g. Cohen's *d*, Pearson's *r*), indicating how they were calculated

Our web collection on statistics for biologists contains articles on many of the points above.

Software and code

Policy information about availability of computer code

Data collection	CCREST 2.8, Gaussian 09, Thermo Xcalibur 2.2, Agilent MSD ChemStation D02.00.237, Shimadzu GCMS solution 4.41, EPU
Data analysis	MEGA7, MestReNova 5.3.1, PHENIX-ver 1.19.2-4158-000, ccp4-7.1, XDS ver Jan 31 2020, Coot 0.9, PyMOL ver 2.0.6, RELION-3.1, UCSF chimera 1.12.1 and 1.13.1, AutoDock Vina 1.1.2., Prism 9, Multiwfn 3.8

For manuscripts utilizing custom algorithms or software that are central to the research but not yet described in published literature, software must be made available to editors and reviewers. We strongly encourage code deposition in a community repository (e.g. GitHub). See the Nature Portfolio guidelines for submitting code & software for further information.

Data

Policy information about availability of data

All manuscripts must include a data availability statement. This statement should provide the following information, where applicable:

- Accession codes, unique identifiers, or web links for publicly available datasets
- A description of any restrictions on data availability
- For clinical datasets or third party data, please ensure that the statement adheres to our policy

The authors declare that the main data supporting the findings of this study are available within the article and its Supplementary Information file. Original data can be obtained from the corresponding authors on reasonable request. The coordinates and the structure factor amplitudes for the apo structure of TvTS-TC and for the structure of TvTS-TC after soaking with 2,3-dihydro-HexPP were deposited under accession codes 7VTA and 7VTB, respectively. The cryo-EM maps and the atomic coordinates for MpMS-PT domain and MpMS-crosslink have been deposited in Electron Microscopy Data Bank (EMDB, https://www.ebi.ac.uk/pdbe/emdb/) and PDB with accession codes EMD-32531 and EMD-32532, and 7WIJ, respectively. The accession numbers (NMDCN0000RG9, NMDCN0000RGA, NMDCN0000RGB,

NMDCN0000R73, KFX89132, KAH9237577, KIK55704, KAF2708718, CRG86078, QIH97829) of PTTC candidates for AlphaFold2 prediction were deposited in the National Microbiology Data Center (https://nmdc.cn/en) and listed in supplementary information.

Field-specific reporting

Please select the one below that is the best fit for your research. If you are not sure, read the appropriate sections before making your selection.

☒ Life sciences ☐ Behavioural & social sciences ☐ Ecological, evolutionary & environmental sciences

For a reference copy of the document with all sections, see nature.com/documents/nr-reporting-summary-flat.pdf

Life sciences study design

All studies must disclose on these points even when the disclosure is negative.

Sample size	Quantitative assays were performed in at least three independent biological replicates and mean and standard deviation values calculated. Such sample size was sufficient to determine the enzyme variant activity according to previous publications in a similar field.
Data exclusions	No data was excluded from the manuscript.
Replication	Reproducibility was verified by performing three or more independent biological replicates and noted. All attempts at replication were successful.
Randomization	No randomization was performed during this study as it was not applicable for our experiments. Detection of triperpene products were run consecutively on the GC-MS to minimize instrument drift within each sample. Single protein crystal structures were solved and randomization is not applicable to this section.
Blinding	No blinding was involved in this study as it does not involve animal or human subjects or group allocation. No class I triterpenes has been reported before, and data was collected and analyzed using software in an objective manner. No data was excluded from the analyses, so blinding is less relevant in this work. All data were analyzed and checked by multiple authors and reviewed by the corresponding author.

Reporting for specific materials, systems and methods

We require information from authors about some types of materials, experimental systems and methods used in many studies. Here, indicate whether each material, system or method listed is relevant to your study. If you are not sure if a list item applies to your research, read the appropriate section before selecting a response.

Materials & experimental systems

n/a	Involved in the study
☒	☐ Antibodies
☒	☐ Eukaryotic cell lines
☒	☐ Palaeontology and archaeology
☒	☐ Animals and other organisms
☒	☐ Human research participants
☒	☐ Clinical data
☒	☐ Dual use research of concern

Methods

n/a	Involved in the study
☒	☐ ChIP-seq
☒	☐ Flow cytometry
☒	☐ MRI-based neuroimaging
